# Comparative Genetic Mapping and Discovery of Linkage Disequilibrium Across Linkage Groups in White Clover (*Trifolium repens* L.)

**DOI:** 10.1534/g3.112.002600

**Published:** 2012-05-01

**Authors:** Sachiko N. Isobe, Hiroshi Hisano, Shusei Sato, Hideki Hirakawa, Kenji Okumura, Kenta Shirasawa, Shigemi Sasamoto, Akiko Watanabe, Tsuyuko Wada, Yoshie Kishida, Hisano Tsuruoka, Tsunakazu Fujishiro, Manabu Yamada, Mistuyo Kohara, Satoshi Tabata

**Affiliations:** * Department of Plant Genome Research, Kazusa DNA Research Institute, Kisarazu, Chiba 292-0818, Japan; † Forage Crop Breeding Research Team, National Agricultural Research Center for Hokkaido Region, Sapporo, Hokkaido 062-8555, Japan

**Keywords:** comparative map, white clover, linkage disequilibrium, expressed sequence tag–simple sequence repeat

## Abstract

White clover (*Trifolium repens* L.) is an allotetraploid species (2n = 4X = 32) that is widely distributed in temperate regions and cultivated as a forage legume. In this study, we developed expressed sequence tag (EST)–derived simple sequence repeat (SSR) markers, constructed linkage maps, and performed comparative mapping with other legume species. A total of 7982 ESTs that could be assembled into 5400 contigs and 2582 singletons were generated. Using the EST sequences that were obtained, 1973 primer pairs to amplify EST-derived SSR markers were designed and used for linkage analysis of 188 F_1_ progenies, which were generated by a cross between two Japanese plants, ‘273-7’ and ‘T17-349,’ with previously published SSR markers. An integrated linkage map was constructed by combining parental-specific maps, which consisted of 1743 SSR loci on 16 homeologous linkage groups with a total length of 2511 cM. The primer sequences of the developed EST-SSR markers and their map positions are available on http://clovergarden.jp/. Linkage disequilibrium (LD) was observed on 9 of 16 linkage groups of a parental-specific map. The genome structures were compared among white clover, red clover (*T. pratense* L.), *Medicago truncatula*, and *Lotus japonicus*. Macrosynteny was observed across the four legume species. Surprisingly, the comparative genome structure between white clover and *M. truncatula* had a higher degree of conservation than that of the two clover species.

White clover (*Trifolium repens* L.) is widely distributed in temperate regions of the world and is cultivated as a forage legume mainly in grazing pasture. It propagates both by seeds and stolons, and it grows well in a wide range of soil and environmental conditions (Frame 2005). White clover is an allotetraploid species (2n = 4X = 32) (Atwood and Hill 1940). The putative diploid progenitors are *T. occidentale* and *T. pallescens* (Ellison *et al.* 2006). White clover has an outcrossing reproduction system and exhibits strong gametophytic self-incompatibility, which causes high levels of genetic heterozygosity in natural and synthetic populations (Baker and Williams 1987). The polyploidy and wide genetic diversity in white clover have made it difficult to achieve rapid advances in genetic and genomic studies.

Molecular genetics studies of white clover have been conducted during the past quarter century. In the initial stage, segregation and diversity analyses were performed with restriction fragment length polymorphism (RFLP), random amplified polymorphic DNA (RAPD), and amplified fragment length polymorphism (AFLP) markers (Hughes *et al.* 1990; Gustine and Sanderson 2001; Kölliker *et al.* 2001a; Gustine *et al.* 2002). In later work, a total of 117 and 677 simple sequence repeat (SSR) markers were developed by Kölliker *et al.* (2001b) and Barrett *et al.* (2004), respectively. The first linkage map was constructed with 78 SSR and 57 AFLP markers using an F_2_ mapping population cross between parental inbred lines (Jones *et al.* 2003). Subsequently, a linkage map suggesting homeologous pairing of linkage groups was reported by Barrett *et al.* (2004), which consisted of 493 SSR loci on 16 homeologous linkage groups of 1144 cM. Although several linkage maps were reported after this map (Zhang *et al.* 2007; Wang *et al.* 2010; Casey *et al.* 2010), it continues to be the densest linkage map published to date. Single nucleotide polymorphism (SNP) discovery has been considered a challenging task in white clover because of its allotetraploidy. Despite this difficulty, primers for 58 SNP markers and SNP sites on 20 candidate gene sequences relating to stress tolerance are reported by Cogan *et al.* (2006) and Hand *et al.* (2008).

Along with the advance of linkage maps, quantitative trait loci (QTL) mapping has been progressing mainly for marker-assisted selection (MAS) in white clover. Significant QTLs were identified for seed production (Barrett *et al.* 2005), for 11 morphological and reproductive traits (Cogan *et al.* 2006), and for salt stress tolerance (Wang *et al.* 2010). In addition to QTL, qualitative trait loci were mapped onto linkage groups of white clover, including the red leaf locus (*R* locus) and the self-incompatibility locus (*S* locus) (Barrett *et al.* 2004; Casey *et al.* 2010; Tashiro *et al.* 2010). Although these results suggest the availability of MAS or the possibility of map-based cloning of genes, obvious progress toward these goals is not reported. The lack of a sufficient number of polymorphic DNA markers and a saturated linkage map could prevent the progress of molecular genetics in white clover.

Comparative genetic mapping is an effective strategy for sharing genetic and genomic information between model species and those with more complex genome structures (Feuillet and Keller 2002). White clover is a member of Trifolieae, which includes the genera *Trifolium*, *Medicago*, and *Melilotus* (Gilett 1985). Of the tribe Trifolieae, the genus *Trifolium* is the largest and contains approximately 255 species (Zohary and Heller 1984; Ellison *et al.* 2006). The comparative genetic mapping of white clover was performed first by using red clover (*T. pretense* L.) and alfalfa (*Medicago sativa*) with 167 and 37 commonly mapped markers, respectively (Zhang *et al.* 2007). The result suggested the existence of putative macro-colinearity between the genomes of the two species. A further analysis was performed between white clover and the model legume *Medicago truncatula*, which has a basic chromosome number that is the same as white clover (2n = 2X = 16) (George *et al.* 2008). A total of 159 ESTs mapped on a white clover linkage map showed significant synteny with the genome of *M. truncatula*. This result indicated the predominant colinearity between most of the homeologous groups (HG) of white clover and chromosomes of *M. truncatula*, except for F and H in white clover and chromosomes 2 and 6 in *M. truncatula*. Before this report, linkage groups of the white clover map were named using letters (A–H). George *et al.* (2008) renamed the linkage groups according to the syntenic chromosome number of *M. truncatula*.

To accelerate the advance of molecular genetics in white clover, we performed EST-SSR marker development and constructed an integrated high-density linkage map. For broadening the knowledge across legume species, the genome structure of white clover was compared with that of red clover and two model legumes, *M. truncatula* and *Lotus japonicus*. The resulting EST-SSR markers, integrated linkage map, and observed macrosynteny produced by this work will be valuable resources for genetic mapping, QTL analysis, and molecular breeding of white clover in the future.

## Materials and Methods

### Plant materials

An integrated genetic linkage map was constructed using a full-sib mapping population of 188 individuals derived from a cross between ‘273-7’ as a female parent and ‘T17-349’ as a male parent. ‘273-7’ is a wild accession in the Hokkaido region in Japan, with characteristics of early flowering, large leaves, a red leaf mark, and a sparse stolon network. ‘T17-349’ was derived from ‘Hokkai 1,’ a breeding line of the National Agricultural Research Center for Hokkaido Region (Japan). ‘Hokkai 1’ was bred by a maternal line selection method, which consisted of 10 maternal lines generated in eight countries (United States, Turkey, Afghanistan, Iraq, Switzerland, France, Spain, and New Zealand). ‘T17-349’ was selected for its special characteristics of micro-leaves, late flowering, a white leaf mark, and a dense stolon network.

### Development of EST-SSR markers

Total RNAs were extracted from 68 g of seedlings of a Swedish white clover variety ‘Sonja’ using the Plant RNA Purification Reagent (Invitrogen). The seeds were sown on petri dishes containing moistened filter papers, and whole seedlings were used for RNA extraction when primary leaves were fully developed. Purification of polyadenylated RNA and conversion to cDNA were performed as described previously (Asamizu *et al.* 1999). The synthesized cDNA was resolved by 1% agarose gel electrophoresis, and a fraction ranging from 1 to 3 kb was recovered. The recovered fragments were cloned into the *Eco* RI-*Xho* I site of a pBluescript II SK(‑) plasmid vector (Stratagene) and introduced into an *E. coli* ElectroTen-Blue strain (Stratagene) by electroporation. For generation of ESTs, plasmid DNA was amplified from the colonies using TempliPhi (GE Healthcare) and was subjected to sequencing using the BigDye Terminator Cycle Sequencing Kit (Applied Biosystems). The reaction mixtures were run on the automated DNA sequencer ABI PRISM 3730 (Applied Biosystems).

Sequencing chromatograms were evaluated with PHRED (Ewing *et al.* 1998; Ewing and Green 1998) and vector-derived sequences were trimmed with CROSSMATCH (Ewing and Green 1998). The EST reads were quality-trimmed by the PHRED quality score at a position where five ambiguous bases (PHRED score under 16) were found within 15 contiguous bases. Reads that comprised >50 bp of contiguous quality were submitted to the DDBJ/EMBL/GenBank databases with the accession numbers FY454661 to FY469874. The PHRAP program was used for clustering of ESTs to identify non-redundant white clover ESTs (Ewing and Green 1998).

A similarity search was performed for non-redundant white clover ESTs using the BLASTX program against protein-encoding genes deduced in the genomes of *Arabidopsis thaliana* (Arabidopsis Genome Initiative 2000), *L. japonicus* (Sato *et al.* 2008), soybean (Schmutz *et al.* 2010), and *M. truncatula* (release 3.0; http://www.medicago.org/genome/). The EST contigs were classified into KOG categories according to the results of BLASTX searches against amino acid sequences in the KOG set (http://www.ncbi.nlm.nih.gov/COG/) (Tatusov *et al.* 2003). These sequence similarities were judged to be significant when the E-value was less than 1e−10.

SSRs ≥15 nucleotides in length, which contained all possible combinations of di-nucleotide (NN), tri-nucleotide (NNN), and tetra-nucleotide (NNNN) repeats, were identified from the non-redundant white clover ESTs using the SSRIT (Simple Sequence Repeat Identification Tool) program (Temnykh *et al.* 2001) for perfect SSRs, and the fuzznuc tool from EMBOSS version 6.1.0 (Rice *et al.* 2000) for SSRs with mismatches. Primer pairs for amplification of SSR-containing regions were designed based on the flanking sequences of each SSR with the assistance of the PRIMER3 program (Rozen and Skaletsky 2000), so that amplified fragment sizes were between 90 and 300 bp in length.

### Amplification of SSR markers and polymorphic analysis

DNA was extracted from young leaves of white clover using DNeasy Plant Mini Kit (Qiagen). A total of 4619 primer pairs of SSR markers, including 1973 SSR markers developed in this study (hereafter WCS markers), 2518 red clover SSR markers (RCS markers) developed by Sato *et al.* (2005), and 128 SSR markers on a published white clover linkage map (Zhang *et al.* 2007; Kölliker *et al.* 2001b; Julier *et al.* 2003; Barrett *et al.* 2004; Sledge *et al.* 2005), were used for comparative polymorphic analysis among the two parents and four randomly selected F_1_ progeny. The names of markers and their sources are listed in Table 1. The primer sequences of WCS markers are listed in Table S1.

**Table 1:** Sources of SSR markers, numbers of markers screened, polymorphic markers, polymorphic ratio, and numbers of mapped markers and loci used for segregation analysis of the ’273-7’ × ’T17-349’ mapping population

Marker Name	Source			Number of Markers Screened	Number of Polymorphic Markers	Polymorphic Ratio (%)	Number of Mapped Markers*^b^*	Number of Mapped Loci*^b^*	Average Number of Mapped Loci/Marker*^b^*
	Species	Sequences	Reference						
WCS	White clover	EST		1973	874	44.3	814	1200	1.5
RCS	Red clover	EST and genomic	Sato *et al.* (2005)	2518	305	12.1	282	412	1.5
prs*^a^*	White clover	EST	Barrett *et al.* (2004)	32	18	56.3	17	20	1.2
ats*^a^*	White clover	Genomic	Barrett *et al.* (2004)	30	27	90.0	27	41	1.5
TRSSR*^a^*	White clover	Genomic	Kölliker *et al.* (2001b)	26	22	84.6	20	26	1.3
MTIC*^a^*	*M. truncatula*	EST	Julier *et al.* (2003)	2	2	100.0	2	3	1.0
AI, AJ, AL, AW, BE, BF, BG, BI, MTBA*^a^*	*M. truncatula*	EST	Sledge *et al.* (2005)	36	28	77.8	25	38	1.5
MT1*^a^*	*M. truncatula*	BAC	Sledge *et al.* (2005)	2	2	100.0	2	3	1.5
Subtotal*^a^*				128	99	77.3	93	131	1.4
Total				4619	1278	27.7	1189	1743	1.5

a On white clover linkage map in Zhang *et al.* (2007).

b On an integrated linkage map.

PCR amplification was performed in 5-µl reaction volumes using 0.6 ng of genomic DNA in 1X PCR buffer (Bioline), 3 mM MgCl_2_, 0.08 U of BIOTAQ DNA polymerase (Bioline), 0.8 mM dNTPs, and 0.4 µM of each primer. A modified touchdown PCR protocol was followed as described by Sato *et al.* (2005). The PCR products were separated by electrophoresis using 10% polyacrylamide gels. The primer pairs giving polymorphisms among the mapping parents and/or F_1_ progenies were selected and used for segregation analysis of a mapping population of 188 progenies.

### Linkage analysis

Segregation data obtained from a mapping population of 188 progeny were analyzed by a combination of a color map method (Kiss *et al.* 1998), which employed a comparison of graphical genotypes for mapping, and the JoinMap program v.4 (Van Ooijen 2006). Generally, the procedure for linkage map construction is two steps: grouping and ordering. In this study, the Grouping module of the JoinMap program did not give a reliable result. Therefore, the grouping step was performed carefully as described below.

First, segregated marker loci were categorized into two parental-specific data sets by comparison of the sizes of polymorphic bands of parents and progenies. The segregation data were re-scored using the ‘HAP1’ population type codes employed in the JoinMap analysis. Next, the segregated marker loci in each parental-specific data set were roughly classified into 16 linkage groups using the color map method. Then, the robustness of the data sets for each linkage group was confirmed by the Grouping module of JoinMap using a logarithm of odds (LOD) threshold of 10. Finally, homeologous linkage groups within each parental-specific data set and corresponding linkage groups between the two parental-specific data sets were assumed by comparison of the names of bi-parental and multiple polymorphic marker loci. The locus orders in each parental-specific map were calculated by a Regression Mapping module of JoinMap. Each parental-specific data set was handled as a ‘HAP1’ population type, and the following parameters were used for the calculation: Kosambi’s mapping function, LOD ≥ 1.0, REC frequency ≤ 0.4, goodness-of-fit jump threshold for removal of loci = 5.0, number of added loci after which to perform a ripple = 1, and third round = yes.

After this procedure, the linkage map derived from the ‘273-7’-specific data were considered reasonable, whereas an error persisted in the ‘T17-349’-specific data. This error was because one of the 16 linkage groups of the ‘T17-349’-specific data consisted of an extremely larger number of marker loci (32.4% of the total number of marker loci) than the other 15 groups, and corresponding marker loci classified to the largest linkage group were found on most of the linkage groups of the ‘273-7’-specific data. In addition, graphical genotypes of the largest linkage group showed mosaic patterns (Figure S1). Therefore, we concluded that the loci belonging to multiple linkage groups were not correctly integrated into the largest linkage group. Meanwhile, most of the 16 linkage groups of the ‘273-7’-specific data showed one-on-one colinearity to the eight chromosomes in *M. truncatula* by comparative analysis. Therefore, the marker loci consisting of the largest linkage group of the ‘T17-349’-specific data were carefully disassembled into multiple linkage groups according to the classified groups of the corresponding markers of the ‘273-7’-specific data and chromosomes of *M. truncatula*, as well as their segregation pattern by color mapping. Finally, the reclassified linkage groups were confirmed again based on their robustness under the Grouping module of the JoinMap program.

For construction of an integrated linkage map, parental-specific data sets were integrated into one data set by the Combine Groups for Mapping Integration module, and then ordered by the Regression Mapping module of JoinMap. The parameters used for the mapping module of an integrated map were the same as for the parental-specific maps.

The GGT 2.0 program was employed to determine LD between loci mapped on the parental-specific maps and to draw graphical genotypes (Berloo 2008). To decrease the calculation volume, marker loci were selected on the linkage maps approximately every 5 cM to determine the R^2^, which is one of the indexes of LD. The total numbers of marker loci used for ‘273-7’- and ‘T17-349’-specific maps were 428 and 401, respectively. The names of marker loci used for the calculation are listed in Table S2.

### Comparative mapping

To compare the genome structures of white clover and red clover, SSR markers developed in this study and those on a published white clover linkage map (Zhang *et al.* 2007) were mapped on an integrated linkage map of red clover. A full-sib mapping population of 188 individuals derived from a cross between ‘HR’ and ‘R130’ was used for map construction (Sato *et al.* 2005). Accession-specific maps of ‘HR’ and ‘R130’ were previously reported with 997 and 810 marker loci, respectively, including SSR, AFLP, and RFLP (Isobe *et al.* 2009). Polymorphic analyses were performed between ‘HR’ and ‘R130’ with 1973 WCS and 128 SSR markers (Table 1). The methods for amplification of SSR markers and linkage analysis were the same as described above. An integrated linkage map of red clover was developed by combining the two parental-specific segregation data sets obtained in this and a previous study (Isobe *et al.* 2009). In addition to reconstruction of a red clover linkage map, integration of homeologous linkage groups of the white clover linkage map was performed by the Combine Groups for Mapping Integration module of JoinMap v.4. The cDNA sequences adjacent to the mapped EST-SSR markers on white and red clover maps were compared by BLASTN, and an E-value of less than 1e−20 was considered significant.

Syntenic regions between the genomes of white clover, red clover, and two model legumes, *M. truncatula* and *L. japonicas*, were detected by identifying the conservation of relative location of genes and genomic regions. The sources of genome sequences of the two model legumes were described previously in the section *Development of EST-SSR Markers*. The cDNA sequences adjacent to the mapped EST-SSR markers on the white clover map were compared with the gene sequences in the reference genomes using BLASTX program with a cutoff E-value ≤ 1e−10. A synteny block was defined as the region where three or more conserved homologs were located within a 10-cM region in the white and red clover linkage map, and a 500-kb DNA stretch in the reference genomes. The syntenic regions were plotted using Cicros (http://circos.ca/).

## Results

### Features of white clover ESTs

A total of 15,214 cDNA clones were sequenced from their 5′ ends, and a total of 10,290,123 qualified bases, of which the average GC content was 42.8%, were obtained. To identify the number of independent EST species, clustering of the EST sequences was performed using the PHRAP program. As a result, 7982 potential non-redundant EST sequences were generated, including 5400 contigs and 2582 singletons. When these non-redundant EST sequences were searched for similarity against proteome databases of three legume genomes (soybean, *L. japonicas*, and *M. truncatula*) and the *A. thaliana* genome, 7082 non-redundant ESTs had significant similarity (E-value < 1e−10) to the registered sequences, whereas the remaining 900 ESTs were not previously identified as sequences that show significant similarity on the published genomes for the four reference species (Table S3).

To investigate the functional classification of white clover ESTs, non-redundant EST sequences were compared with the eukaryotic clusters of orthologous groups (KOGs) by BLASTX and classified into KOG categories of assigned orthologous groups (Tatusov *et al.* 2003). Of 7982 non-redundant white clover EST sequences, 5193 showed similarity to KOG sequences with functional classifications. The distribution of non-redundant white clover EST sequences assigned to KOG functional categories is shown in Figure S2.

### SSR features and marker development

A total of 1266 di-, tri-, and tetra-nucleotide SSRs that were ≥15 bp were identified in the non-redundant EST sequences. Provided that the total size of the non-redundant EST sequences is 6.0 Mbp, the frequency of occurrence of the SSRs in transcribed regions of the white clover genome was estimated to be one SSR in every 4.7 kb. Di-, tri-, and tetra-nucleotide SSRs accounted for 19.0%, 69.8%, and 11.1% of the identified SSRs, respectively (Table 2). The SSR motifs on the previously reported 32 EST-SSR markers [prs markers, Barrett *et al.* (2004)] consisted of 1 di-, 24 tri-, 4 tetra-, and 3 penta-nucleotide repeats. The main difference between the study of Barrett *et al.* (2004) and our study was the inclusion of penta-nucleotide repeats by Barrett *et al.* (2004). Among the assigned 1266 SSR regions, qualified primer pairs could be designed on 241 SSR regions. These primer pairs were employed for validation of EST-SSR markers. In addition to the perfect SSR, “mismatch SSRs” were identified, which are SSRs showing 1- or 2-bp mismatch sequences to the repeat motif on the identified SSRs. The numbers of identified 1- and 2-bp mismatch SSRs were 1440 and 4316, respectively. To increase the numbers for the candidate EST-SSR markers, an additional 456 and 1276 primer pairs were designed on the SSR regions that allowed the presence of 1- and 2-bp mismatch motifs in the SSRs, respectively (Table 2). As a result, a total of 1973 EST-SSR markers were designed and named WCS markers.

**Table 2:** SSRs in non-redundant white clover ESTs and designed EST-SSR primers

SSR Pattern	SSR Numbers in Non-redundant White Clover ESTs	Frequency (%)	Designed EST-SSR Primers			
			Mismatch 0	Mismatch 1	Mismatch 2	Total
AG	211	16.67	30	19	44	93
AT	22	1.74	7	3	15	25
AC	8	0.63	4	6	11	21
GC	0	0.00	0	0	0	0
AAC	182	14.38	49	64	139	252
AAG	179	14.14	35	90	246	371
GGT	170	13.43	27	63	157	247
ATC	113	8.93	30	49	151	230
AAT	86	6.79	17	21	66	104
AGC	79	6.24	8	33	90	131
GGA	36	2.84	10	34	89	133
GGC	19	1.50	2	8	47	57
ACT	16	1.26	5	12	21	38
ACG	4	0.32	0	5	21	26
AATG	26	2.05	3	12	19	34
AAAT	23	1.82	4	13	30	47
AAAG	20	1.58	3	5	44	52
AGTG	8	0.63	0	0	0	0
ATAG	8	0.63	0	0	0	0
AATT	8	0.63	3	2	17	22
ATAC	5	0.39	0	0	0	0
AACC	4	0.32	0	0	0	0
AAAC	4	0.32	0	9	34	43
AACT	3	0.24	0	0	0	0
Other tetra-nucleotide repeats	32	2.53	4	8	35	47
Total	1266	100	241	456	1276	1973

In terms of the repeat motifs among the 1973 generated WCS markers, 1589 (81%) were tri-nucleotide repeats, whereas 139 (7%) and 245 (12%) were di- and tetra-nucleotide repeats, respectively. Three types of di-nucleotide repeats were observed, with poly (AG)n the most frequently observed, constituting 67% of the di-nucleotide repeats (Table 2). Among the tri-nucleotide repeats, the poly (AAG)n motif was most abundant (371, 23.3%), followed by poly (AAC)n (252, 15.9%), poly (GGT)n (247, 15.5%), and poly (ATC)n (230, 14.5%). The tetra-nucleotide repeats, specifically, poly (AAAG)n, poly (AAAT)n, poly (AAAC)n, and poly (AATG)n, were more commonly observed compared with other motifs, and collectively they added up to 72% of the tetra-nucleotide repeats. The details of the designed white clover EST-SSR primers, along with the corresponding SSR motif, product size, primer sequence, and images of 10% polyacrylamide gel electrophoresis, are provided on the web database at http://clovergarden.jp/ and in Table S1.

### Construction of parental-specific linkage maps

Polymorphisms of the white clover mapping population were examined for a total of 4619 SSR markers, including 1973 white clover SSR markers (WCS), 2518 red clover SSR markers (RCS), and 128 other SSR markers on a previously published white clover linkage map (Zhang *et al.* 2007) (Table 1). As a result, 874, 305, and 99 polymorphic markers were screened in WCS, RCS, and other markers. The polymorphic ratio of WCS markers was 44.3%, whereas that of RCS markers was 12.1%. A total of 1797 polymorphic loci were identified from 1278 markers (data not shown). Multiple loci generated from single markers were indicated by attaching lowercase letters after the marker names (*e.g.* WCS0403a and WCS0403b). Of the 1797 loci, 424, 789, and 584 showed bi-parental, ‘273-7’-specific, and ‘T17-349’-specific polymorphisms, respectively.

Of the 1173 loci showing polymorphisms on ‘273-7’, 1059 were mapped onto 16 linkage groups, representing a total map length of 2322.9 cM (Table 3 and Table S2). The length of each linkage group ranged from 97.6 cM (LG6a) to 186.5 cM (LG1b). The mean locus density and segregation distortion were 2.19 cM and 35.7%, ranging from 1.43 cM·locus^-1^ (LG8a) to 4.07 cM·locus^-1^ (LG6a) and from 19.5% (LG3a) to 66.0% (LG2b), respectively. Most of the linkage groups, except LG6a and LG6b, showed colinearity to eight chromosomes of *M. truncatula* (data not shown). Therefore, the linkage group numbers were designated based on the corresponding *M. truncatula* chromosome number. The numbers of loci showing significant similarity with genome sequences on the corresponding *M. truncatula* chromosomes were 22 (LG1a, 41% of the total number of mapped loci), 32 (LG1b, 39%), 17 (LG2a, 31%), 20 (LG2b, 43%), 37 (LG3a, 45%), 54 (LG3b, 53%), 52 (LG4a, 52%), 32 (LG4b, 46%), 37 (LG5a, 58%), 33 (LG5b, 70%), 1 (LG6a, 4%), 6 (LG6b, 11%), 18 (LG7a, 47%), 36 (LG7b, 50%), 43 (LG8a, 48%), and 35 (LG8b, 45%). Homeologous linkage groups were distinguished by randomly attached lower case letters (*e.g.* ‘a’ and ‘b’).

**Table 3:** Description of parental-specific and integrated maps

LG	‘273-7’-specific Map					‘T17-349’-specific Map			
	Number of Loci	Length (cM)	Density*^a^* (cM per marker)	Segregation Distortion Ratio (%)^b^		Number of Loci	Length (cM)	Density*^a^* (cM per marker)	Segregation Distortion Ratio (%)*^b^*
1a	54	153.0	2.83	50.0		63	153.8	2.44	71.4
1b	83	186.5	2.25	53.0		37	178.5	4.82	59.5
2a	55	122.3	2.22	34.5		62	197.6	3.20	58.1
2b	47	159.0	3.38	66.0		58	179.7	3.10	36.2
3a	82	150.0	1.83	19.5		51	154.5	3.03	37.3
3b	101	147.4	1.46	28.7		63	107.5	1.71	52.4
4a	100	158.3	1.58	26.0		62	129.4	2.09	75.8
4b	70	139.5	1.99	28.6		76	155.5	2.05	40.8
5a	64	138.2	2.16	29.7		64	142.4	2.22	35.9
5b	47	149.6	3.18	34.0		64	175.0	2.73	50.0
6a	24	97.6	4.07	41.7		22	120.5	5.48	54.6
6b	55	140.0	2.54	36.4		16	124.3	7.77	50.0
7a	38	129.3	3.40	57.9		56	252.3	4.51	44.6
7b	72	174.5	2.42	27.8		44	115.4	2.62	72.7
8a	89	126.8	1.43	31.5		85	174.5	2.05	60.0
8b	78	150.7	1.93	39.7		40	89.6	2.24	57.5
Total	1059	2322.9	2.19	35.7		863	2450.3	2.84	53.3
LG	Integrated Map								
	Number of Loci	Length (cM)	Density*^a^* (cM per marker)	Segregation Distortion Ratio (%)*^b^*					
1a	106	159.1	1.50	57.6					
1b	113	190.9	1.69	62.0					
2a	107	122.9	1.15	45.8					
2b	98	207.8	2.12	50.0					
3a	124	172.5	1.39	28.2					
3b	146	142.6	0.98	41.8					
4a	141	157.0	1.11	51.8					
4b	134	177.1	1.32	35.8					
5a	118	164.5	1.39	35.6					
5b	108	156.2	1.45	39.8					
6a	(44)*^c^*	(109.08)*^c^*	(2.48)*^c^*	(50.0)*^c^*					
6b	63	132.4	2.10	39.7					
7a	86	155.7	1.81	53.5					
7b	101	151.5	1.50	46.5					
8a	146	166.1	1.14	52.1					
8b	108	146.0	1.35	44.4					
Total	1743	2511.3	1.44	45.6					

a Average length between two loci.

b A significant level at *P* < 0.05.

c LG 6a was not integrated because of fewer bi-parental markers. Therefore, the number of locus and length were investigated as follows: number of locus = (total number of the parental-specific maps) – (number of commonly mapped markers on the parental-specific maps); length = average length of the parental-specific maps.

As briefly described in *Materials and Methods*, the grouping procedure of the ‘T17-349’-specific map was more complicated than that of the ‘273-7’-specific map. When we classified a total of 1008 loci showing polymorphisms on ‘T17-349’ onto 16 linkage groups, we found that one of the linkage groups was consistent with a much larger number of loci (327/1008, 32.4%) than the other 15 linkage groups (data not shown). All of the loci of the largest linkage groups were successfully ordered by the JoinMap software, but the result appeared to be incorrect because graphical genotypes of mapping populations showed mosaic patterns (Figure S1). Moreover, the corresponding loci of the linkage map were mapped on most of linkage groups of the ‘273-7’-specific map, and no colinearity was found between the linkage group and chromosomes of *M. truncatula*. Therefore, we concluded that the largest linkage group was consistent with loci that were originally generated from multiple chromosomes and showing similar segregation patterns. For this reason, the loci consisting of the largest linkage group of the ‘T17-349’-specific data were disassembled to multiple linkage groups according to the homeologous linkage groups of the corresponding markers of the ‘273-7’-specific map and chromosomes of *M. truncatula*, along with their segregation pattern by color mapping. As a result, the ‘T17-349’-specific map was constructed with 863 loci of 16 linkage groups, with a total length of 2450.3 cM (Table 3 and Table S2). The graphical genotypes of the individuals showed less inconsistency than the previous map (Figure S3). By comparison of the marker positions between the two parental-specific maps, the linkage groups of the ‘T17-349’-specific map were numbered according to the names of the ‘273-7’-specific map. The numbers of loci showing significant similarity with genome sequences on the corresponding *M. truncatula* chromosomes were 25 (LG1a, 40% of the total number of mapped loci), 14 (LG1b, 38%), 14 (LG2a, 23%), 29 (LG2b, 50%), 27 (LG3a, 53%), 37 (LG3b, 59%), 33 (LG4a, 53%), 44 (LG4b, 58%), 36 (LG5a, 56%), 35 (LG5b, 55%), 3 (LG6a, 14%), 2 (LG6b, 13%), 32 (LG7a, 57%), 24 (LG7b, 55%), 36 (LG8a, 42%), and 11 (LG8b, 28%). The length and locus density of each linkage group of the ‘T17-349’-specific map ranged from 89.6 (LG8b) to 252.3 cM (LG7a) and from 1.71 cM·locus^-1^ (LG3b) to 7.77 cM·locus^-1^ (LG6b), respectively. Segregation distortion (*P* < 0.05) of each linkage group tended to be higher than that of ‘273-7’ and ranged from 35.9% (LG5a) to 75.8% (LG4a), with 53.3% as a mean.

### Construction of an integrated linkage map

An integrated linkage map was constructed by combining the segregation data of the ‘273-7’- and ‘T17-349’-specific maps. Most of the linkage groups were successfully integrated; however, LG6a was not integrated because only two bi-parental loci were commonly mapped on the two parental-specific maps. Therefore, the number of mapped loci and the length of LG6a were calculated as follows. Number of loci = (total number of the parental-specific maps) – (number of commonly mapped markers on the parental-specific maps); length = average length of the parental-specific maps. As a result, 1743 loci generated from 1189 SSR markers were mapped onto 16 linkage groups, which totaled 2511.3 cM in length (Table 3 and Table S2). The length of each linkage group varied from 122.9 (LG2a) to 207.8 cM (LG2b). The locus density and segregation distortion were 1.44 cM·locus^-1^ and 45.6%, ranging from 0.98 cM·locus^-1^ (LG3b) to 2.12 cM·locus^-1^ (LG2b) and from 28.2% (LG3a) to 62.0% (LG1b), respectively.

**Figure 1:**
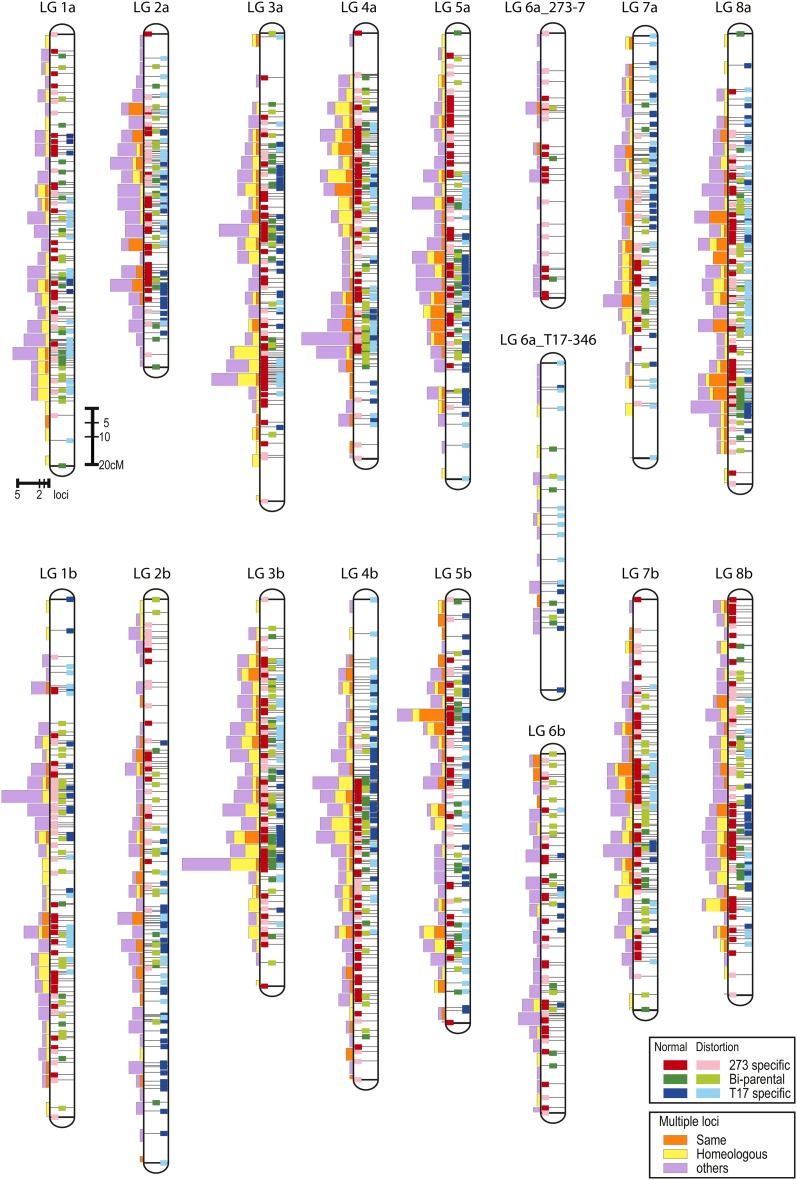
An integrated linkage map of white clover generated from a mapping population of ‘273-7’ × ‘T17-349’. Positions of bi-parental, ‘273-7’-specific, and ‘T17-349’-specific loci are indicated by colors of linkage bars. Those showing normal segregation of bi-parental, ‘273-7’-specific, and ‘T17-349’-specific loci are green, red, and blue, respectively. Those showing distorted segregation of bi-parental, ‘273-7’-specific, and ‘T17-349’-specific loci are yellow-green, pink, and aqua, respectively. Distorted loci are preferentially represented when multiple loci, including the distorted loci, are closely located. Bars attached to linkage groups show numbers of mapped multiple loci per each 5 cM. The orange and yellow colors indicate when all multiple loci generated from a single marker are mapped onto the same or homeologous linkage groups, respectively, whereas purple represents other cases.

The numbers of bi-parental, ‘273-7’-specific, and ‘T17-349’-specific loci mapped onto the integrated linkage map were 407, 764, and 532, respectively (Table S4). Both parental-specific and bi-parental markers mapped randomly onto most regions of the integrated linkage groups, except for several distal regions (*i.e.* top of LG1a, LG2b, LG4b, LG 5a, LG7a, and LG 7b, and bottom of LG1b, LG2b, LG4b, LG5b, and LG6b) (Figure 1). The largest gap between two loci on the integrated linkage groups was 19.1 cM, between RCS3956 and WCS0963a on LG7a.

The number of mapped multiple loci generated from a single marker was 1.5 on average and ranged from 1 to 5 (Table 1 and Table S2). Multiple loci were classified as Type I and Type II (Table S2). Type I was defined as multiple loci identified by observation of multiple bands on 10% polyacrylamide gels. Type II was defined as those generated from single bi-parental segregation data and located on different linkage groups of the parental-specific maps. The total number of multiple loci was 962 (55.3% of all mapped loci), of which the numbers of Type I and Type II were 186 and 836, respectively (60 loci overlapped). Of the 962 multiple loci, 324 and 409 were mapped onto the same or homeologous linkage groups, respectively, whereas 229 multiple loci were mapped onto other linkage groups (Table S4). The multiple loci were identified across all linkage groups (Figure 1), but they did not always map randomly. Homeologous multiple loci were frequently observed on LG3a and LG3b and the upper half of LG4a and LG4b, and less frequently mapped on LG2a and LG2b.

### Detection of LD across linkage groups

**Figure 2:**
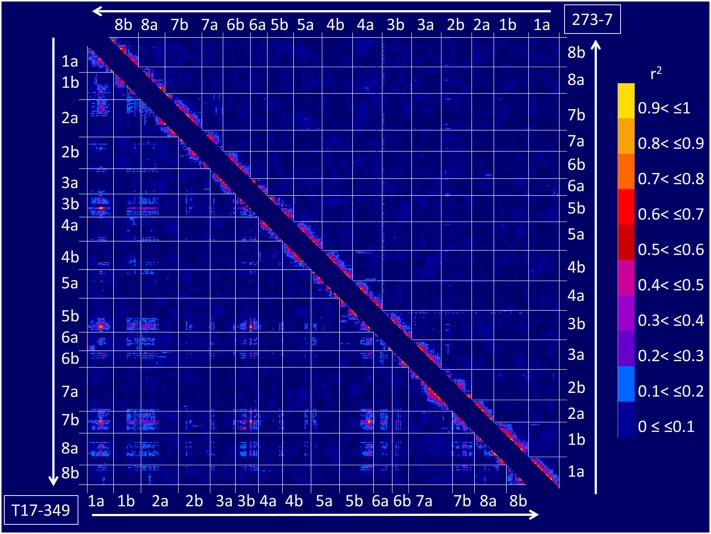
Patterns of LD blocks of the parental-specific maps. Upper right and lower left triangles show LD blocks of ‘273-7’-specific and ‘T17-349’-specific maps, respectively. Colors in triangles indicate magnitudes of LD (r^2^) as listed on the right side.

The genome-wide LD was estimated for the marker loci mapped onto parental-specific linkage maps (Figure 2). High r^2^ values were observed between most of the adjacent marker loci mapped onto each linkage group. When considering marker loci mapped onto different linkage groups, LD showing r^2^ > 0.5 was observed between 87 locus pairs located on LG1a, LG1b, LG2a, LG3b, LG5b, LG6a, LG6b, LG7b, and LG8a of the ‘T17-349’-specific map, whereas r^2^ > 0.5 was identified only between single marker pairs of WCS1722a (LG3b) and WCS0080b(LG7b) of the ‘273-7’-specific map. Of the regions showing significant LD across linkage groups of the ‘T17-349’-specific map, four regions had extremely high LD compared with other regions, including 75–80 cM of LG1a, 55–59 cM of LG3b, 143–156 cM of LG5b, and 54–63 cM of LG7b.

### Comparison with the integrated linkage map of red clover

To investigate genome synteny between white clover and red clover, 2071 SSR markers developed from white clover and *M. truncatula* sequences (Table 1) were examined for mapping onto the red clover linkage map. As a result, 240 loci generated from 238 markers were newly mapped onto an integrated linkage map of red clover developed from a cross between ‘HR’ and ‘R130’ (Table S5). The total number of mapped loci and the genetic length of the map were 1714 and 833.9 cM, respectively. Each of the two homeologous linkage groups of the white clover integrated map were integrated to a single linkage group to simplify the comparison of white and red clover genomes.

**Figure 3:**
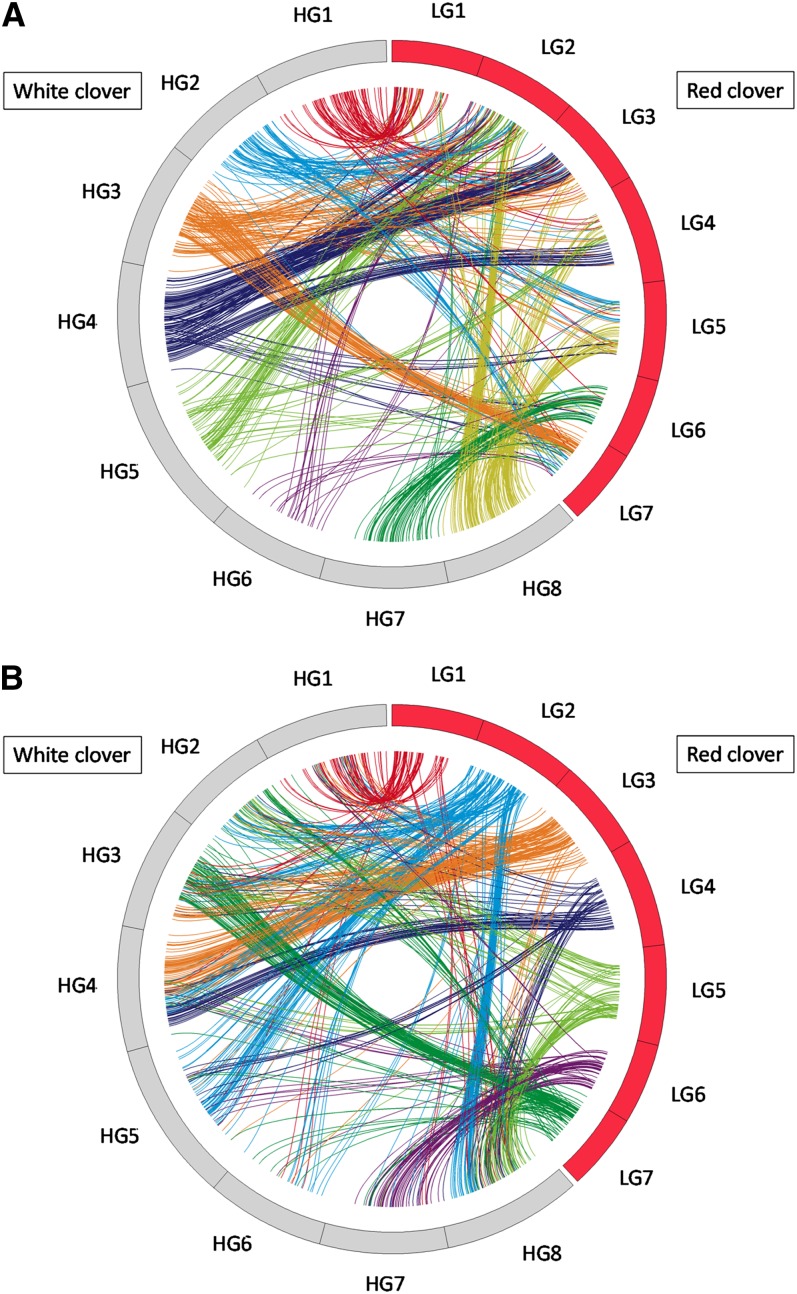
Graphical view of syntenic blocks between white and red clover. Loci generated common or orthologous markers identified by BLASTX searches with a cutoff E-value ≤ 1e−10. (A) Colors of lines represent white clover homeologous linkage groups of orthologous loci. (B) Colors of lines represent red clover linkage groups of orthologous loci.

Of the 1743 loci mapped onto the white clover integrated linkage map, 951 showed sequence similarities to the ESTs or genome sequences of markers mapped onto the integrated linkage map of red clover. As shown in Figure 3, alignment of homologs along each linkage group revealed an obvious syntenic relationship. The proposed syntenic regions were white clover (wc) HG1–red clover (rc) LG1; wc HG2–rc LG2, LG5, and LG7; wc HG3–rc LG2, LG3, and LG7; wc HG4–rc LG2, LG3, and LG4; wc HG5–rc LG2 and LG4; wc HG6–rc LG2 and LG7; wc HG7–rc LG6; and wc HG8–rc LG2, LG4, and LG5.

### Comparison with the genomes of two model legumes, *M. truncatula* and *L. japonicus*

**Figure 4:**
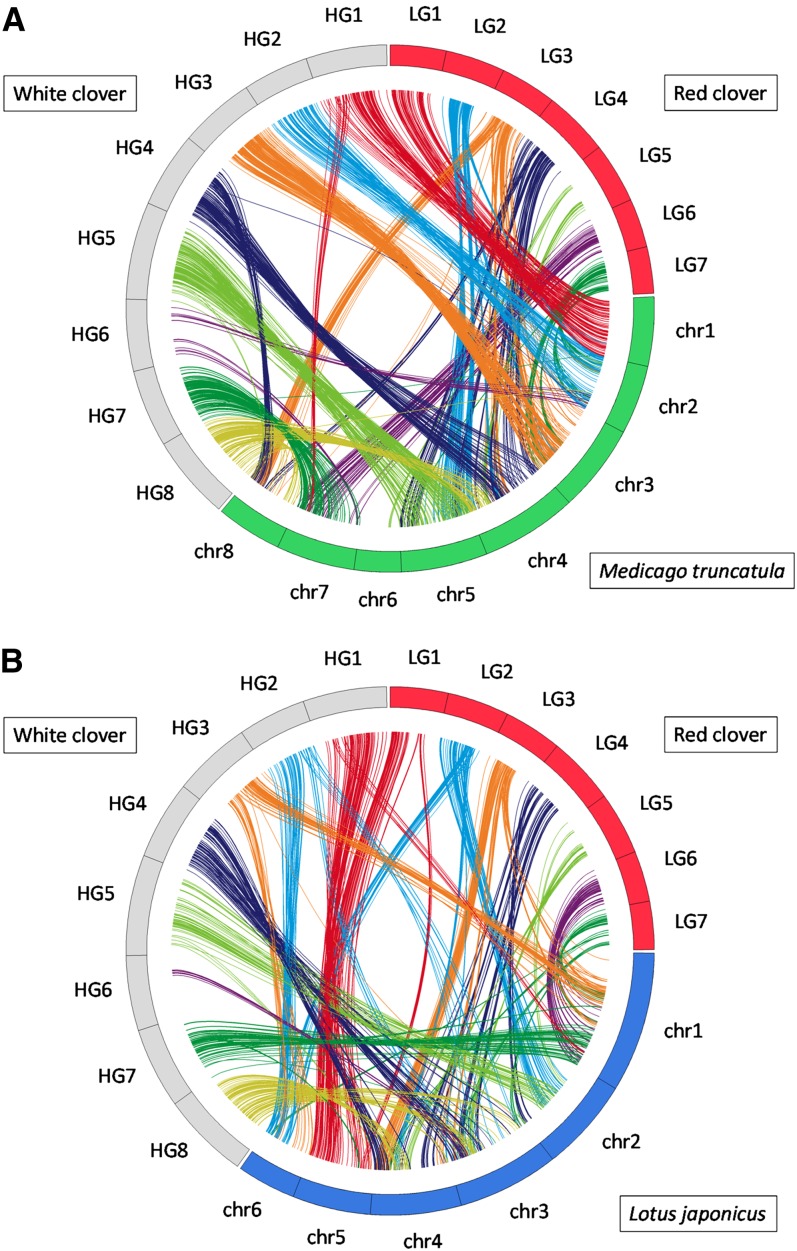
Graphical view of syntenic relationship between white clover, red clover, *M. truncatula*, and *L. japonicus*. Homologous regions were identified by BLASTX searches with a cutoff E-value ≤ 1e−10. Synteny blocks were defined as the region where three or more conserved homologs were located within a 10-cM region in the white and red clover linkage maps, and a 500-kb DNA stretch in the reference genomes. Syntenic regions between the two clover species and model legumes are connected by colored lines. Line colors represent white clover homeologous linkage groups and red clover linkage groups of orthologous loci. (A) Syntenic relationships between two clover species and *M. truncatula*. (B) Syntenic relationships between two clover species and *L. japonicus.*

Of the 1096 sequences that corresponded to mapped SSR markers, 784 and 807 showed sequence similarities to the genes of *M. truncatula*, and *L. japonicus*, respectively, and 725 were common to two genomes. By considering the genes with highest similarity score as putative orthologs, the map locations of the white clover markers and the corresponding genes of the other legumes were compared. As shown in Figure 4 and Figure S4, the alignment of homologous sequence pairs along each linkage group revealed an obvious syntenic relationship. Syntenic relationships seemed to be highest against *M. truncatula* (Mt), in which the syntenic relationships spanned whole chromosome between wc HG1–Mt chr1, wc HG2–Mt chr2, wc HG3–Mt chr3, wc HG5–Mt chr5, and wc HG7–Mt chr7. A segmental syntenic blocks were observed between wc HG1–Mt chr7; wc HG4–Mt chr4 and 8; and wc HG8–Mt chr4 and 8. No synteny blocks were observed between white clover HGs and Mt chr6. A segmental level of syntenic relationships were detected against *L. japonicus* (Lj) as follows: wc HG1–Lj chr5; wc HG2–Lj chr6; wc HG3–Lj chr1; wc HG4–Lj chr3 and 4; wc HG5–Lj chr2; wc HG7–Lj chr1; and wc HG8–Lj chr3 and 4.

## Discussion

The original aim of this study was to develop EST-SSR markers and linkage maps for molecular genetics analysis in white clover. We achieved this by generating 1973 WCS markers and an integrated map with 1743 loci. WGS markers were designed based on the accumulated 7982 non-redundant white clover EST sequences. Although the number of accumulated EST sequences was limited, the proportion of KOG functional categories represented by the accumulated EST sequences was comparable to the proportion predicted in the genomes of *Arabidopsis*, *L. japonicas*, and rice. In addition, the distribution of the identified SSR motifs in the accumulated white clover EST sequences was basically similar to the distribution found in the genomes of other legume species, such as *L. japonicas* (Sato *et al.* 2008). Therefore, no specific bias should be observed in the accumulated white clover EST sequences.

In parallel, we discovered the existence of LD across linkage groups on the ‘T17-349’-specific map. Although the segregation pattern of polymorphic bands in an F_1_ population of an outcrossing species is complex, most of the polymorphic bands of loci showing high LDs mapped onto different linkage groups and were clearly resolved (Figure S5). In addition, the patterns of graphical genotypes of those loci were not inconsistent with other markers (Figure S3). Therefore, these data indicate that both the graphical genotypes and the mapping position of loci were adequate. We considered this the most unique result in this study, because linkage analysis has been generally performed under a prerequisite assumption of the absence of linkage between chromosomes. Therefore, it was impossible to identify LD across linkage groups without the assistance of reference information. In this study, we used two reference sources for identification of LD across white clover linkage groups, including another parental-specific map (‘273-7’) and a predominant macrosynteny between *M. truncatula*. This work is an example of the application of comparative genomics to reveal unique molecular genetics phenomena of species that have a complex genome structure. The LD across linkage groups was observed only in the ‘T17-349’-specific map, which suggested that it was a specific behavior of haplotype combination. Based on the experimental procedures and results of this study, it is not possible to specify the biological factors that affect the phenomenon. However, the result suggested that systematic selection was occurring to promote (or avoid) specific combinations of haplotype blocks across chromosomes. Further analysis, such as an investigation of the segregation of haplotypes of BC_1_F_1_ populations derived from a cross between multiple F_1_ and ‘T17-349,’ would be required to determine the biological factors that affect the phenomenon.

Of the 1973 designed primer pairs of WCS markers, 1776 were amplified fragments with DNAs of the ‘273-7’×’T17-349’ F_1_ mapping population (data not shown). To our knowledge, a total of 168 primer sequences, including 90 SSR and 78 SNP markers, are published for DNA markers generated from the white clover genome or EST sequences (Kölliker *et al.* 2001b; Barrett *et al.* 2004; Cogan *et al.* 2006; Hand *et al.* 2008). We expect that the large number of primer sequences published in this study will help advance the study of molecular genetics in white clover.

Forty-four percent of the 1973 WCS markers showed polymorphisms in the F_1_ mapping population. The polymorphic ratio was 2.4 times higher than that observed in polymorphic analyses of RCS markers with a red clover F_1_ mapping population (18.2%) (Sato *et al.* 2005). Meanwhile, the average number of mapped loci generating a single marker in white clover was 1.47, which was 1.4 times higher than that observed in red clover (1.03) (Sato *et al.* 2005). Although both white and red clover have self-incompatibility systems and are considered to maintain high degree of heterozygosity within populations, white clover showed significantly higher polymorphisms than red clover. Considering that red clover is a diploid species, the possible reason is a higher number of multiple loci caused by allotetraploidy of white clover.

The integrated linkage map constructed in this study consisted of 1743 loci on 2511 cM. The length of the linkage map was longer than those previously published: 1144 cM in Barrett *et al.* (2004) and 1877 cM in Zhang *et al.* (2007). The mapped loci were randomly located across linkage groups, and the average interval between loci was 1.4 cM. However, we were unsuccessful in obtaining an integrated linkage group of LG6a because of the low number of bridging loci. Therefore, we concluded that the integrated linkage map was almost saturated except for LG6a. The segregation distortion ratio varied between parental-specific maps as well as among linkage groups. Most of the linkage groups in the ‘T17-349’-specific map showed a higher segregation distortion ratio than those observed in the ‘273-7’-specific map. By contrast, no common pattern was observed for variation of segregation distortion among linkage groups between the parental-specific maps. Therefore, it was concluded that the behavior of segregation distortion in white clover was specific for haplotype combination.

*T. occidentale* and *T. pallescens* are putative diploid progenitors of white clover. In this study, we randomly suffixed ‘a’ and ‘b’ to each homeologous linkage group without consideration of their origin. Casey *et al.* (2010) pointed out a risk of random suffixing, because it would mislead identification of two subgenome groups. We temporarily added suffixes for identification of the 16 linkage groups, but the suffixes were replaced immediately with other suffixes that reflect the progenitor of the subgenomes. Hand *et al.* (2008) demonstrated the identification of progenitors of subgenomes by an *in vitro* gene-associated SNP discovery approach and found close similarity between the genome of *T. occidentale* and the linkage group LG3(A)O. In their study, progenitor-specific SNPs were identified on stress tolerance–related genes. We consider that SNPs on amplicons of mapped EST-SSR markers also can be used for identification of the progenitor of genomes. By application of the approach reported by Hand *et al.* (2008), it would be possible to identify progenitors of all homeologous linkage groups in white clover.

Significant synteny blocks were identified between white clover and red clover. Although the identified synteny blocks in this study showed more complex patterns, the results are consistent with those identified by Zhang *et al.* (2007). George *et al.* (2008) compared genome structures between white clover and *M. truncatula*, and they identified predominant synteny between the two species, except HG2(F) and HG6(H) in white clover and chr2 and chr6 in *M. trancatula*. Although only 14 markers were commonly mapped between the two studies, most of our results agree with George *et al.* (2008). Prominent synteny was observed between wc HG2 and Mt chr2. Translocation was observed between wc HG1 and Mt chr7, wc HG4 and Mt chr8, and wc HG8 and Mt chr4. It is interesting that clearer macrosynteny was observed between white clover and *M. truncatula* than between white and red clover. Because the ancestor chromosome number in the genus *Trifolium* is supposed to be 2n = 16, our results suggest that the genome structures of white clover and *M. truncatula* have not drastically diverged after genus *Trifolium* and *Medicago* diverged (Ellison *et al.* 2006). White clover belongs to the section *Trifoliastum*, which consists of species with a basic chromosome number of 8, whereas red clover belongs to the section *Trifolium*, which consists of species with basic chromosome numbers of 5,6,7,8, and 24 (Ellison *et al.* 2006). Therefore, genome rearrangement of red clover might have happened after the divergence of white clover and red clover into different sections. In our results, HG6 consisted of fewer loci than other HGs, and chromosome 6 of *M. truncatula* did not show significant macrosynteny between two clover species. O’Bleness (2008) described that chromosome 6 of *M. truncatula* was different from other chromosomes: it is the smallest and has multiple heterochromatic sites and retroviral elements scattered throughout its arm. The unique structure of the chromosome might be conserved in white clover HG6 and cause the difficulty of mapping EST-SSR loci.

Along with recent remarkable advances of technologies in genome analysis, knowledge of the genetics and genomics of plant species has rapidly progressed. However, there are still many hurdles in genetic and genomic analysis in polyploid and outcrossing species because of the difficulty in distinguishing between heterozygous, paralogous, and homeologous sequences. There are still many unresolved issues in the genetic analysis of polyploidy and outcrossing species, such as QTL identification, gene expression, and inbreeding depression. We anticipate that the EST-SSR markers and linkage maps developed in this study will accelerate the progress of genetics in white clover and other polyploid and outcrossing species.

## Supplementary Material

Supporting Information
