# Supplementary material for: Comparative Genetic Mapping and Discovery of Linkage Disequilibrium Across Linkage Groups in White Clover (Trifolium repens L.)
Source: G3 (Bethesda). 2012 May 1;2(5):607–17. doi: 10.1534/g3.112.002600 (PMC3362943; doi:10.1534/g3.112.002600)
Supplement: Supporting Information [file supp_2_5_607__index.html]

Supporting Information 

# Comparative Genetic Mapping and Discovery of Linkage Disequilibrium Across Linkage Groups in White Clover (*Trifolium repens* L.)

## Supporting Information for Isobe *et al*, 2012

**Files in this Data Supplement:**

- Supporting Information - Figures S1-S5 and Tables S1-S5 (PDF, 4 MB)
- Figure S1 - Graphical genotypes of the largest linkage groups of the 'T17-349'-specific map constructed before disassembling the locus data (PDF, 181 KB)
- Figure S2 - Functional classification of non-redundant EST sequences of *T. repens* and unigene sequences of *L. japonicus*, *A. thaliana*, and *O. sativa* based on KOG categories (PDF, 330 KB)
- Figure S3 - Graphical genotypes of 50 of the 188 F1 populations mapped onto the largest linkage group shown in Figure S1 (PDF, 1.5 MB)
- Figure S4 - Graphical view of syntenic blocks between white clover and *M. truncatula* (PDF, 164 KB)
- Figure S5 - List of loci with identified LD showing r2>0.5 between loci mapped onto different linkage groups, and photographs of acrylamide gel electrophoresis of PCR amplicons of the listed loci (PDF, 11.6 MB)
- Table S1 - Primer sequences, SSR motifs, and expected sizes of amplicons of white clover EST-SSR markers (.xls, 374 KB)
- Table S2 - Locus name, position, and segregation distortion of the integrated and parental-specific maps (.xls, 480 KB)
- Table S3 - List of the non-redundant white clover ESTs (.xls, 1.8 MB)
- Table S4 - Number of mapped multiple loci and loci exhibiting parental-specific polymorphisms (.xls, 31 KB)
- Table S5 - Locus name and position of an integrated linkage map of red clover developed from a cross between HR and R130 (.xls, 182 KB)
