# Supplementary material for: Comparative Genetic Mapping and Discovery of Linkage Disequilibrium Across Linkage Groups in White Clover (Trifolium repens L.)
Source: G3 (Bethesda). 2012 May 1;2(5):607–17. doi: 10.1534/g3.112.002600 (PMC3362943; doi:10.1534/g3.112.002600)
Supplement: Supporting Information [file supp_2.5.607_002600SI.pdf]

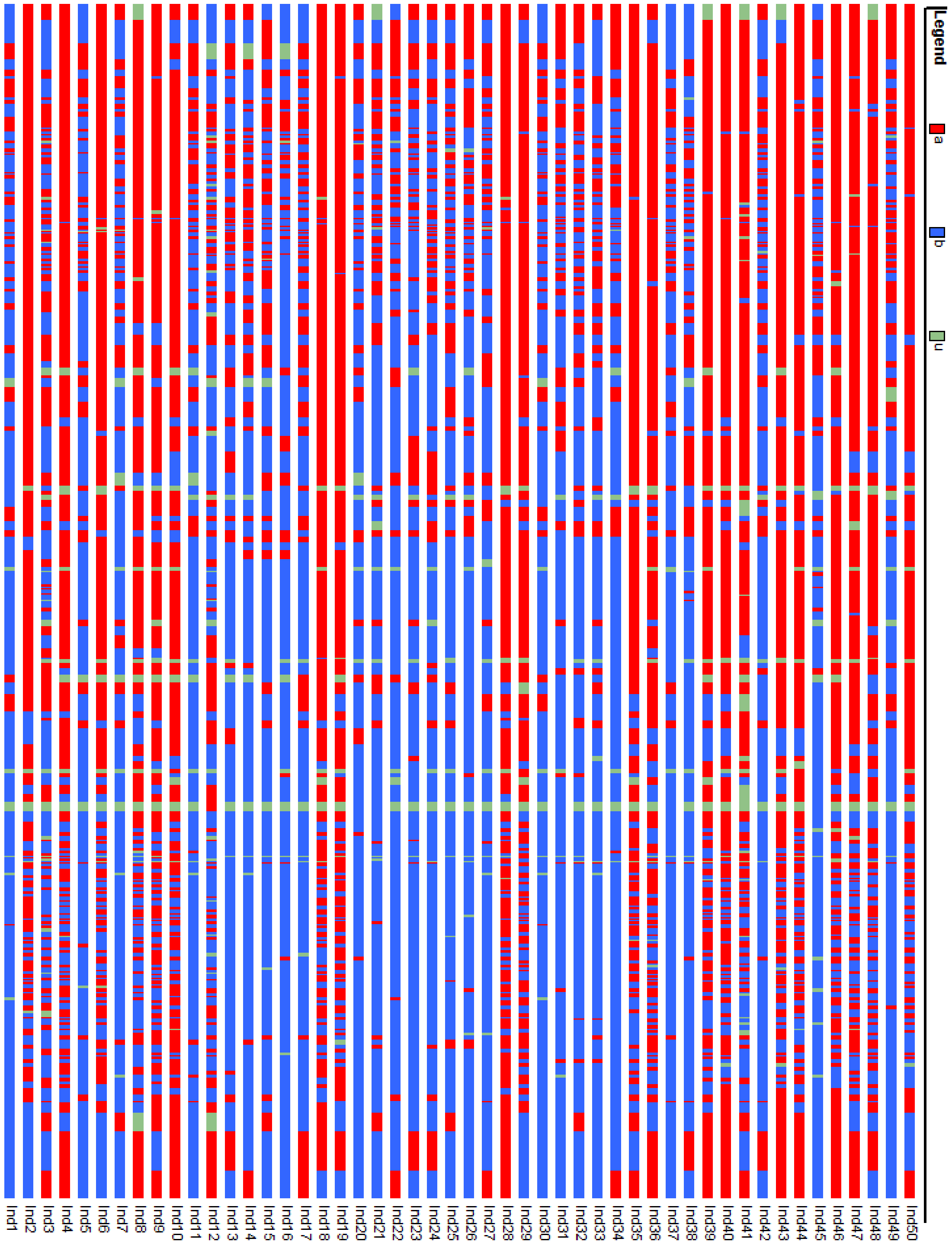

Figure S1 Graphical genotypes of the largest linkage groups of the 'T17-349'-specific map constructed before disassembling the locus data. The linkage group consisted of a total of 296 loci. Graphical genotypes are shown for 50 of the 188 F_1 mapping population. Red and blue colors show the two haplotypes of 'T17-349'. Light green color shows missing data.

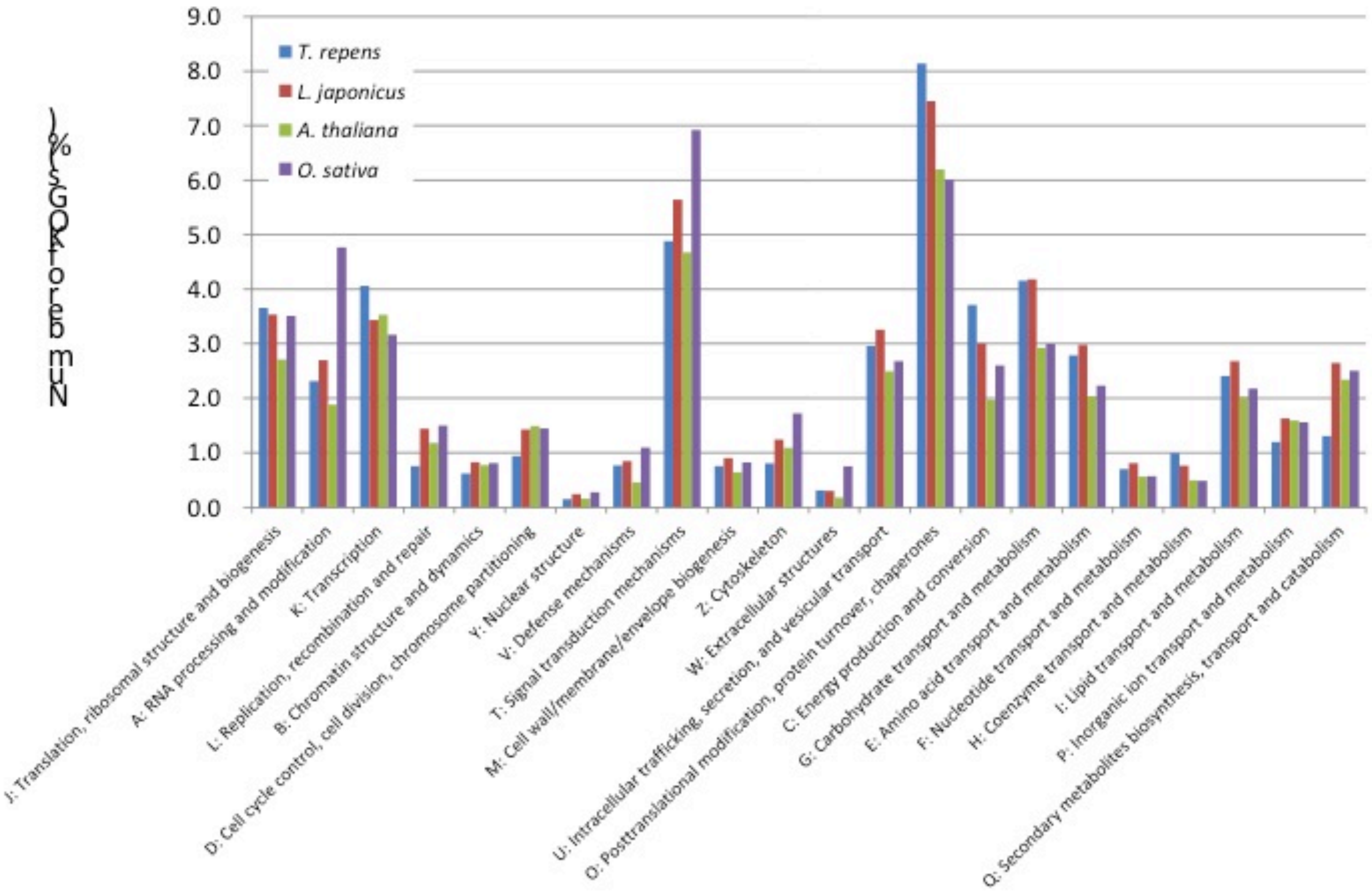

Figure S2 Functional classification of non-redundant EST sequences of *T. repens* and unigene sequences of *L. japonicus*, *A. thaliana*, and *O. sativa* based on KOG categories. BLASTX was used to compare the non-redundant EST sequences with the sequences in the KOG database. EST sequences were then classified into the KOG categories with the most similar sequences. The total number of KOG associated ESTs sequences and non-associated were 5193 and 2789, respectively.

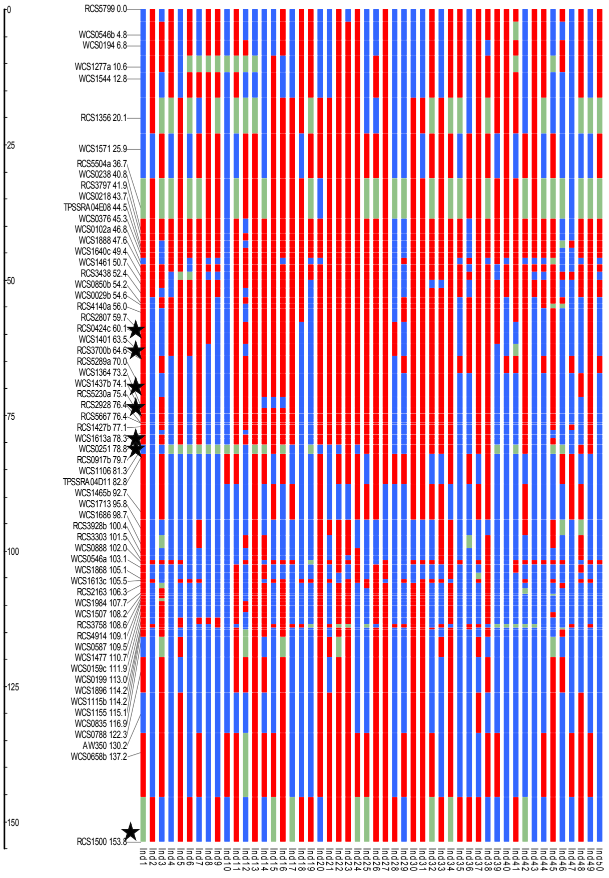
RCS5799 0.0
WCS0546b 4.8
WCS0194 6.8
WCS1277a 10.6
WCS1544 12.8
RCS1356 20.1
WCS1571 25.9
RCS5504a 36.7
WCS0238 40.8
RCS3797 41.9
WCS0218 43.7
TPSSRA04E08 44.5
WCS0376 45.3
WCS0102a 46.8
WCS1888 47.6
WCS1640c 49.4
WCS1461 50.7
RCS3438 52.4
WCS0850b 54.2
WCS0029b 54.6
RCS4140a 56.0
RCS2807 59.7
RCS0424c 60.1
WCS1401 63.5
RCS3700b 64.6
RCS5289a 70.0
WCS1364 73.2
WCS1437b 74.1
RCS5230a 75.4
RCS2928 76.4
RCS5667 76.4
RCS1427b 77.1
WCS1613a 78.3
WCS0251 78.8
RCS0917b 79.7
WCS1106 81.3
TPSSRA04D11 82.8
WCS1465b 92.7
WCS1713 95.8
WCS1686 98.7
RCS3928b 100.4
RCS3303 101.5
WCS0888 102.0
WCS0546a 103.1
WCS1868 105.1
WCS1613c 105.5
RCS2163 106.3
WCS1984 107.7
WCS1507 108.2
RCS3758 108.6
RCS4914 109.1
WCS0587 109.5
WCS1477 110.7
WCS0159c 111.9
WCS0199 113.0
WCS1896 114.2
WCS1115b 114.2
WCS1155 115.1
WCS0835 116.9
WCS0788 122.3
AW350 130.2
WCS0658b 137.2
RCS1500 153.8
0
25
50
75
100
125
150
cM

LG1a

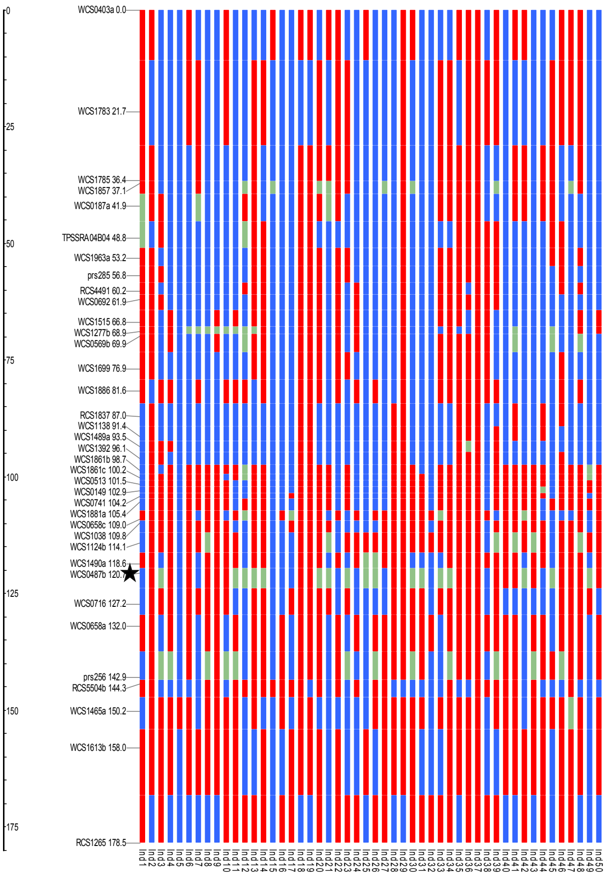
WCS0403a 0.0
WCS1783 21.7
WCS1785 36.4
WCS1857 37.1
WCS0187a 41.9
TPSSRA04B04 48.8
WCS1963a 53.2
prs285 56.8
RCS4491 60.2
WCS0692 61.9
WCS1515 66.8
WCS1277b 68.9
WCS0569b 69.9
WCS1699 76.9
WCS1886 81.6
RCS1837 87.0
WCS1138 91.4
WCS1489a 93.5
WCS1392 96.1
WCS1861b 98.7
WCS1861c 100.2
WCS0513 101.5
WCS0149 102.9
WCS0741 104.2
WCS1881a 105.4
WCS0658c 109.0
WCS1038 109.8
WCS1124b 114.1
WCS1490a 118.6
WCS0487b 120.7
WCS0716 127.2
WCS0658a 132.0
prs256 142.9
RCS5504b 144.3
WCS1465a 150.2
WCS1613b 158.0
RCS1265 178.5
0
25
50
75
100
125
150
175
cM

LG1b

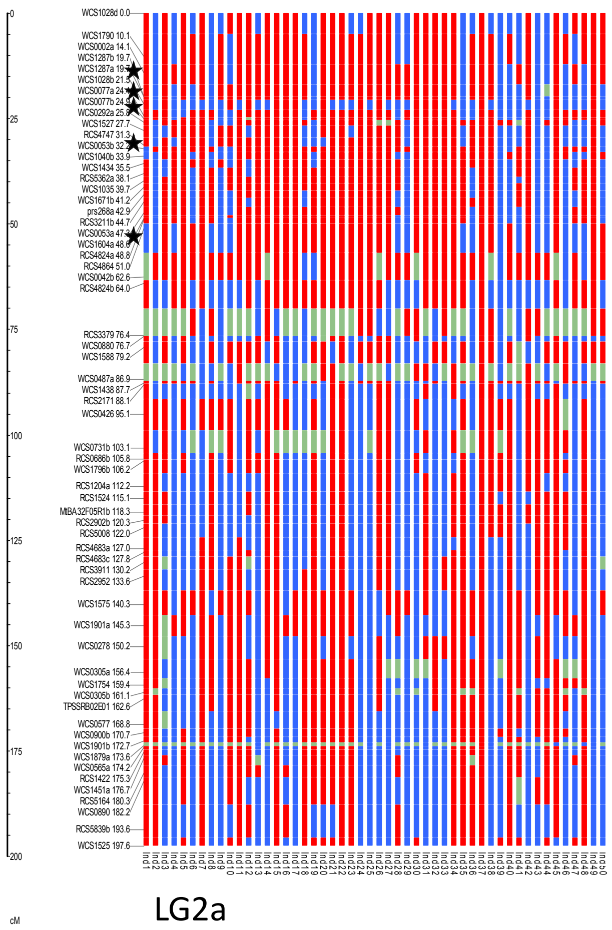

LG2a

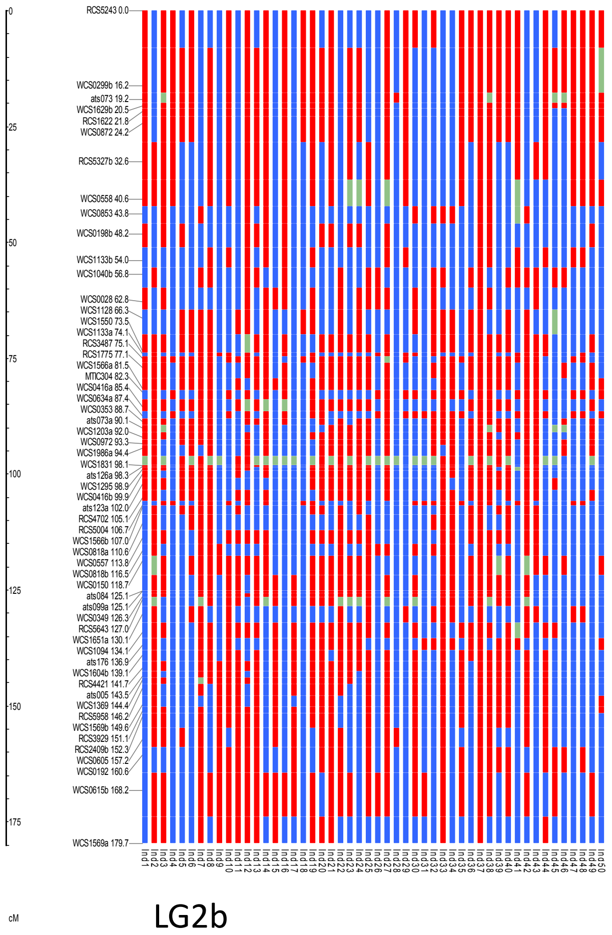

LG2b

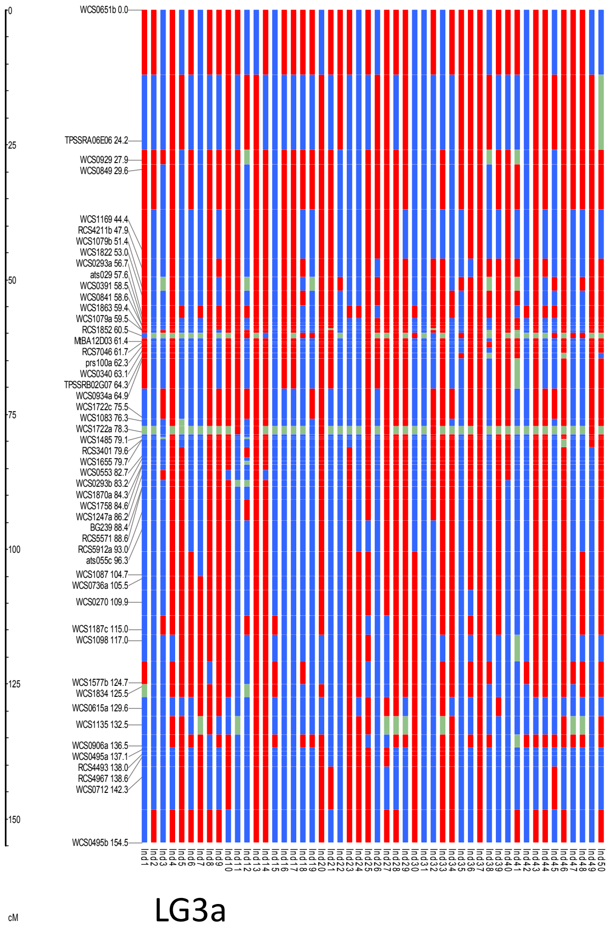
cM
LG3a
WCS0651b 0.0
TPSSRA06E06 24.2
WCS0929 27.9
WCS0849 29.6
WCS1169 44.4
RCS4211b 47.9
WCS1079b 51.4
WCS1822 53.0
WCS0293a 56.7
ats029 57.6
WCS0391 58.5
WCS0841 58.6
WCS1863 59.4
WCS1079a 59.5
RCS1852 60.5
MtBA12D03 61.4
RCS7046 61.7
prs100a 62.3
WCS0340 63.1
TPSSRB02G07 64.3
WCS0934a 64.9
WCS1722c 75.5
WCS1083 76.3
WCS1722a 78.3
WCS1485 79.1
RCS3401 79.6
WCS1655 79.7
WCS0553 82.7
WCS0293b 83.2
WCS1870a 84.3
WCS1758 84.6
WCS1247a 86.2
BG239 88.4
RCS5571 88.6
RCS5912a 93.0
ats055c 96.3
WCS1087 104.7
WCS0736a 105.5
WCS0270 109.9
WCS1187c 115.0
WCS1098 117.0
WCS1577b 124.7
WCS1834 125.5
WCS0615a 129.6
WCS1135 132.5
WCS0906a 136.5
WCS0495a 137.1
RCS4493 138.0
RCS4967 138.6
WCS0712 142.3
WCS0495b 154.5

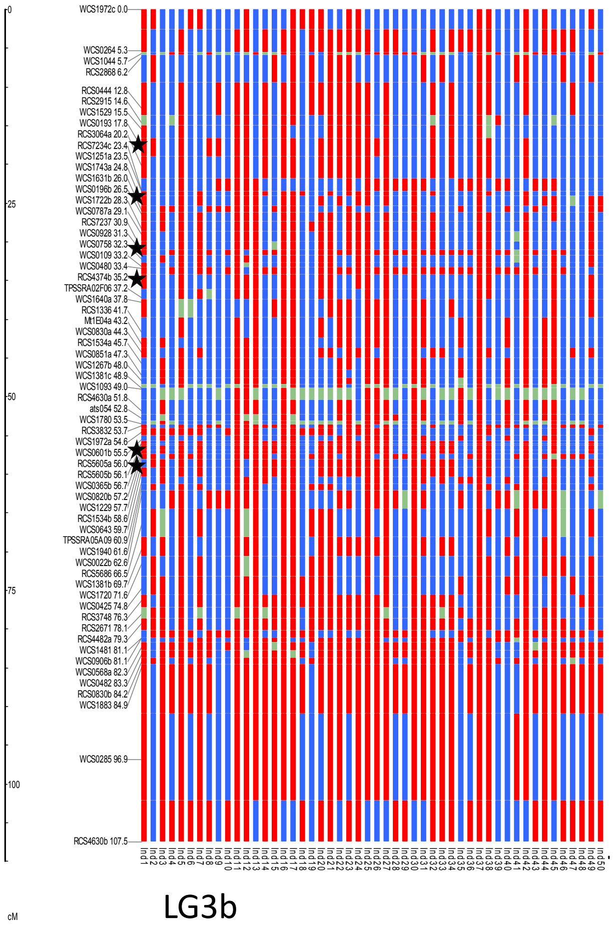
cM
LG3b
WCS1972c 0.0
WCS0264 5.3
WCS1044 5.7
RCS2868 6.2
RCS0444 12.8
RCS2915 14.6
WCS1529 15.5
WCS0193 17.8
RCS3064a 20.2
RCS7234c 23.4
WCS1251a 23.5
WCS1743a 24.8
WCS1631b 26.0
WCS0196b 26.5
WCS1722b 28.3
WCS0787a 29.1
RCS7237 30.9
WCS0928 31.3
WCS0758 32.3
WCS0109 33.2
WCS0480 33.4
RCS4374b 35.2
TPSSRA02F06 37.2
WCS1640a 37.8
RCS1336 41.7
Mt1E04a 43.2
WCS0830a 44.3
RCS1534a 45.7
WCS0851a 47.3
WCS1267b 48.0
WCS1381c 48.9
WCS1093 49.0
RCS4630a 51.8
ats054 52.8
WCS1780 53.5
RCS3832 53.7
WCS1972a 54.6
WCS0601b 55.5
RCS5605a 56.0
RCS5605b 56.1
WCS0365b 56.7
WCS0820b 57.2
WCS1229 57.7
RCS1534b 58.6
WCS0643 59.7
TPSSRA05A09 60.9
WCS1940 61.6
WCS0022b 62.6
RCS5686 66.5
WCS1381b 69.7
WCS1720 71.6
WCS0425 74.8
RCS3748 76.3
RCS2671 78.1
RCS4482a 79.3
WCS1481 81.1
WCS0906b 81.1
WCS0568a 82.3
WCS0482 83.3
RCS0830b 84.2
WCS1883 84.9
WCS0285 96.9
RCS4630b 107.5

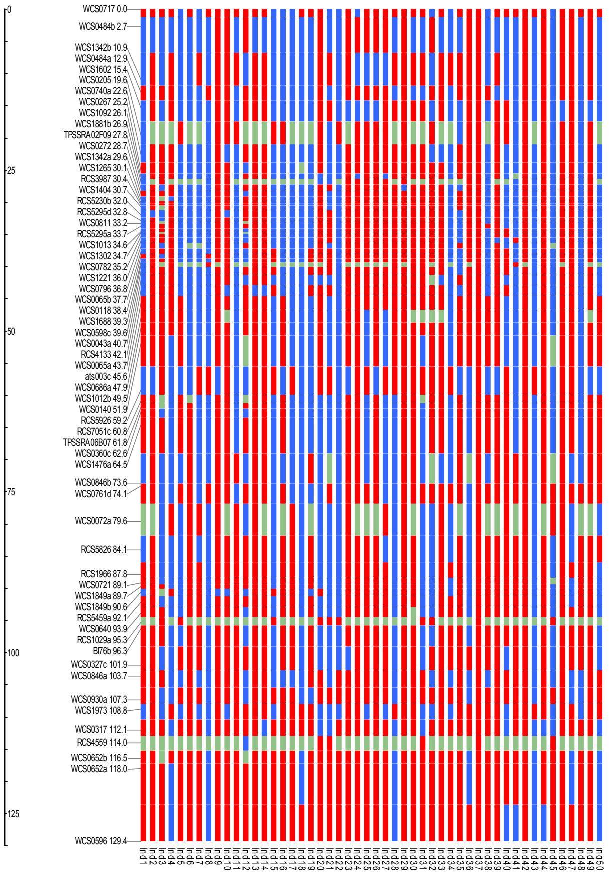
WCS0717 0.0
WCS0484b 2.7
WCS1342b 10.9
WCS0484a 12.9
WCS1602 15.4
WCS0205 19.6
WCS0740a 22.6
WCS0267 25.2
WCS1092 26.1
WCS1881b 26.9
TPSSRA02F09 27.8
WCS0272 28.7
WCS1342a 29.6
WCS1265 30.1
RCS3987 30.4
WCS1404 30.7
RCS5230b 32.0
RCS5295d 32.8
WCS0811 33.2
RCS5295a 33.7
WCS1013 34.6
WCS1302 34.7
WCS0782 35.2
WCS1221 36.0
WCS0796 36.8
WCS0065b 37.7
WCS0118 38.4
WCS1688 39.3
WCS0598c 39.6
WCS0043a 40.7
RCS4133 42.1
WCS0065a 43.7
ats003c 45.6
WCS0686a 47.9
WCS1012b 49.5
WCS0140 51.9
RCS5926 59.2
RCS7051c 60.8
TPSSRA06B07 61.8
WCS0360c 62.6
WCS1476a 64.5
WCS0846b 73.6
WCS0761d 74.1
WCS0072a 79.6
RCS5826 84.1
RCS1966 87.8
WCS0721 89.1
WCS1849a 89.7
WCS1849b 90.6
RCS5459a 92.1
WCS0640 93.9
RCS1029a 95.3
BI76b 96.3
WCS0327c 101.9
WCS0846a 103.7
WCS0930a 107.3
WCS1973 108.8
WCS0317 112.1
RCS4559 114.0
WCS0652b 116.5
WCS0652a 118.0
WCS0596 129.4
0
25
50
75
100
125
cM

LG4a

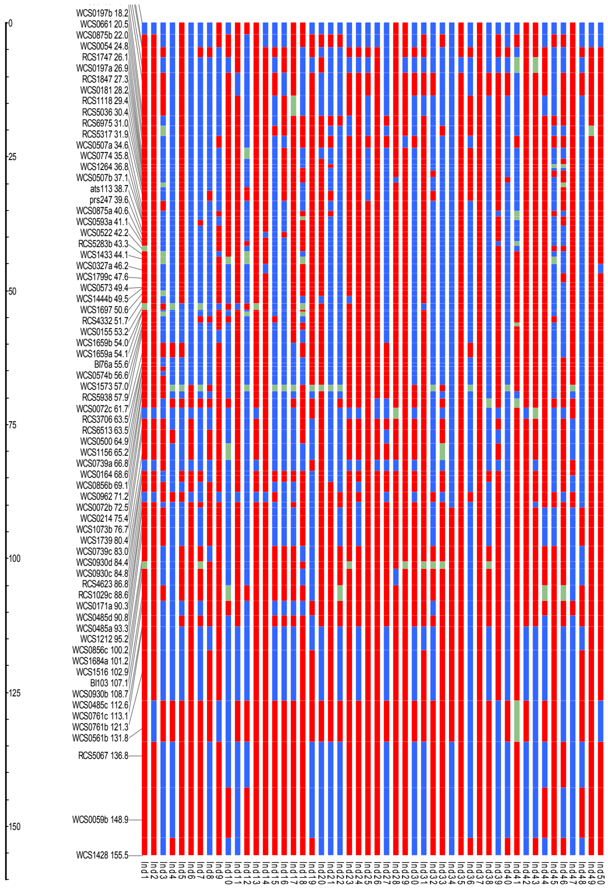
WCS0197b 18.2
WCS0661 20.5
WCS0875b 22.0
WCS0054 24.8
RCS1747 26.1
WCS0197a 26.9
RCS1847 27.3
WCS0181 28.2
RCS1118 29.4
RCS5036 30.4
RCS6975 31.0
RCS5317 31.9
WCS0507a 34.6
WCS0774 35.8
WCS1264 36.8
WCS0507b 37.1
ats113 38.7
prs247 39.6
WCS0875a 40.6
WCS0593a 41.1
WCS0522 42.2
RCS5283b 43.3
WCS1433 44.1
WCS0327a 46.2
WCS1799c 47.6
WCS0573 49.4
WCS1444b 49.5
WCS1697 50.6
RCS4332 51.7
WCS0155 53.2
WCS1659b 54.0
WCS1659a 54.1
BI76a 55.6
WCS0574b 56.6
WCS1573 57.0
RCS5938 57.9
WCS0072c 61.7
RCS3706 63.5
RCS6513 63.5
WCS0500 64.9
WCS1156 65.2
WCS0739a 66.8
WCS0164 68.6
WCS0856b 69.1
WCS0962 71.2
WCS0072b 72.5
WCS0214 75.4
WCS1073b 76.7
WCS1739 80.4
WCS0739c 83.0
WCS0930d 84.4
WCS0930c 84.8
RCS4623 86.8
RCS1029c 88.6
WCS0171a 90.3
WCS0485d 90.8
WCS0485a 93.3
WCS1212 95.2
WCS0856c 100.2
WCS1684a 101.2
WCS1516 102.9
BI103 107.1
WCS0930b 108.7
WCS0485c 112.6
WCS0761c 113.1
WCS0761b 121.3
WCS0561b 131.8
RCS5067 136.8
WCS0059b 148.9
WCS1428 155.5
0
25
50
75
100
125
150
cM

LG4b

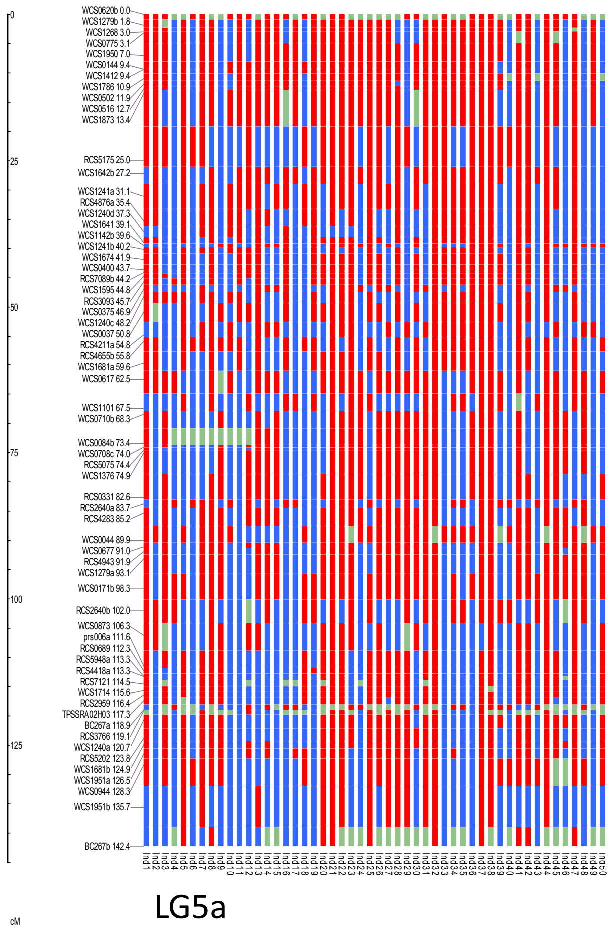
LG5a

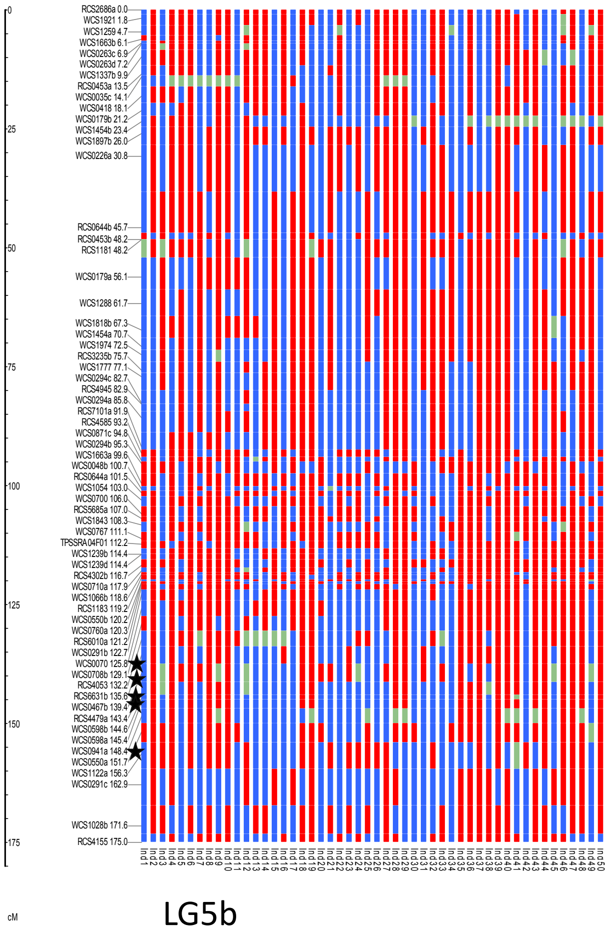
LG5b

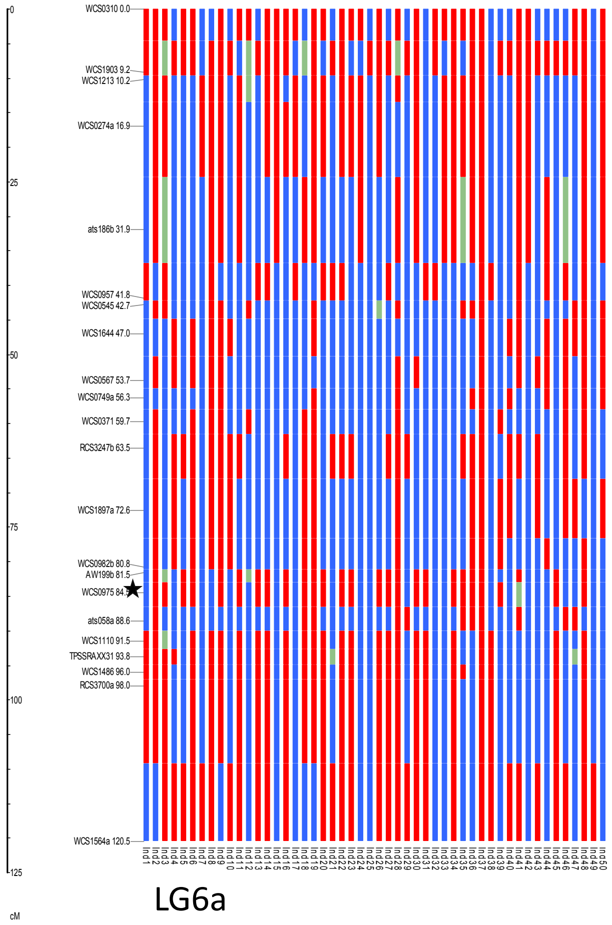
WCS0310 0.0
WCS1903 9.2
WCS1213 10.2
WCS0274a 16.9
ats186b 31.9
WCS0957 41.8
WCS0545 42.7
WCS1644 47.0
WCS0567 53.7
WCS0749a 56.3
WCS0371 59.7
RCS3247b 63.5
WCS1897a 72.6
WCS0982b 80.8
AW199b 81.5
WCS0975 84.7
ats058a 88.6
WCS1110 91.5
TPSSRAXX31 93.8
WCS1486 96.0
RCS3700a 98.0
WCS1564a 120.5
cM

LG6a

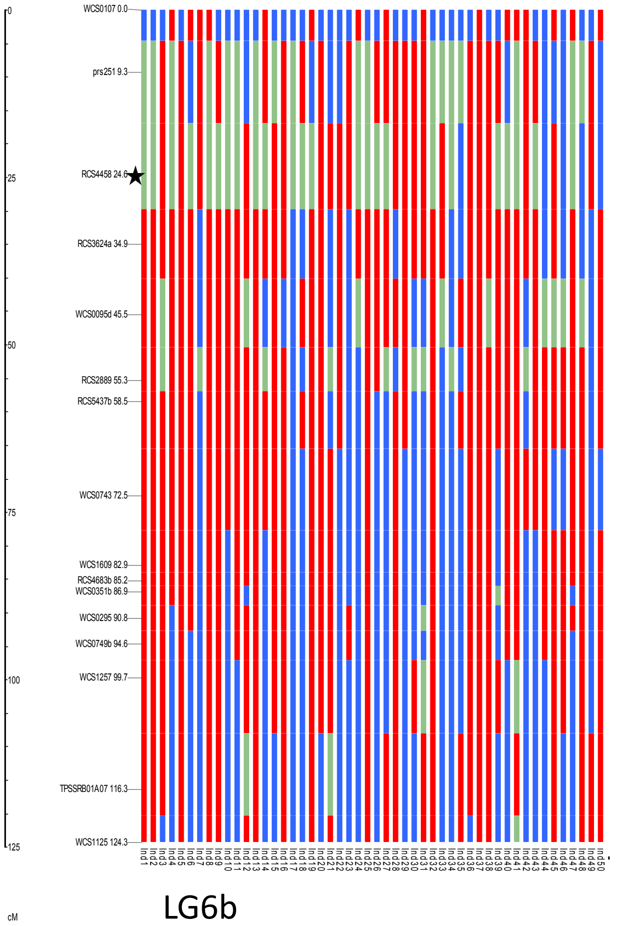
WCS0107 0.0
prs251 9.3
RCS4458 24.6
RCS3624a 34.9
WCS0095d 45.5
RCS2889 55.3
RCS5437b 58.5
WCS0743 72.5
WCS1609 82.9
RCS4683b 85.2
WCS0351b 86.9
WCS0295 90.8
WCS0749b 94.6
WCS1257 99.7
TPSSRB01A07 116.3
WCS1125 124.3
cM

LG6b

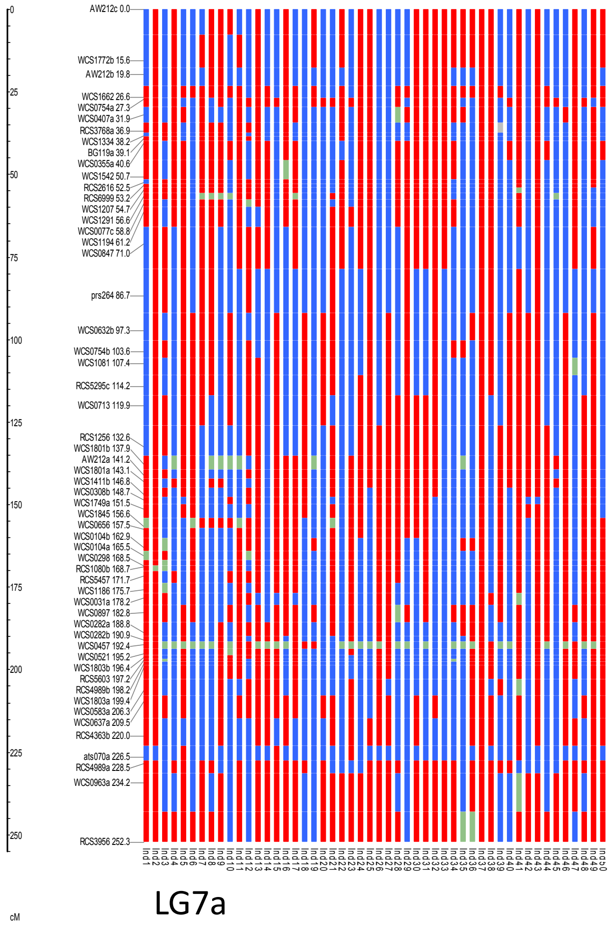
AW212c 0.0
WCS1772b 15.6
AW212b 19.8
WCS1662 26.6
WCS0754a 27.3
WCS0407a 31.9
RCS3768a 36.9
WCS1334 38.2
BG119a 39.1
WCS0355a 40.6
WCS1542 50.7
RCS2616 52.5
RCS6999 53.2
WCS1207 54.7
WCS1291 56.6
WCS0077c 58.8
WCS1194 61.2
WCS0847 71.0
prs264 86.7
WCS0632b 97.3
WCS0754b 103.6
WCS1081 107.4
RCS5295c 114.2
WCS0713 119.9
RCS1256 132.6
WCS1801b 137.9
AW212a 141.2
WCS1801a 143.1
WCS1411b 146.8
WCS0308b 148.7
WCS1749a 151.5
WCS1845 156.6
WCS0656 157.5
WCS0104b 162.9
WCS0104a 165.5
WCS0298 168.5
RCS1080b 168.7
RCS5457 171.7
WCS1186 175.7
WCS0031a 178.2
WCS0897 182.8
WCS0282a 188.8
WCS0282b 190.9
WCS0457 192.4
WCS0521 195.2
WCS1803b 196.4
RCS5603 197.2
RCS4989b 198.2
WCS1803a 199.4
WCS0583a 206.3
WCS0637a 209.5
RCS4363b 220.0
ats070a 226.5
RCS4989a 228.5
WCS0963a 234.2
RCS3956 252.3
0
25
50
75
100
125
150
175
200
225
250
cM

LG7a

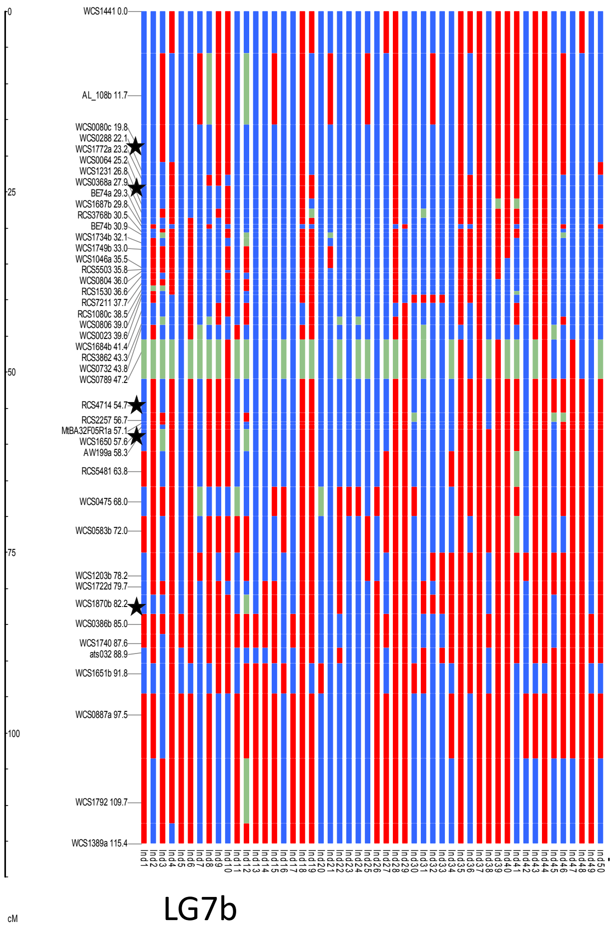
WCS1441 0.0
AL_108b 11.7
WCS0080c 19.8
WCS0288 22.1
WCS1772a 23.2
WCS0064 25.2
WCS1231 26.8
WCS0368a 27.9
BE74a 29.3
WCS1687b 29.8
RCS3768b 30.5
BE74b 30.9
WCS1734b 32.1
WCS1749b 33.0
WCS1046a 35.5
RCS5503 35.8
WCS0804 36.0
RCS1530 36.6
RCS7211 37.7
RCS1080c 38.5
WCS0806 39.0
WCS0023 39.6
WCS1684b 41.4
RCS3862 43.3
WCS0732 43.8
WCS0789 47.2
RCS4714 54.7
RCS2257 56.7
MtBA32F05R1a 57.1
WCS1650 57.6
AW199a 58.3
RCS5481 63.8
WCS0475 68.0
WCS0583b 72.0
WCS1203b 78.2
WCS1722d 79.7
WCS1870b 82.2
WCS0386b 85.0
WCS1740 87.6
ats032 88.9
WCS1651b 91.8
WCS0887a 97.5
WCS1792 109.7
WCS1389a 115.4
0
25
50
75
100
cM

LG7b

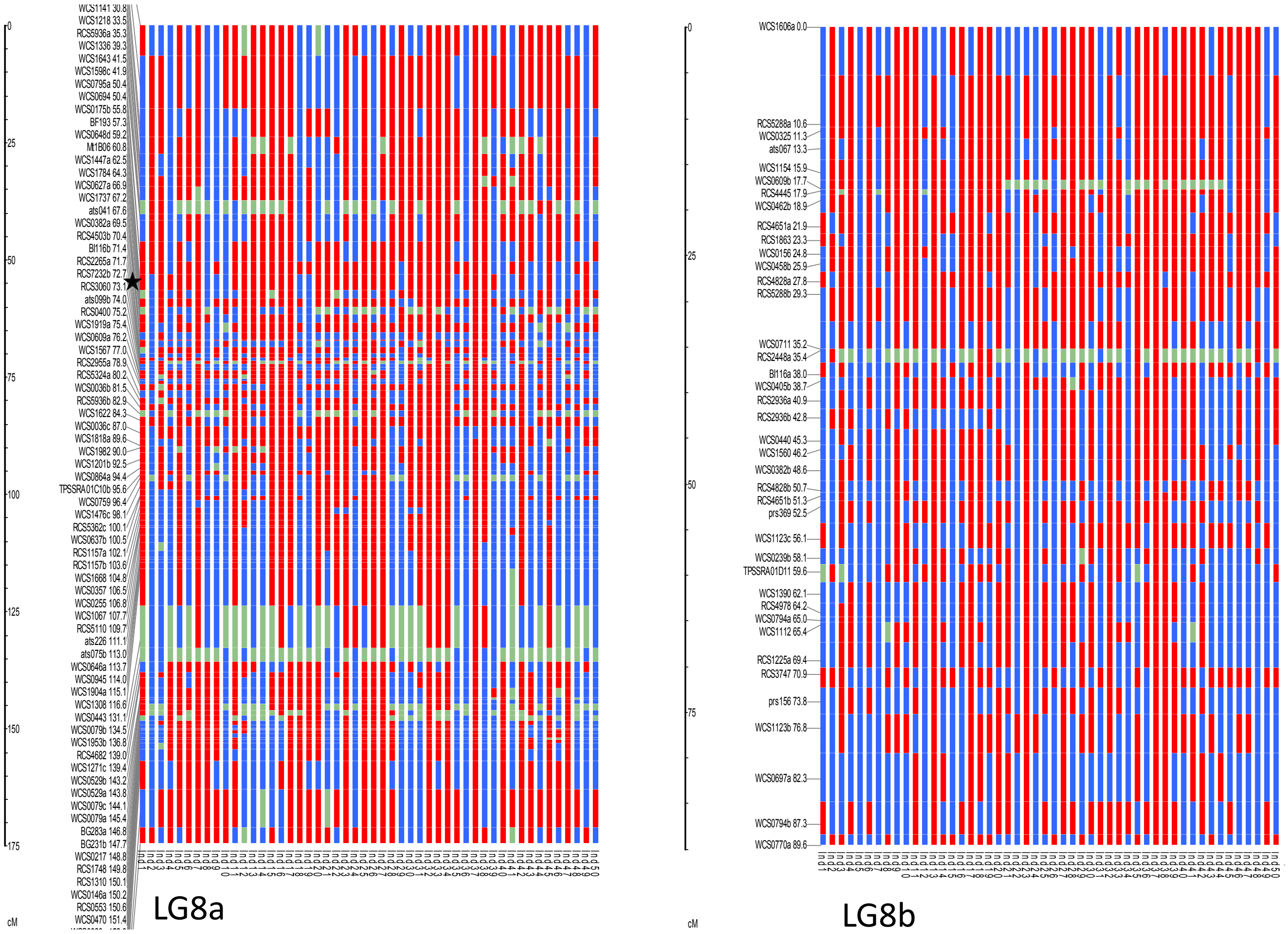

Figure S3 Graphical genotypes of 50 of the 188 F_1 populations mapped onto the largest linkage group shown in Figure S1. The red and blue colors show the two haplotypes of 'T17-349'. The light green color shows missing data. Black stars indicate loci showing a high LD (r^2>0.5) between loci mapped onto different linkage groups.

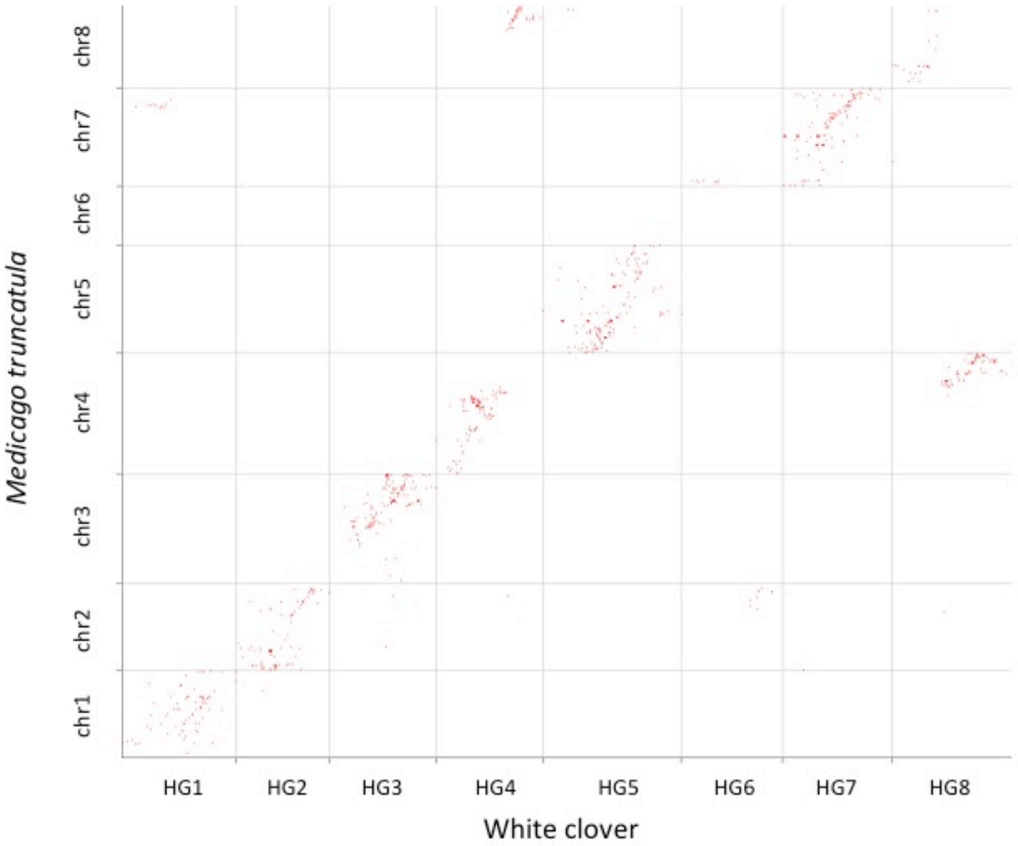

Figure S4 Graphical view of syntenic blocks between white clover and *M. truncatula*. X and Y axis show position on mapped markers on an white clover linkage map and chromosomes of *M. truncatula* genome, respectively.

List of loci identified LD showing r2>0.5 between loci mapped onto different linkage groups.

Mapped LG	Locus name	Mapped LG	Locus name
1a	RCS1500	3b	WCS0643
1a	RCS2807	3b	WCS1743a
1a	RCS5289a	5b	RCS6631b
1a	WCS0251	5b	WCS0467b
1a	WCS1106	5b	WCS0598a
1a	WCS1364	5b	WCS0598b
1a	WCS1401	5b	WCS1122a
1b	WCS0487b	6a	WCS0975
2a	RCS4747	6b	RCS4458
2a	RCS4864	7b	ats032
2a	WCS0002a	7b	AW199a
2a	WCS0077a	7b	RCS4714
2a	WCS1287b	7b	WCS0064
3b	RCS3064a	7b	WCS0080c
3b	RCS4374b	7b	WCS1870b
3b	RCS5605a	8a	WCS0175b
3b	RCS7237		

RCS1500

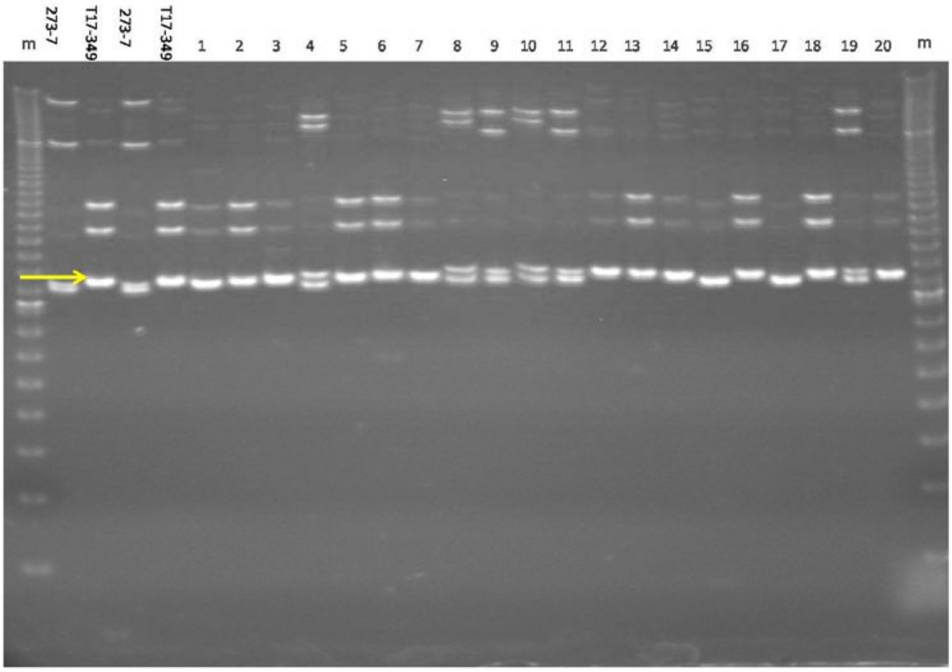

RCS2807

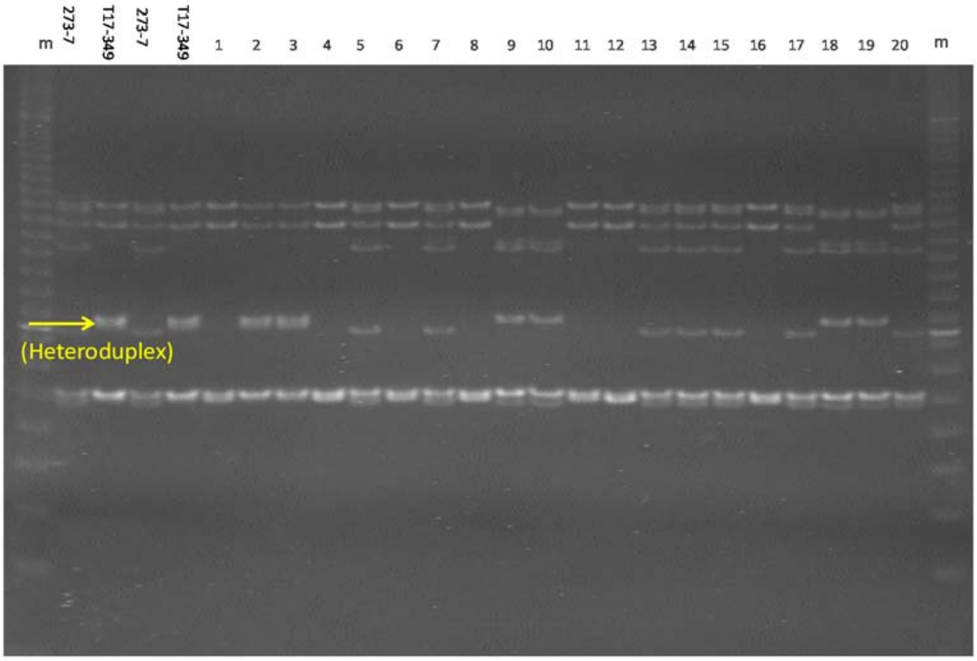

RCS5289a

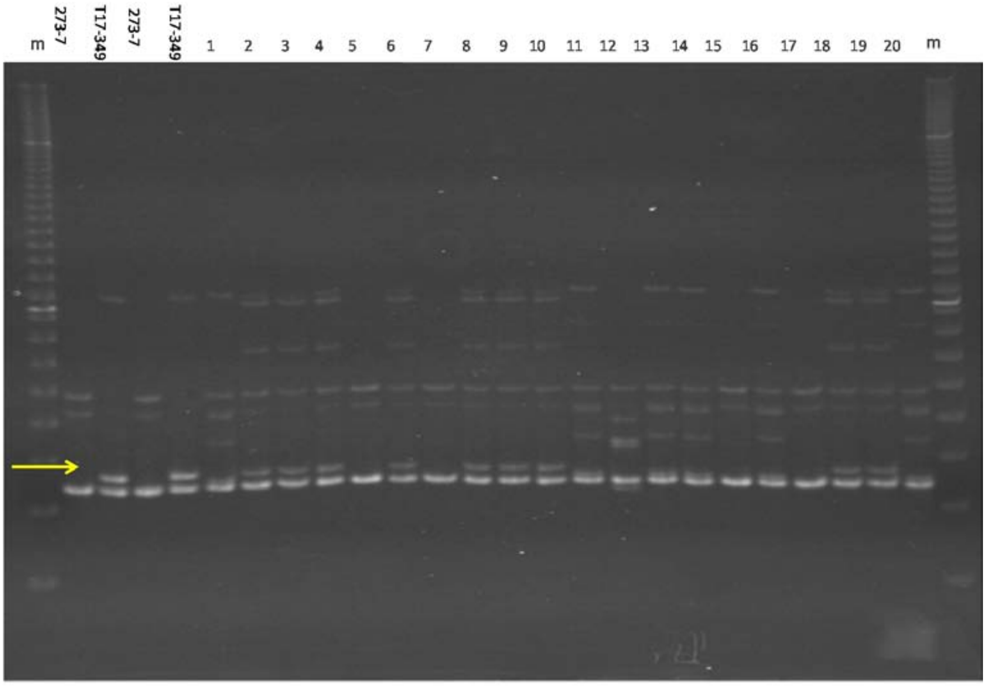

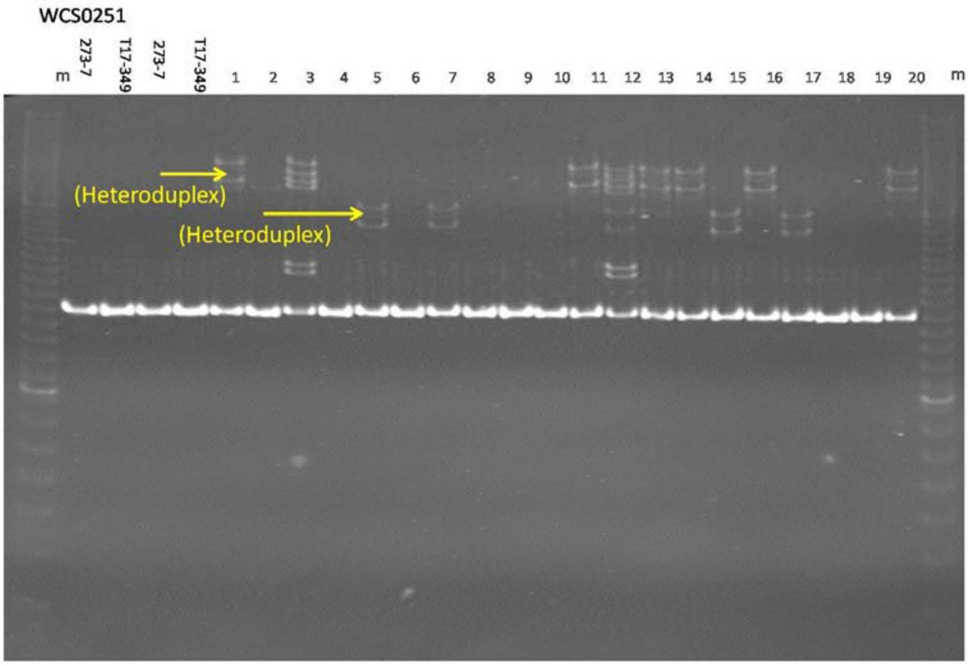
WCS0251
m 273-7 T17-349 273-7 T17-349 1 2 3 4 5 6 7 8 9 10 11 12 13 14 15 16 17 18 19 20 m
(Heteroduplex)
(Heteroduplex)

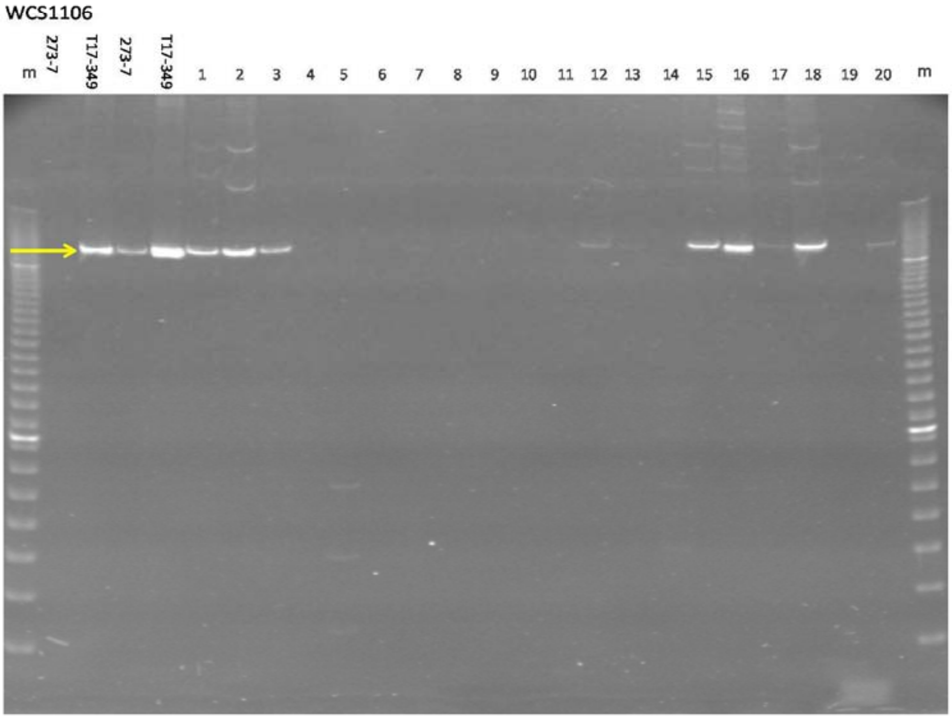
WCS1106
m 273-7 T17-349 273-7 T17-349 1 2 3 4 5 6 7 8 9 10 11 12 13 14 15 16 17 18 19 20 m

WCS1364

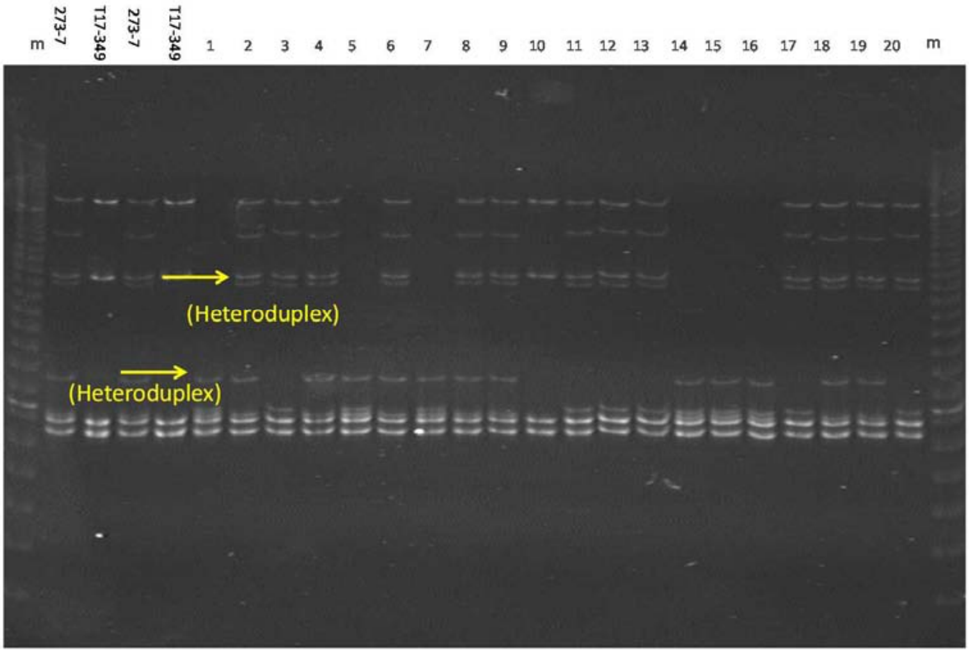

WCS1401

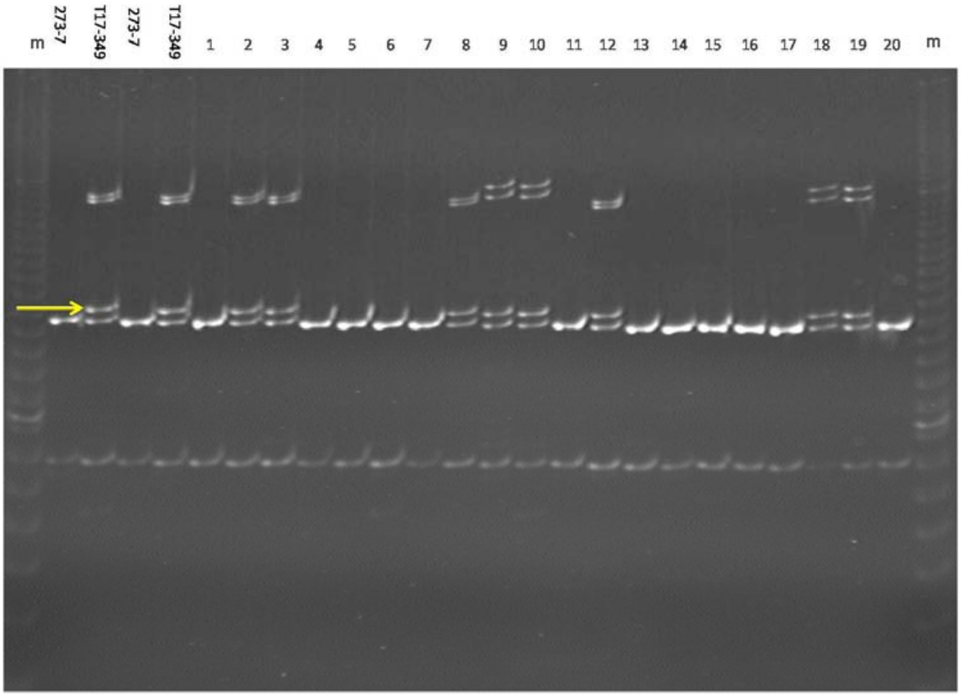

WCS0487b

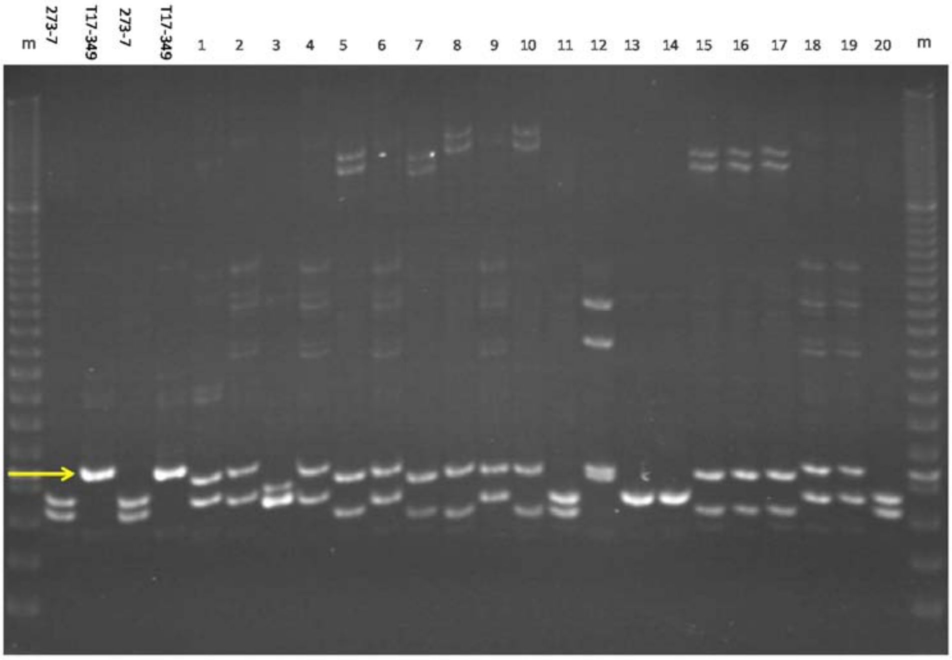

RCS4747

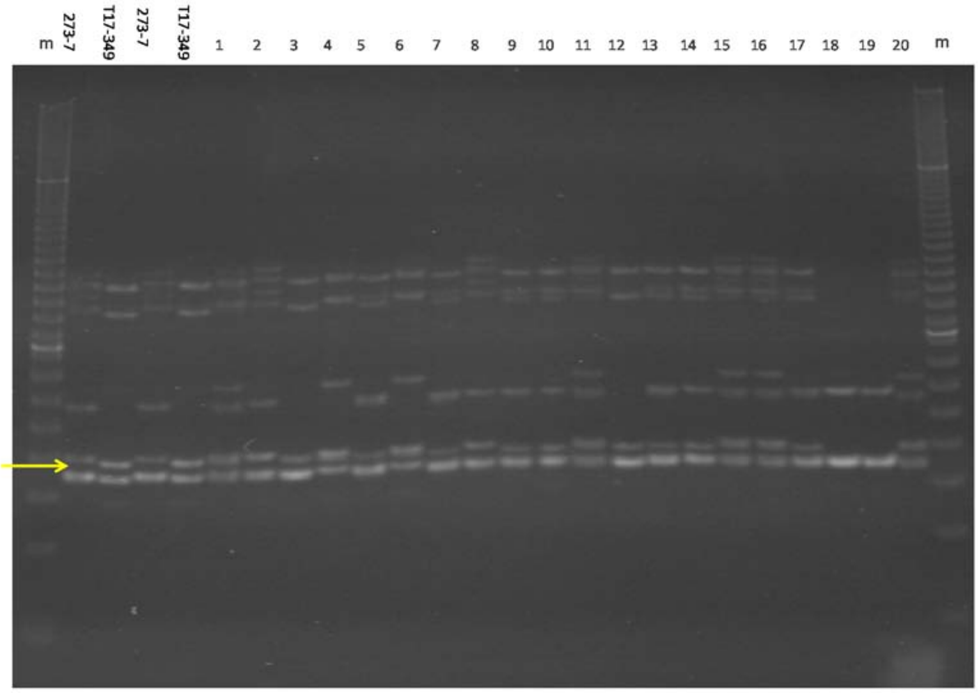

RCS4864

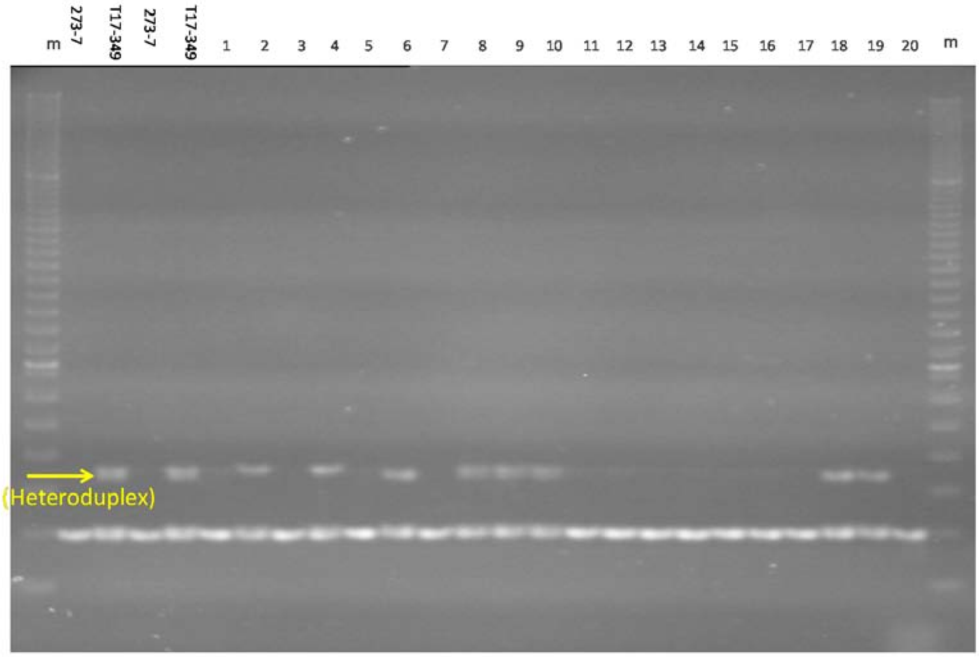

WCS0002a

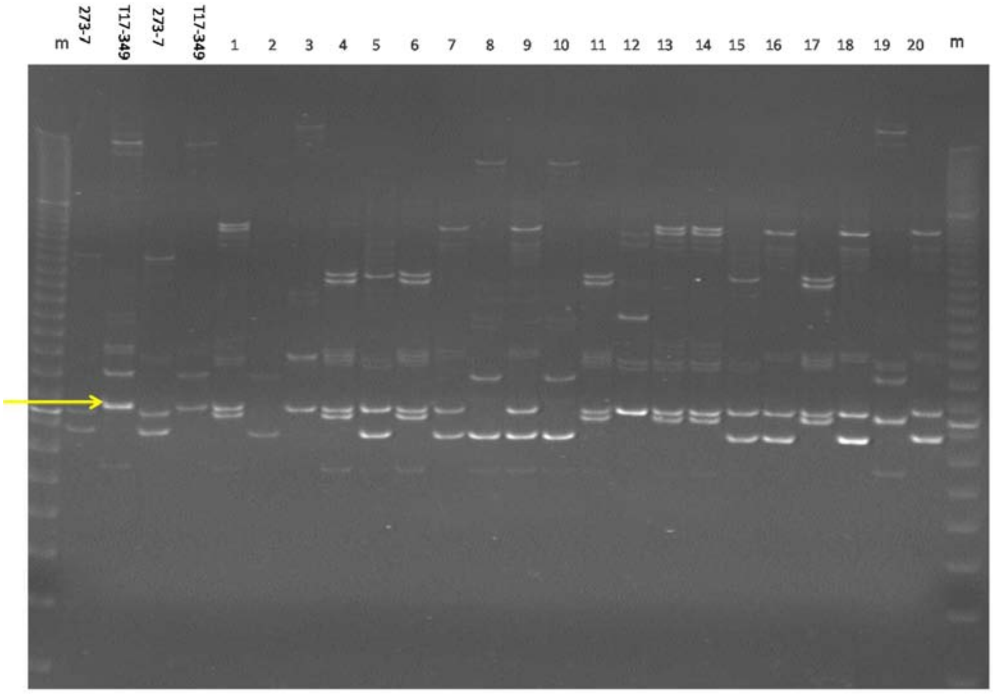

WCS00077a

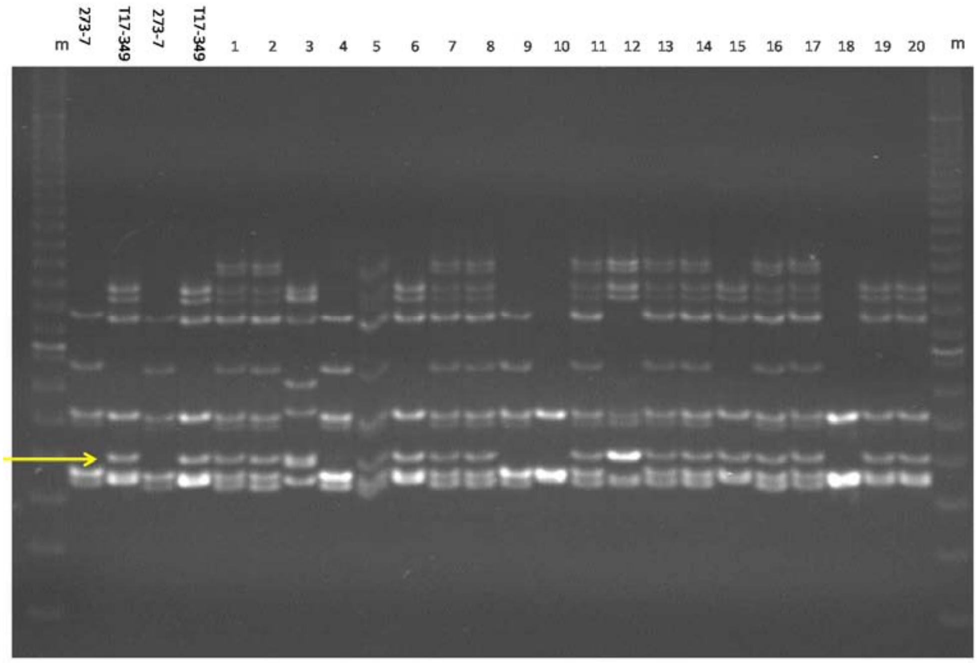

WCS1287b

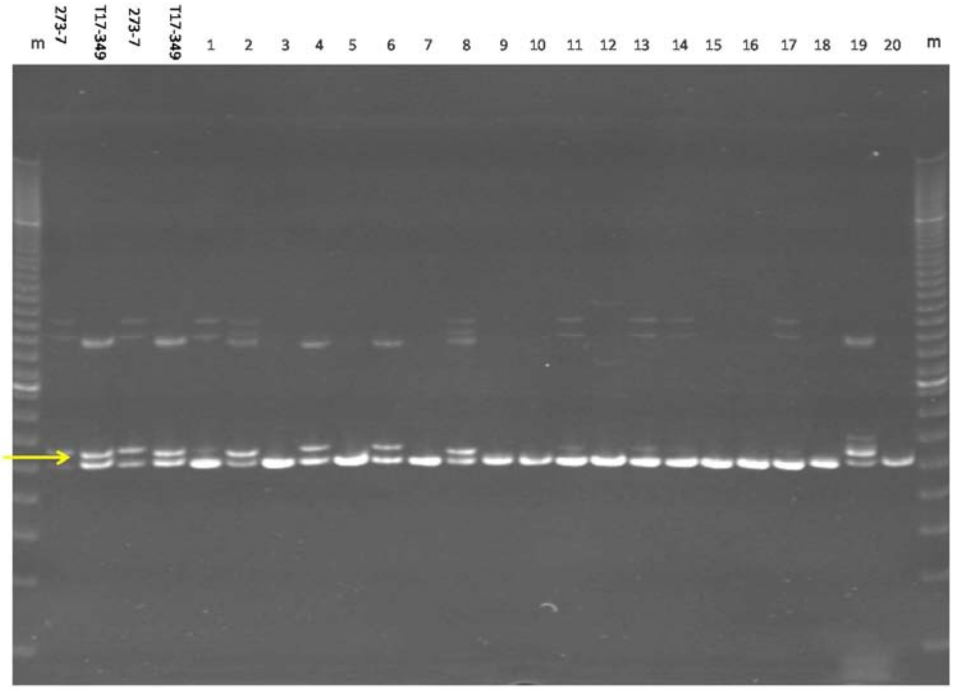

RCS3064a

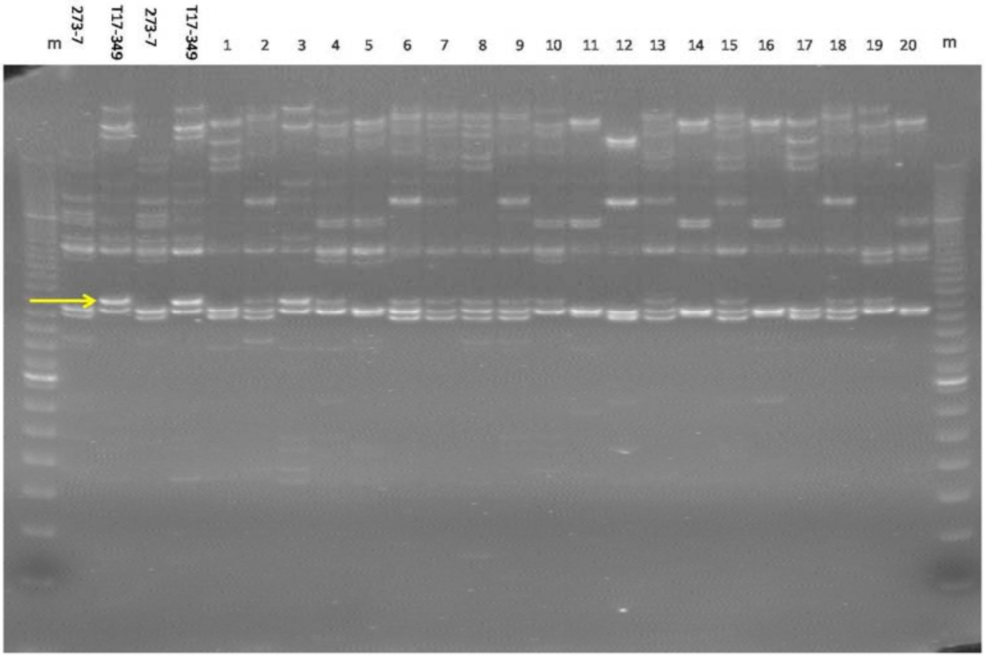

RCS4374b

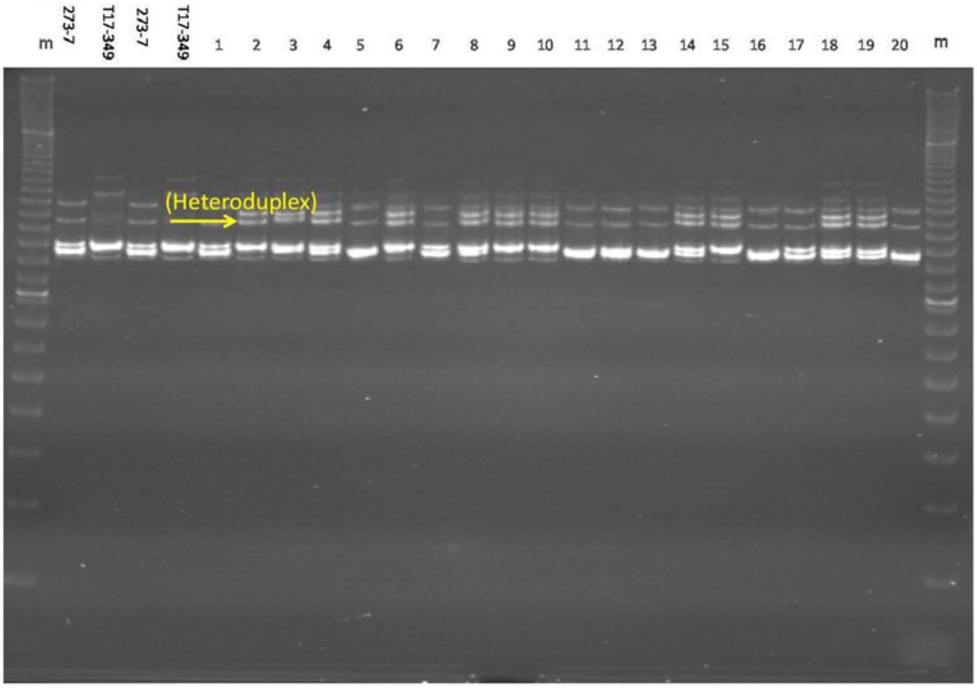

RCS5605a

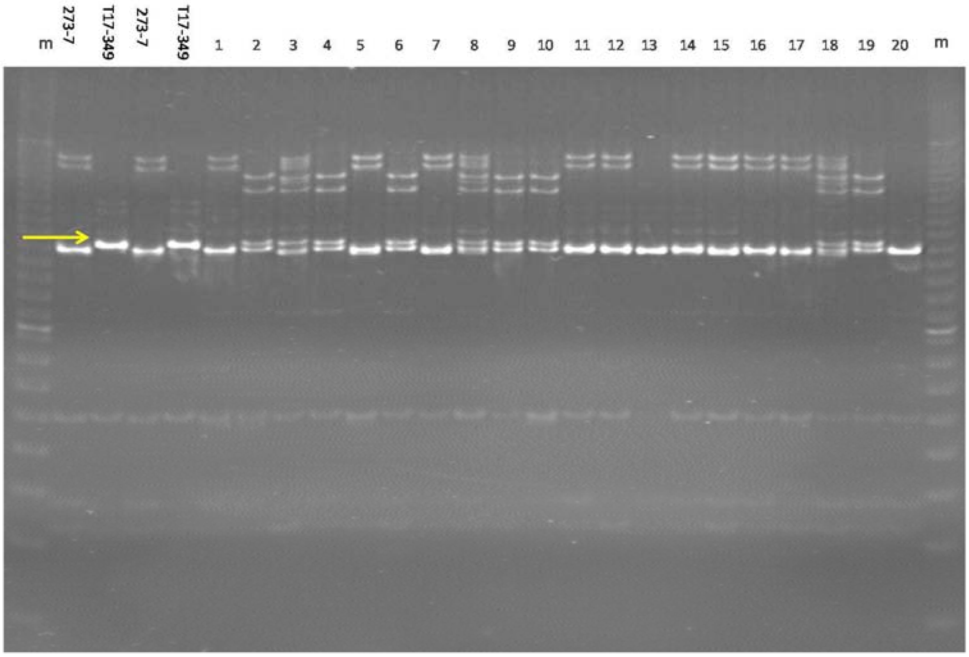

RCS7237

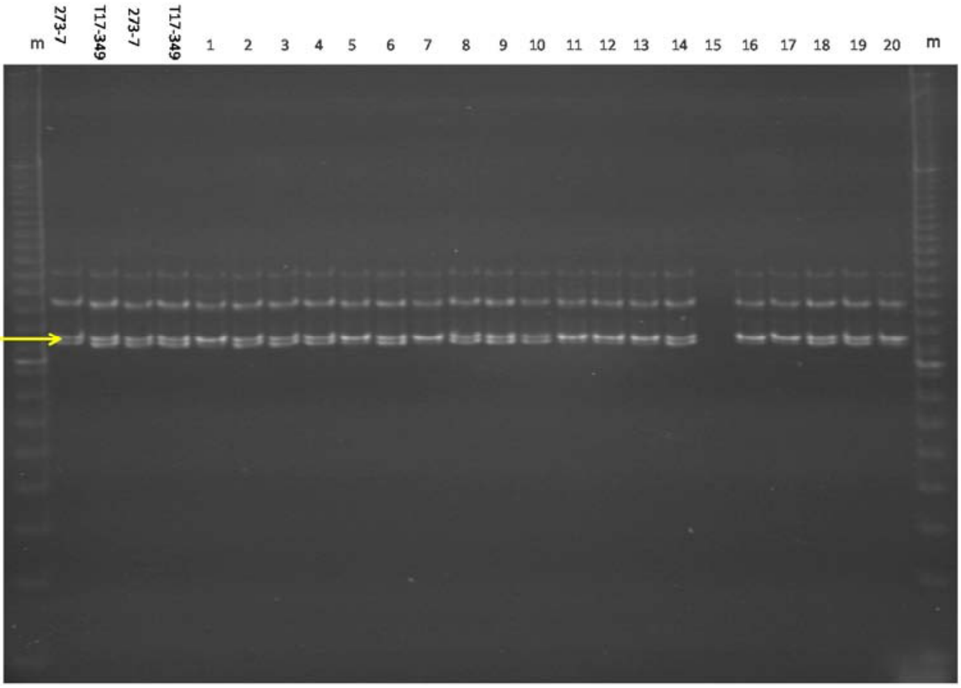

WCS0643

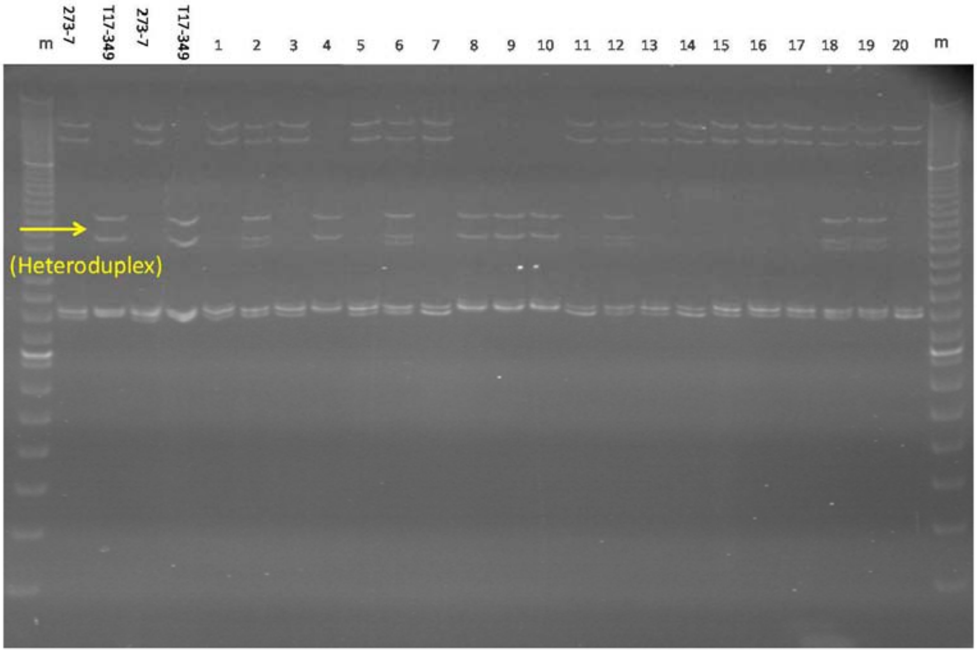

WCS1743a

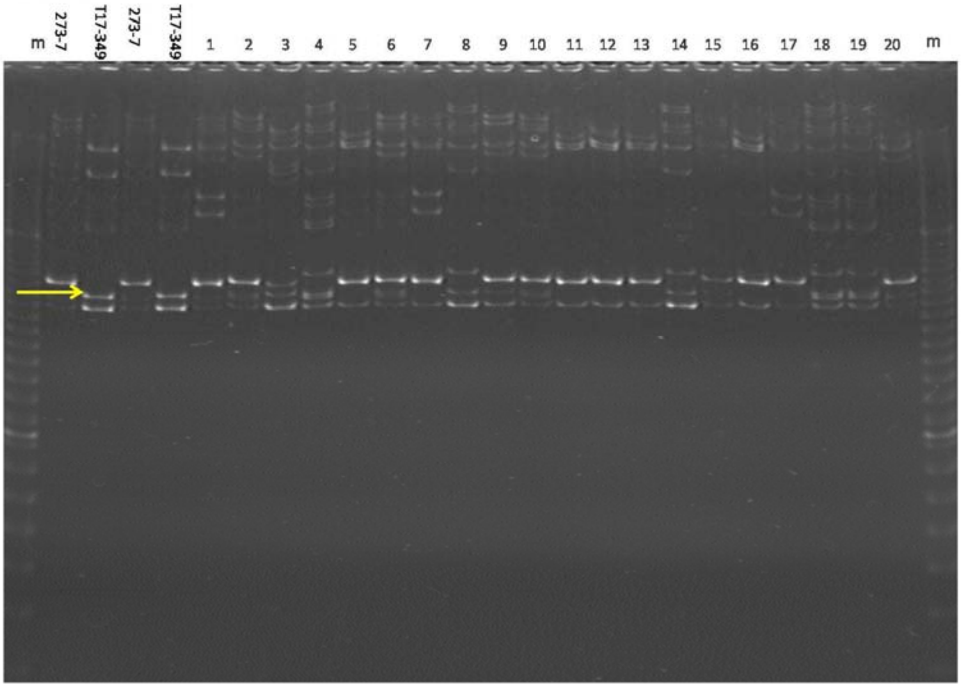

RCS6631b

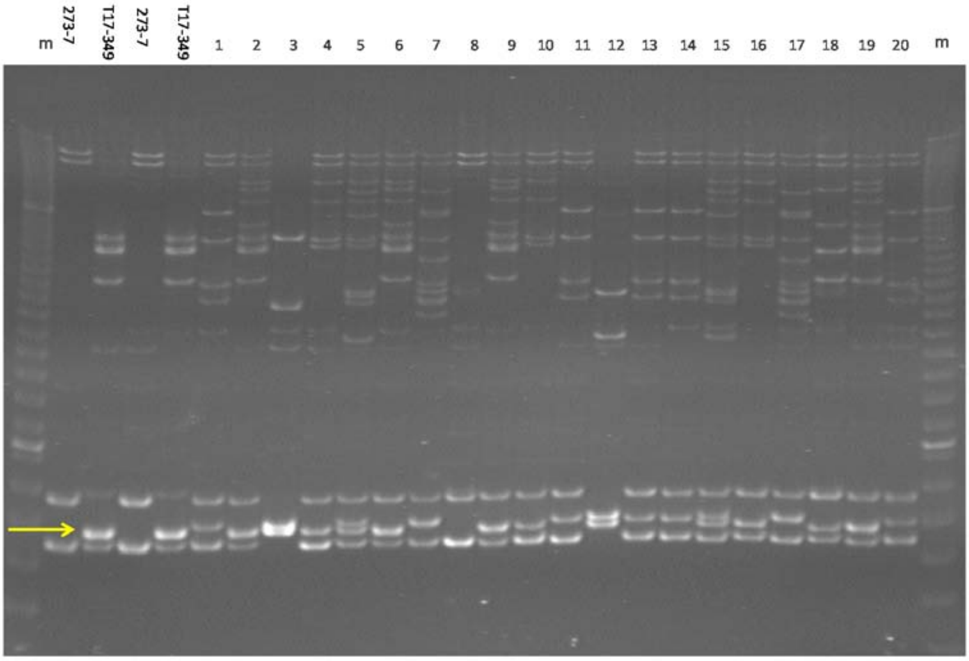

WCS0467b

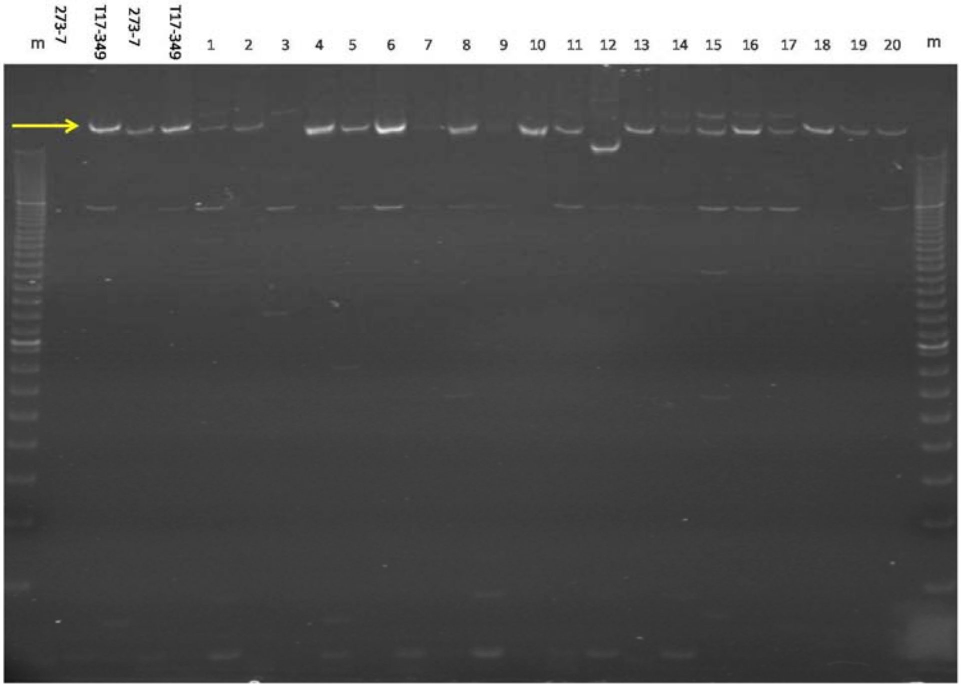

WCS0598a and WCS0598b

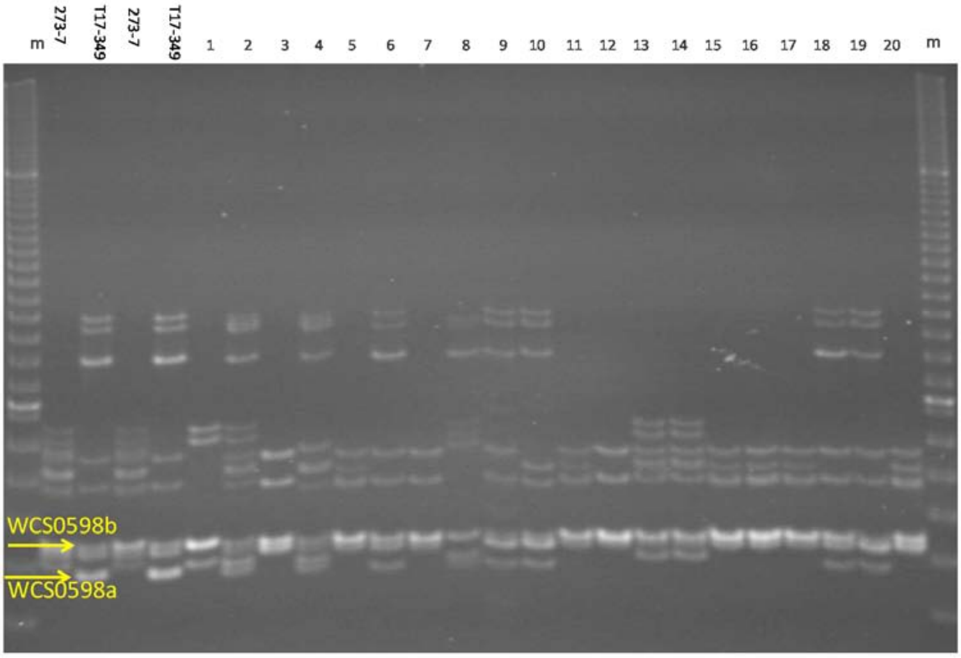

WCS1122a

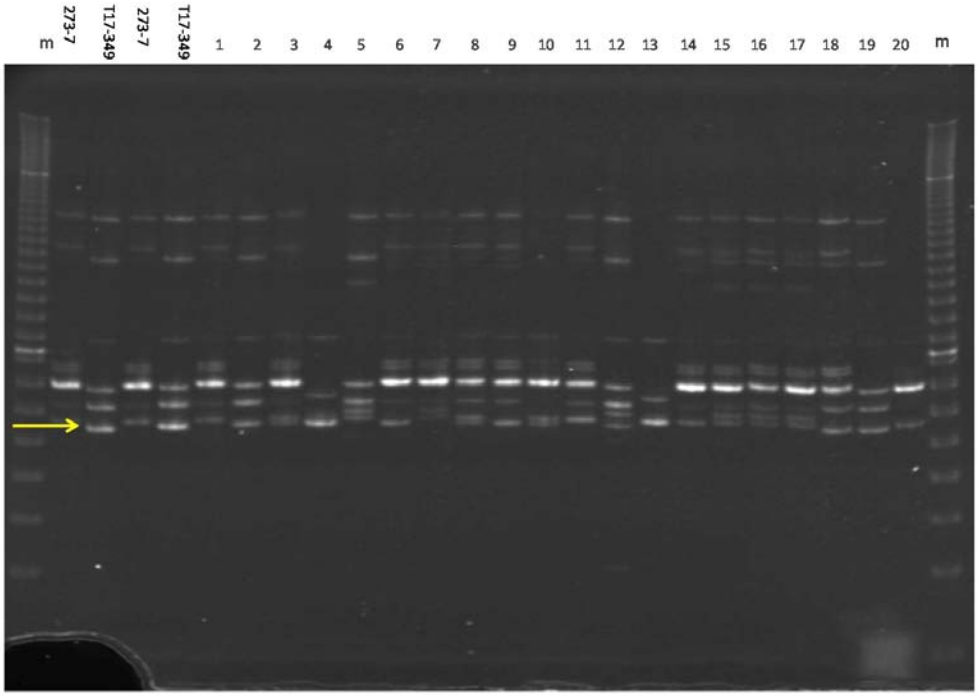

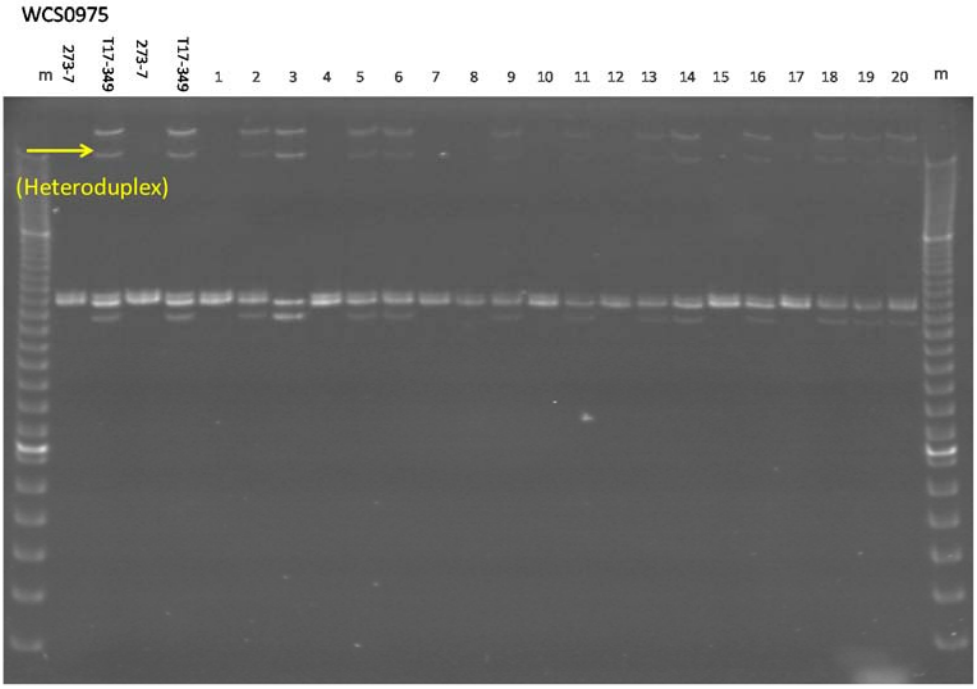
WCS0975
m
273-7
T17-349
273-7
T17-349
1 2 3 4 5 6 7 8 9 10 11 12 13 14 15 16 17 18 19 20
m
(Heteroduplex)

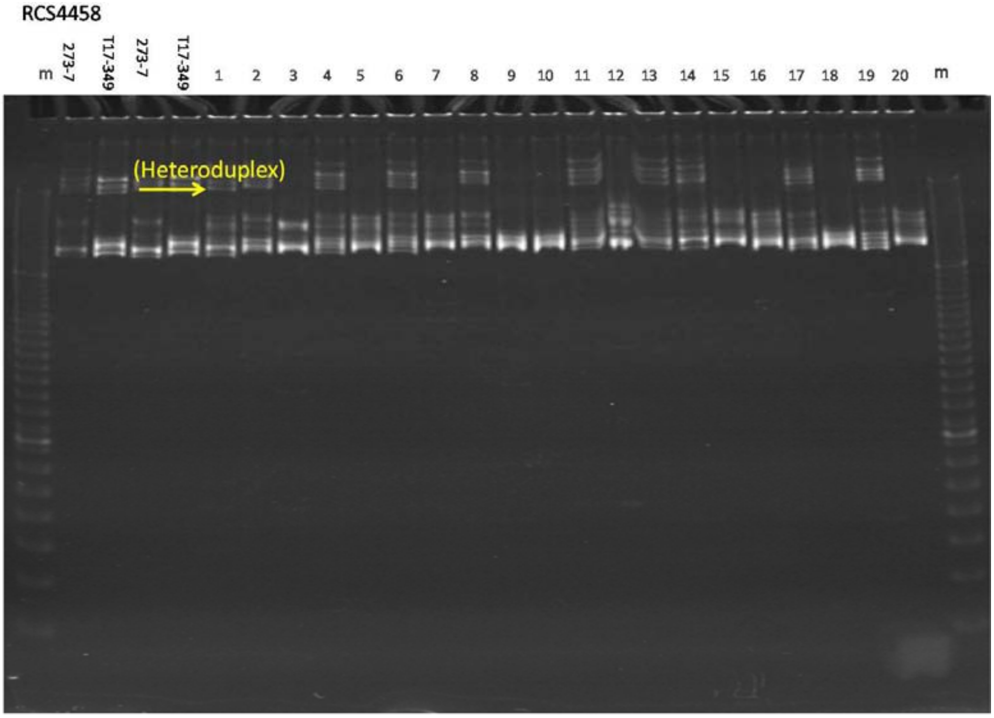
RCS4458
m
273-7
T17-349
273-7
T17-349
1 2 3 4 5 6 7 8 9 10 11 12 13 14 15 16 17 18 19 20
m
(Heteroduplex)

ats032

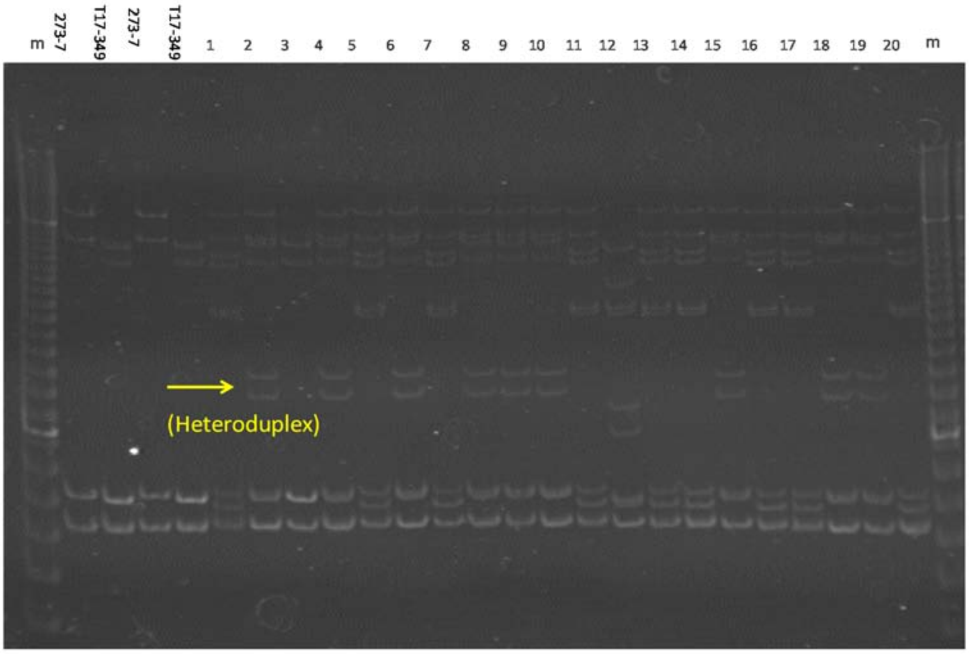

AW199a

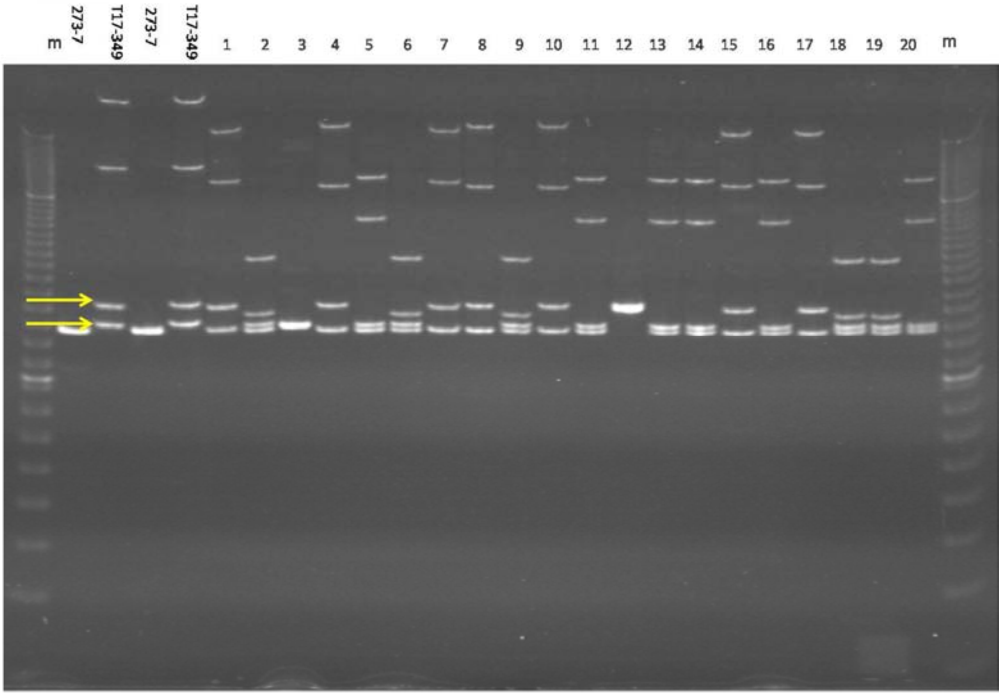

RCS4714

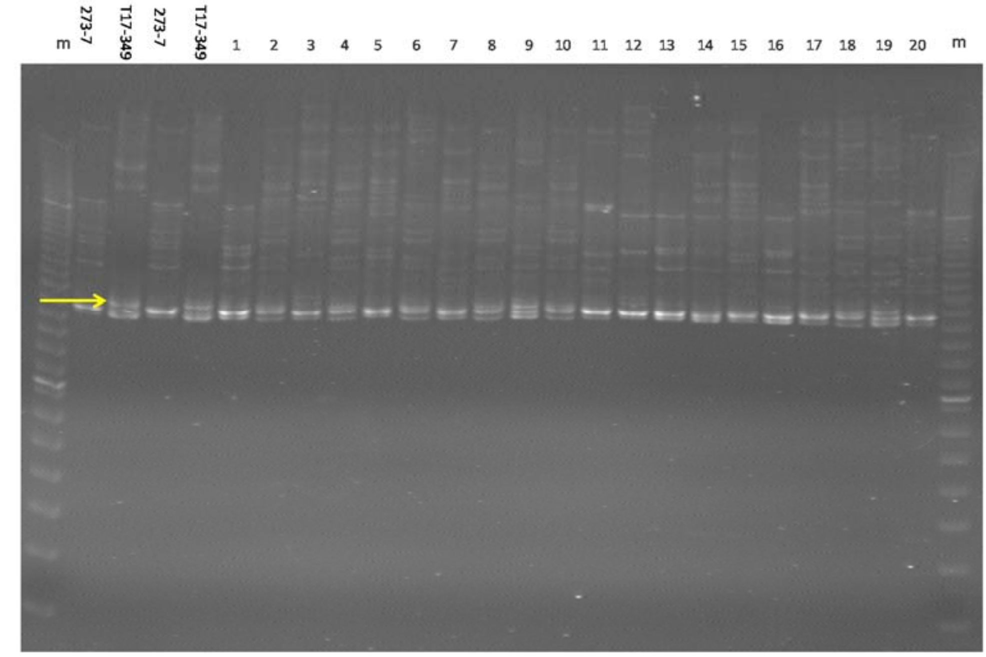

WCS0064

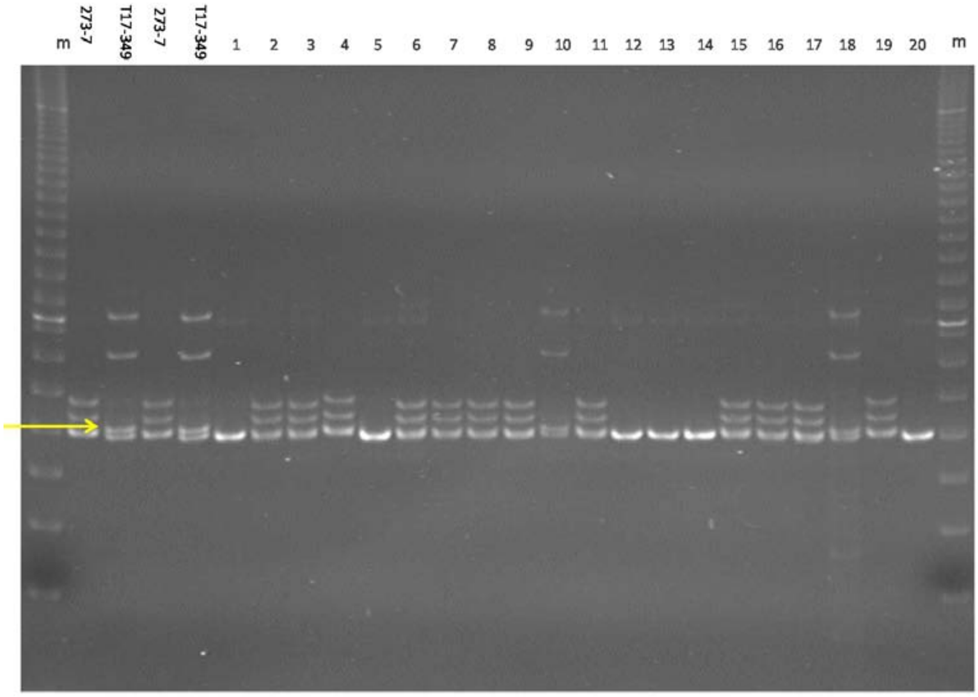

WCS0080c

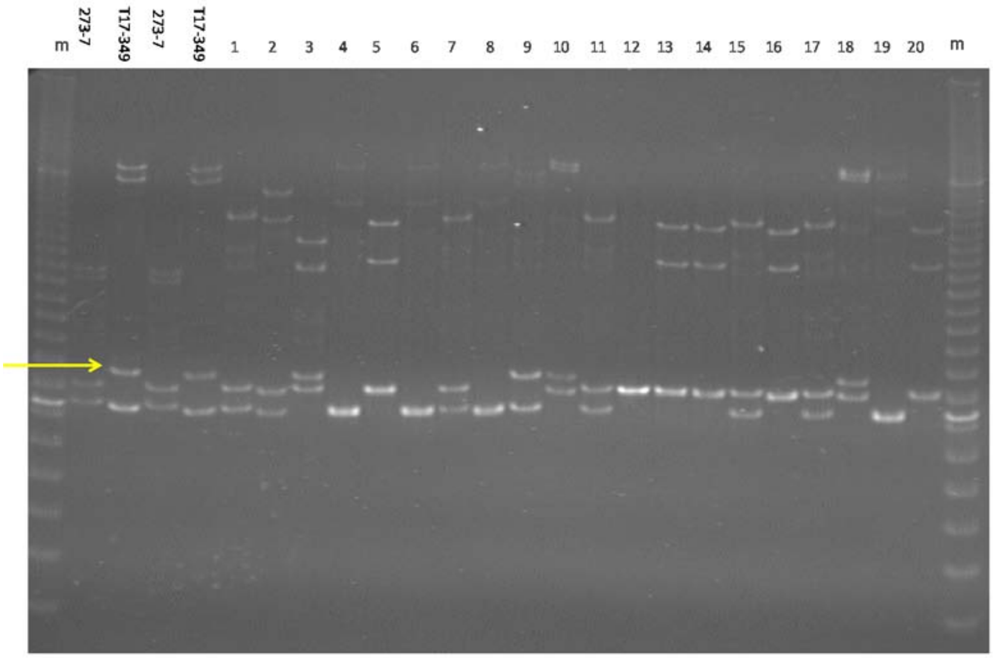

WCS1870b

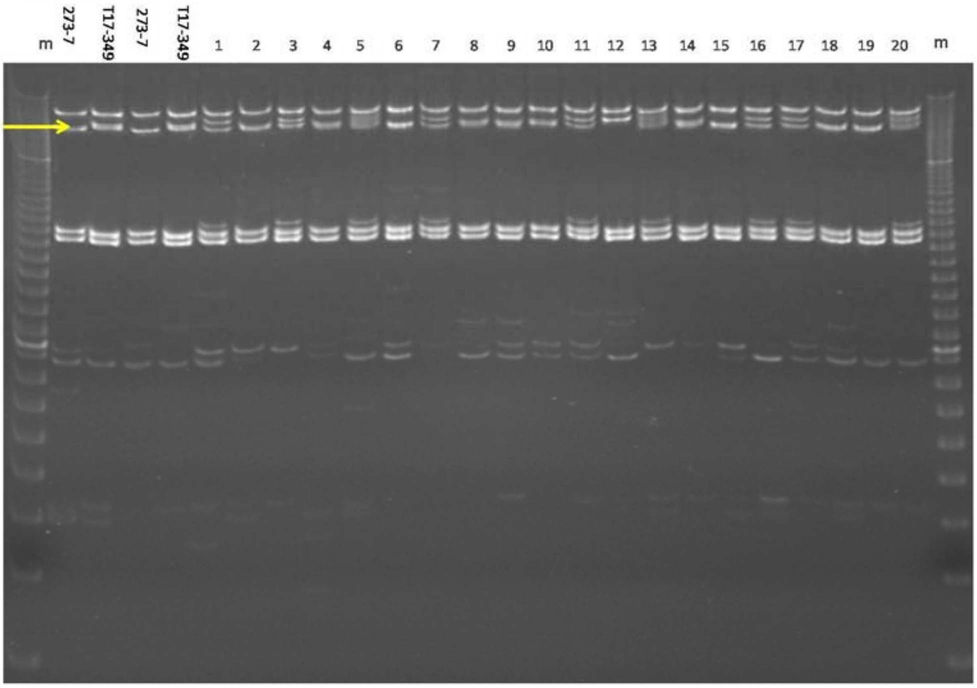

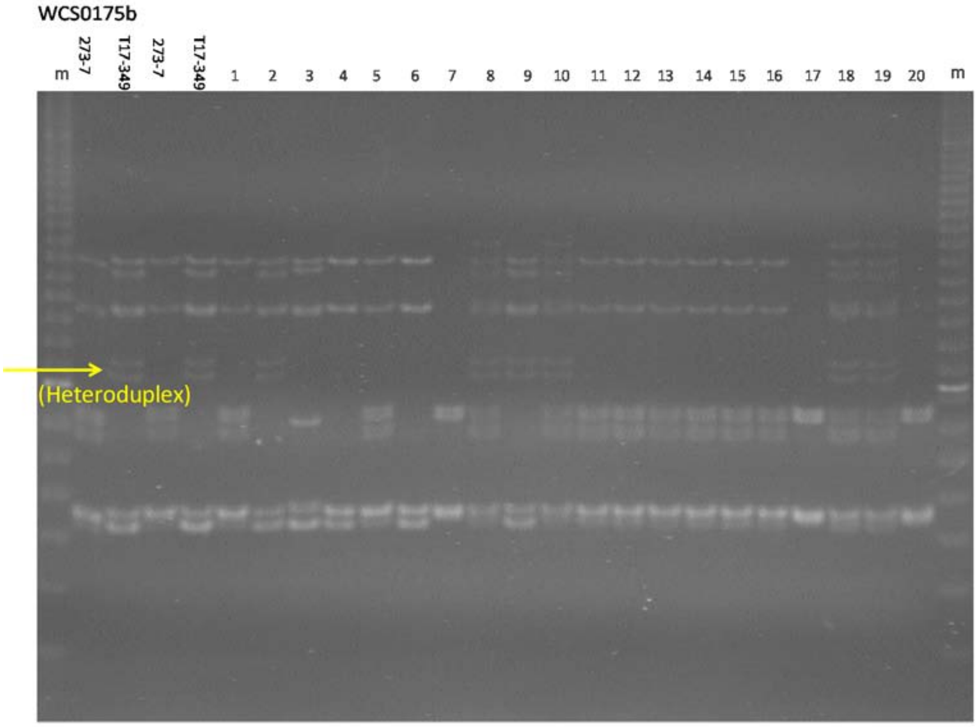

Figure S5 List of loci with identified LD showing r^2>0.5 between loci mapped onto different linkage groups, and photographs of acrylamide gel electrophoresis of PCR amplicons of the listed loci. The numbers above the acrylamide gel lanes represent IDs of F_1 individuals; m represents 20 bp ladder markers. Yellow arrows indicate scored bands for locus genotyping. 'Heteroduplex' shows bands used for scoring loci but predicted as heteroduplex bands.

Supporting Tables

These supporting tables are available for download at

http://www.g3journal.org/lookup/suppl/doi:10.1534/g3.112.002600/-/DC1.

Table S1 Primer sequences, SSR motifs, and expected sizes of amplicons of white clover EST-SSR markers.

Table S2 Locus name, position, and segregation distortion of the integrated and parental-specific maps.

Table S3 List of the non-redundant white clover ESTs.

Table S4 Number of mapped multiple loci and loci exhibiting parental-specific polymorphisms.

Table S5 Locus name and position of an integrated linkage map of red clover developed from a cross between HR and R130.
