# Supplementary material for: Comparative Genetic Mapping and Discovery of Linkage Disequilibrium Across Linkage Groups in White Clover (Trifolium repens L.)
Source: G3 (Bethesda). 2012 May 1;2(5):607–17. doi: 10.1534/g3.112.002600 (PMC3362943; doi:10.1534/g3.112.002600)
Supplement: Supporting Information [file supp_2.5.607_FigureS1.pdf]

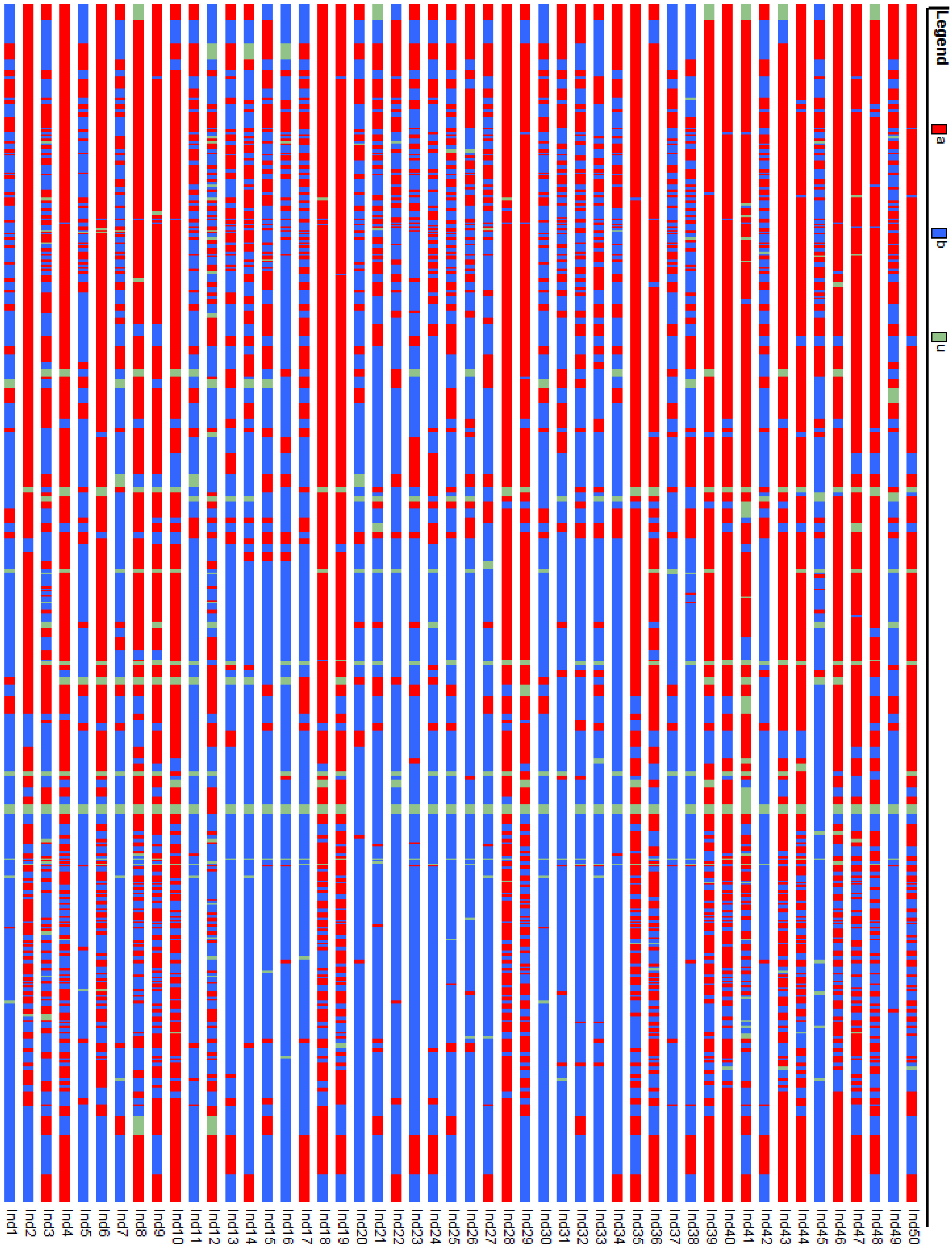

Figure S1 Graphical genotypes of the largest linkage groups of the 'T17-349'-specific map constructed before disassembling the locus data. The linkage group consisted of a total of 296 loci. Graphical genotypes are shown for 50 of the 188 F_1 mapping population. Red and blue colors show the two haplotypes of 'T17-349'. Light green color shows missing data.
