# Supplementary material for: Comparative Genetic Mapping and Discovery of Linkage Disequilibrium Across Linkage Groups in White Clover (Trifolium repens L.)
Source: G3 (Bethesda). 2012 May 1;2(5):607–17. doi: 10.1534/g3.112.002600 (PMC3362943; doi:10.1534/g3.112.002600)
Supplement: Supporting Information [file supp_2.5.607_FigureS2.pdf]

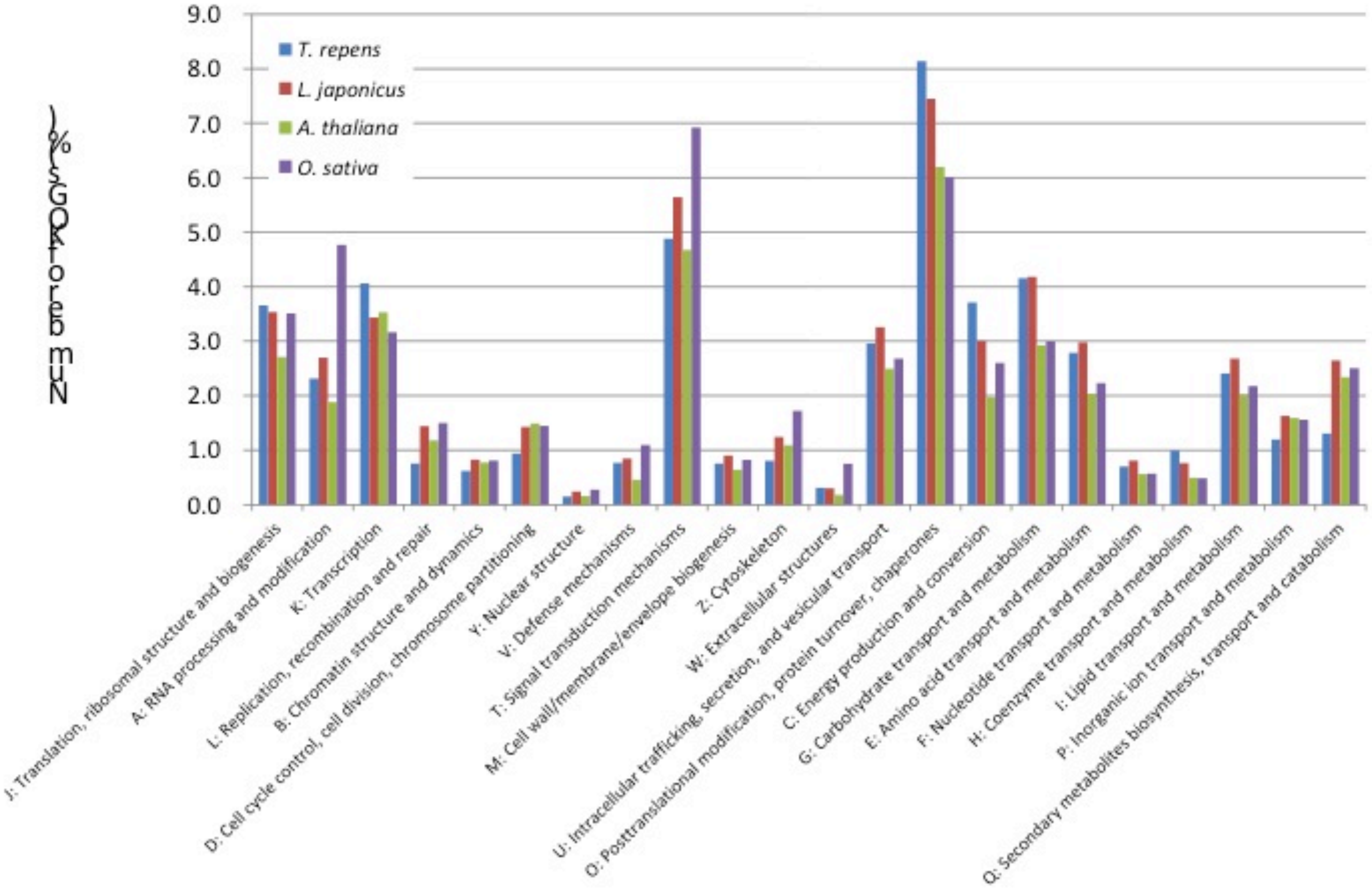

Figure S2 Functional classification of non-redundant EST sequences of *T. repens* and unigene sequences of *L. japonicus*, *A. thaliana*, and *O. sativa* based on KOG categories. BLASTX was used to compare the non-redundant EST sequences with the sequences in the KOG database. EST sequences were then classified into the KOG categories with the most similar sequences. The total number of KOG associated ESTs sequences and non-associated were 5193 and 2789, respectively.
