# Supplementary material for: Comparative Genetic Mapping and Discovery of Linkage Disequilibrium Across Linkage Groups in White Clover (Trifolium repens L.)
Source: G3 (Bethesda). 2012 May 1;2(5):607–17. doi: 10.1534/g3.112.002600 (PMC3362943; doi:10.1534/g3.112.002600)
Supplement: Supporting Information [file supp_2.5.607_FigureS3.pdf]

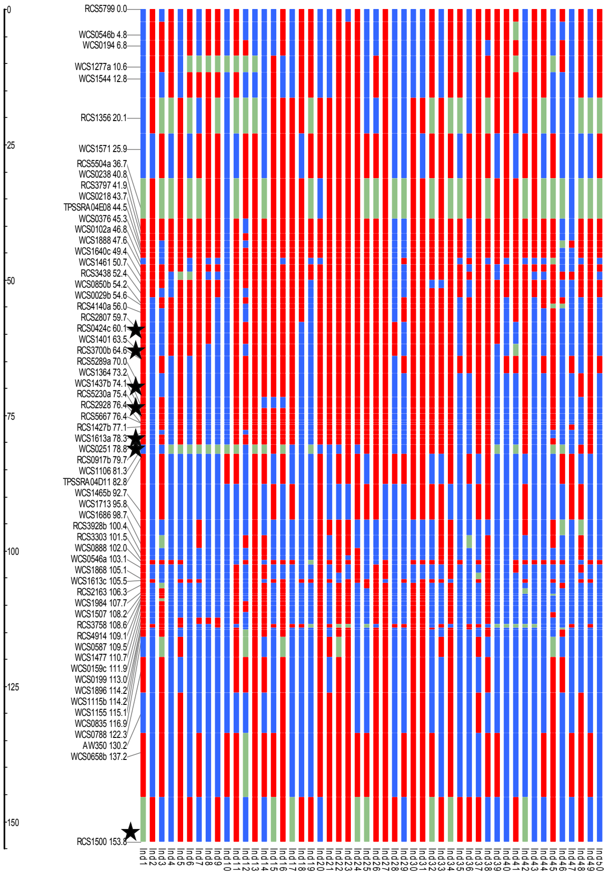

LG1a

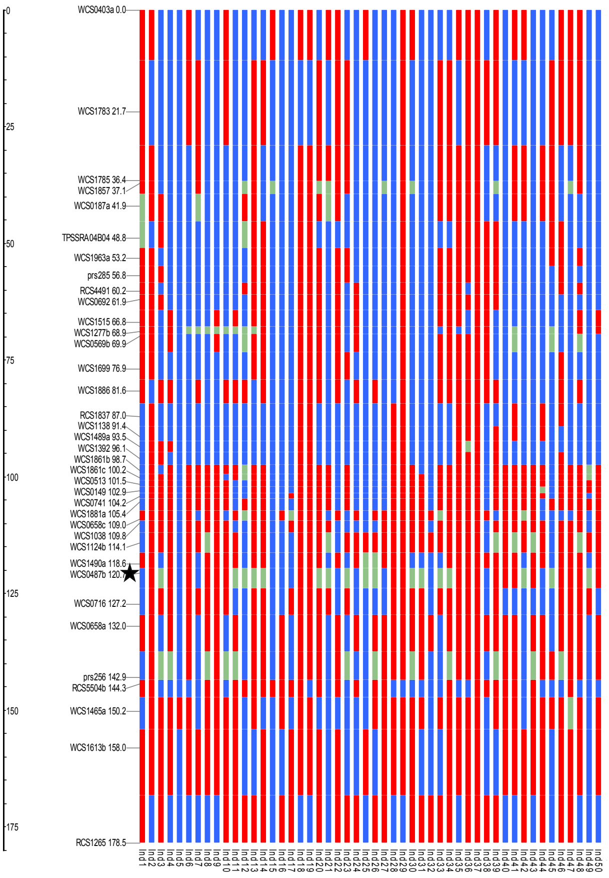

LG1b

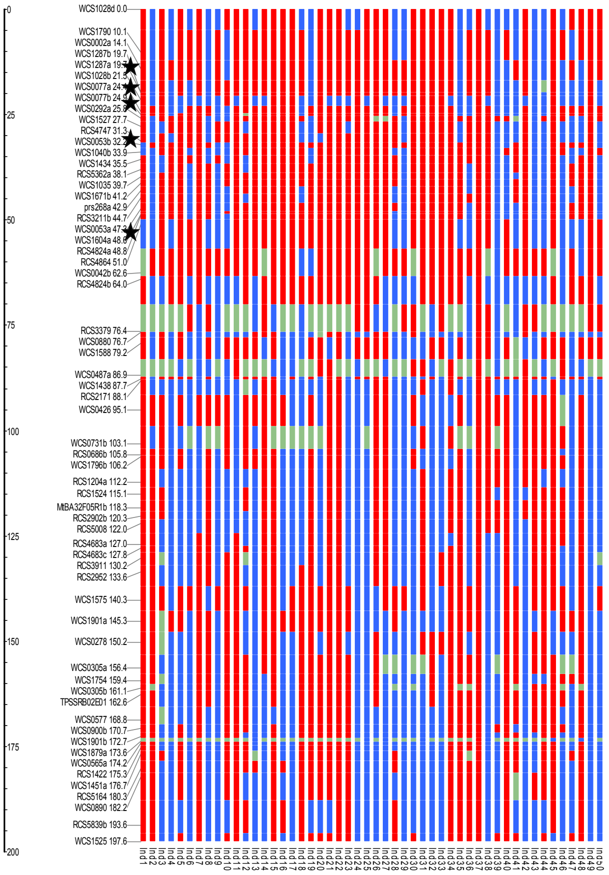
WCS1028d 0.0
WCS1790 10.1
WCS0002a 14.1
WCS1287b 19.7
WCS1028b 21.5
WCS1527 27.7
RCS4747 31.3
WCS1040b 33.9
WCS1434 35.5
RCS5362a 38.1
WCS1035 39.7
WCS1671b 41.2
prs268a 42.9
RCS3211b 44.7
WCS1604a 48.8
RCS4824a 48.8
RCS4864 51.0
WCS0042b 62.6
RCS4824b 64.0
RCS3379 76.4
WCS0880 76.7
WCS1588 79.2
WCS0487a 86.9
WCS1438 87.7
RCS2171 88.1
WCS0426 95.1
WCS0731b 103.1
RCS0686b 105.8
WCS1796b 106.2
RCS1204a 112.2
RCS1524 115.1
MtBA32F05R1b 118.3
RCS2902b 120.3
RCS5008 122.0
RCS4683a 127.0
RCS4683c 127.8
RCS3911 130.2
RCS2952 133.6
WCS1575 140.3
WCS1901a 145.3
WCS0278 150.2
WCS0305a 156.4
WCS1754 159.4
WCS0305b 161.1
TPSSRB02ED1 162.6
WCS0577 168.8
WCS0900b 170.7
WCS1901b 172.7
WCS1879a 173.6
WCS0565a 174.2
RCS1422 175.3
WCS1451a 176.7
RCS5164 180.3
WCS0890 182.2
RCS5839b 193.6
WCS1525 197.6
cM
LG2a

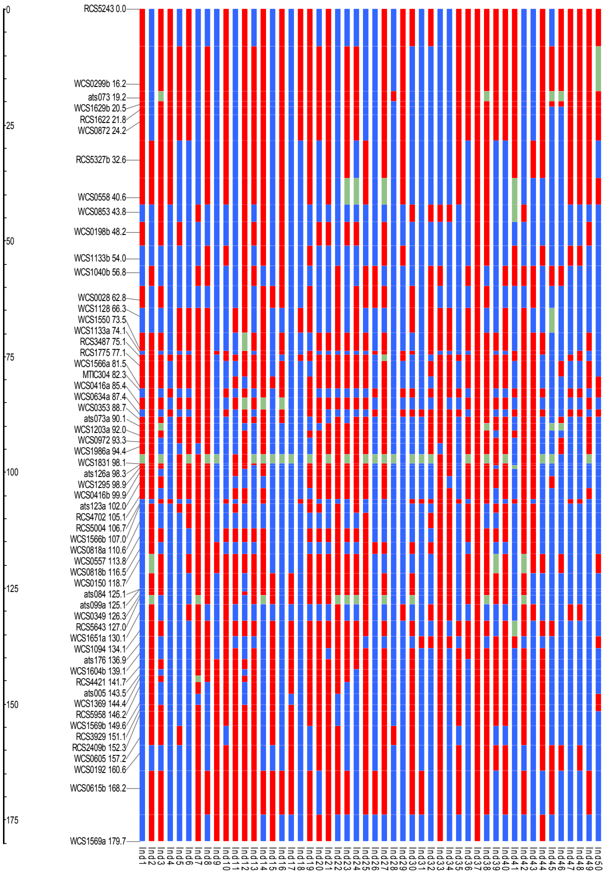
RCS5243 0.0
WCS0299b 16.2
ats073 19.2
WCS1629b 20.5
RCS1622 21.8
WCS0872 24.2
RCS5327b 32.6
WCS0558 40.6
WCS0853 43.8
WCS0198b 48.2
WCS1133b 54.0
WCS1040b 56.8
WCS0028 62.8
WCS1128 66.3
WCS1550 73.5
WCS1133a 74.1
RCS3487 75.1
RCS1775 77.1
WCS1566a 81.5
MTIC304 82.3
WCS0416a 85.4
WCS0634a 87.4
WCS0353 88.7
ats073a 90.1
WCS1203a 92.0
WCS0972 93.3
WCS1986a 94.4
WCS1831 98.1
ats126a 98.3
WCS1295 98.9
WCS0416b 99.9
ats123a 102.0
RCS4702 105.1
RCS5004 106.7
WCS1566b 107.0
WCS0818a 110.6
WCS0557 113.8
WCS0818b 116.5
WCS0150 118.7
ats084 125.1
ats099a 125.1
WCS0349 126.3
RCS5643 127.0
WCS1651a 130.1
WCS1094 134.1
ats176 136.9
WCS1604b 139.1
RCS4421 141.7
ats005 143.5
WCS1369 144.4
RCS5958 146.2
WCS1569b 149.6
RCS3929 151.1
RCS2409b 152.3
WCS0605 157.2
WCS0192 160.6
WCS0615b 168.2
WCS1569a 179.7
cM
LG2b

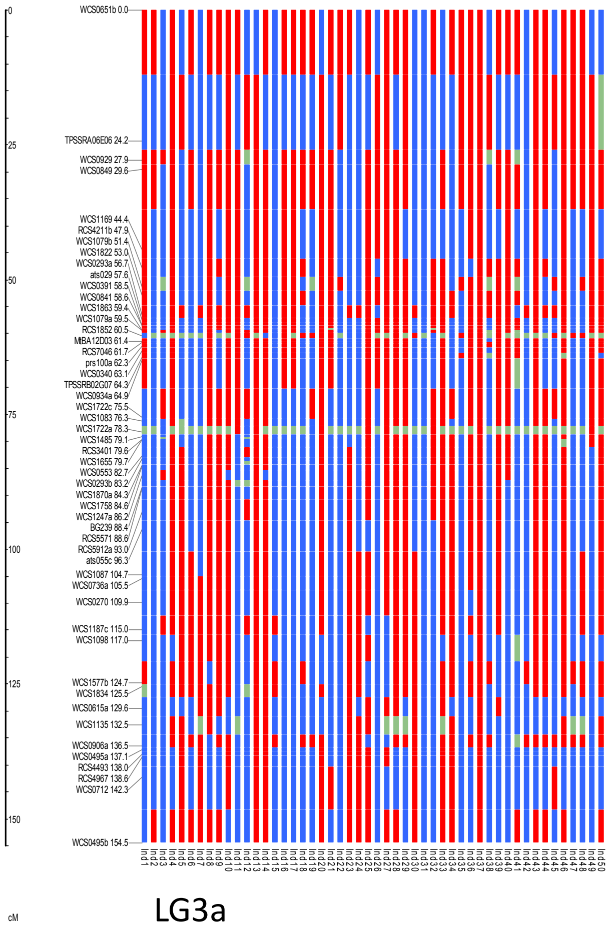

WCS0651b 0.0
TPSSRA06E06 24.2
WCS0929 27.9
WCS0849 29.6
WCS1169 44.4
RCS4211b 47.9
WCS1079b 51.4
WCS1822 53.0
WCS0293a 56.7
ats029 57.6
WCS0391 58.5
WCS0841 58.6
WCS1863 59.4
WCS1079a 59.5
RCS1852 60.5
MIBA12D03 61.4
RCS7046 61.7
prs100a 62.3
WCS0340 63.1
TPSSRB02G07 64.3
WCS0934a 64.9
WCS1722c 75.5
WCS1083 76.3
WCS1722a 78.3
WCS1485 79.1
RCS3401 79.6
WCS1655 79.7
WCS0553 82.7
WCS0293b 83.2
WCS1870a 84.3
WCS1758 84.6
WCS1247a 86.2
BG239 88.4
RCS5571 88.6
RCS5912a 93.0
ats055c 96.3
WCS1087 104.7
WCS0736a 105.5
WCS0270 109.9
WCS1187c 115.0
WCS1098 117.0
WCS1577b 124.7
WCS1834 125.5
WCS0615a 129.6
WCS1135 132.5
WCS0906a 136.5
WCS0495a 137.1
RCS4493 138.0
RCS4967 138.6
WCS0712 142.3
WCS0495b 154.5
cM
LG3a

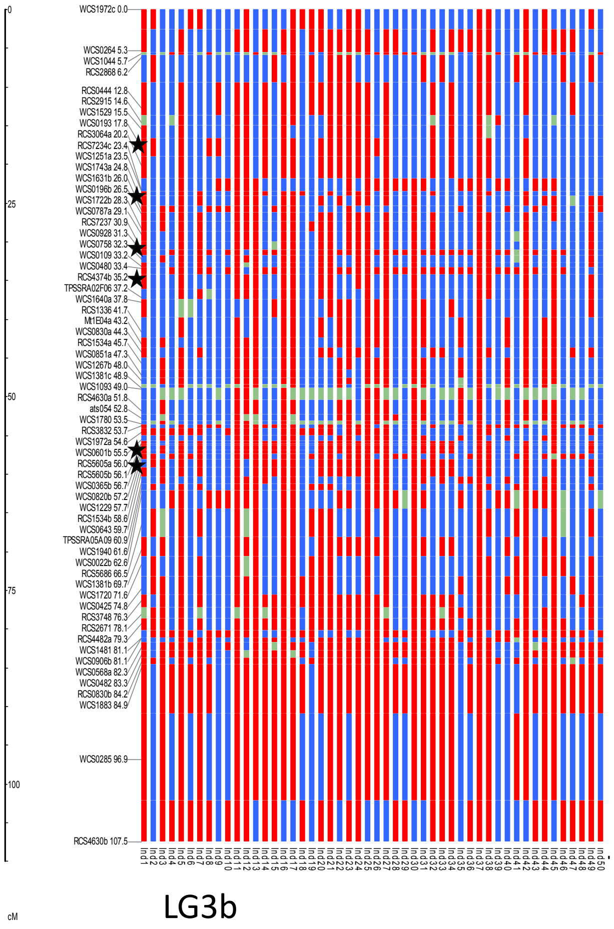

WCS1972c 0.0
WCS0264 5.3
WCS1044 5.7
RCS2868 6.2
RCS0444 12.8
RCS2915 14.6
WCS1529 15.5
WCS0193 17.8
RCS3064a 20.2
RCS7234c 23.4
WCS1251a 23.5
WCS1743a 24.8
WCS1631b 26.0
WCS0196b 26.5
WCS1722b 28.3
WCS0787a 29.1
RCS7237 30.9
WCS0928 31.3
WCS0758 32.3
WCS0109 33.2
WCS0480 33.4
RCS4374b 35.2
TPSSRA02F06 37.2
WCS1640a 37.8
RCS1336 41.7
Mt1E04a 43.2
WCS0830a 44.3
RCS1534a 45.7
WCS0851a 47.3
WCS1267b 48.0
WCS1381c 48.9
WCS1093 49.0
RCS4630a 51.8
ats054 52.8
WCS1780 53.5
RCS3832 53.7
WCS1972a 54.6
WCS0601b 55.5
RCS5605a 56.0
RCS5605b 56.1
WCS0365b 56.7
WCS0820b 57.2
WCS1229 57.7
RCS1534b 58.6
WCS0643 59.7
TPSSRA05A09 60.9
WCS1940 61.6
WCS0022b 62.6
RCS5686 66.5
WCS1381b 69.7
WCS1720 71.6
WCS0425 74.8
RCS3748 76.3
RCS2671 78.1
RCS4482a 79.3
WCS1481 81.1
WCS0906b 81.1
WCS0568a 82.3
WCS0482 83.3
RCS0830b 84.2
WCS1883 84.9
WCS0285 96.9
RCS4630b 107.5
cM
LG3b

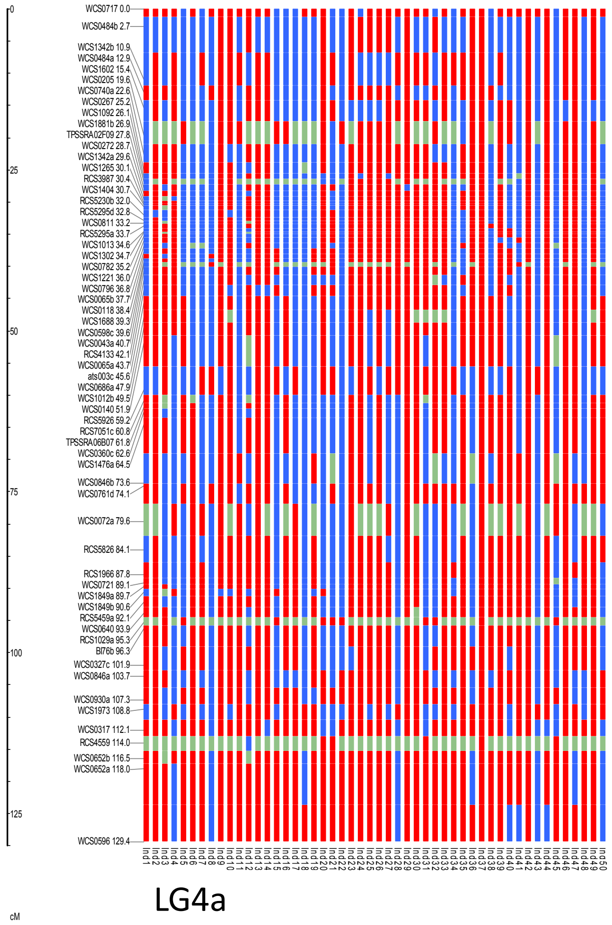
0
25
50
75
100
125
cM
WCS0717 0.0
WCS0484b 2.7
WCS1342b 10.9
WCS0484a 12.9
WCS1602 15.4
WCS0205 19.6
WCS0740a 22.6
WCS0267 25.2
WCS1092 26.1
WCS1881b 26.9
TPSSRA02F09 27.8
WCS0272 28.7
WCS1342a 29.6
WCS1265 30.1
RCS3987 30.4
WCS1404 30.7
RCS5230b 32.0
RCS5295d 32.8
WCS0811 33.2
RCS5295a 33.7
WCS1013 34.6
WCS1302 34.7
WCS0782 35.2
WCS1221 36.0
WCS0796 36.8
WCS0065b 37.7
WCS0118 38.4
WCS1688 39.3
WCS0598c 39.6
WCS0043a 40.7
RCS4133 42.1
WCS0065a 43.7
ats003c 45.6
WCS0686a 47.9
WCS1012b 49.5
WCS0140 51.9
RCS5926 59.2
RCS7051c 60.8
TPSSRA06B07 61.8
WCS0360c 62.6
WCS1476a 64.5
WCS0846b 73.6
WCS0761d 74.1
WCS0072a 79.6
RCS5826 84.1
RCS1966 87.8
WCS0721 89.1
WCS1849a 89.7
WCS1849b 90.6
RCS5459a 92.1
WCS0640 93.9
RCS1029a 95.3
BI76b 96.3
WCS0327c 101.9
WCS0846a 103.7
WCS0930a 107.3
WCS1973 108.8
WCS0317 112.1
RCS4559 114.0
WCS0652b 116.5
WCS0652a 118.0
WCS0596 129.4
Ind1 Ind2 Ind3 Ind4 Ind5 Ind6 Ind7 Ind8 Ind9 Ind10 Ind11 Ind12 Ind13 Ind14 Ind15 Ind16 Ind17 Ind18 Ind19 Ind20 Ind21 Ind22 Ind23 Ind24 Ind25 Ind26 Ind27 Ind28 Ind29 Ind30 Ind31 Ind32 Ind33 Ind34 Ind35 Ind36 Ind37 Ind38 Ind39 Ind40 Ind41 Ind42 Ind43 Ind44 Ind45 Ind46 Ind47 Ind48 Ind49 Ind50

LG4a

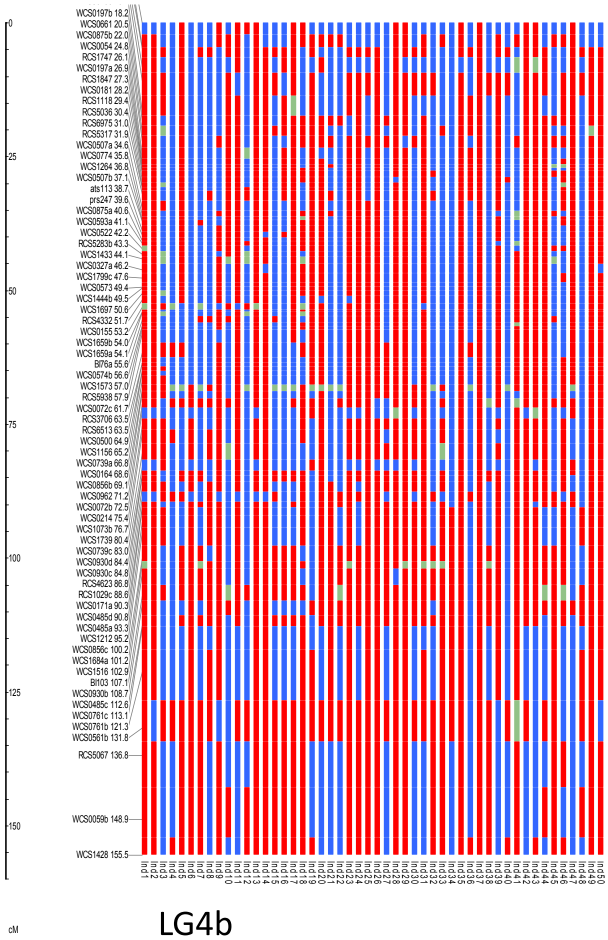
0
25
50
75
100
125
150
cM
WCS0197b 18.2
WCS0661 20.5
WCS0875b 22.0
WCS0054 24.8
RCS1747 26.1
WCS0197a 26.9
RCS1847 27.3
WCS0181 28.2
RCS1118 29.4
RCS5036 30.4
RCS6975 31.0
RCS5317 31.9
WCS0507a 34.6
WCS0774 35.8
WCS1264 36.8
WCS0507b 37.1
ats113 38.7
prs247 39.6
WCS0875a 40.6
WCS0593a 41.1
WCS0522 42.2
RCS5283b 43.3
WCS1433 44.1
WCS0327a 46.2
WCS1799c 47.6
WCS0573 49.4
WCS1444b 49.5
WCS1697 50.6
RCS4332 51.7
WCS0155 53.2
WCS1659b 54.0
WCS1659a 54.1
BI76a 55.6
WCS0574b 56.6
WCS1573 57.0
RCS5938 57.9
WCS0072c 61.7
RCS3706 63.5
RCS6513 63.5
WCS0500 64.9
WCS1156 65.2
WCS0739a 66.8
WCS0164 68.6
WCS0856b 69.1
WCS0962 71.2
WCS0072b 72.5
WCS0214 75.4
WCS1073b 76.7
WCS1739 80.4
WCS0739c 83.0
WCS0930d 84.4
WCS0930c 84.8
RCS4623 86.8
RCS1029c 88.6
WCS0171a 90.3
WCS0485d 90.8
WCS0485a 93.3
WCS1212 95.2
WCS0856c 100.2
WCS1684a 101.2
WCS1516 102.9
BI103 107.1
WCS0930b 108.7
WCS0485c 112.6
WCS0761c 113.1
WCS0761b 121.3
WCS0561b 131.8
RCS5067 136.8
WCS0059b 148.9
WCS1428 155.5
Ind1 Ind2 Ind3 Ind4 Ind5 Ind6 Ind7 Ind8 Ind9 Ind10 Ind11 Ind12 Ind13 Ind14 Ind15 Ind16 Ind17 Ind18 Ind19 Ind20 Ind21 Ind22 Ind23 Ind24 Ind25 Ind26 Ind27 Ind28 Ind29 Ind30 Ind31 Ind32 Ind33 Ind34 Ind35 Ind36 Ind37 Ind38 Ind39 Ind40 Ind41 Ind42 Ind43 Ind44 Ind45 Ind46 Ind47 Ind48 Ind49 Ind50

LG4b

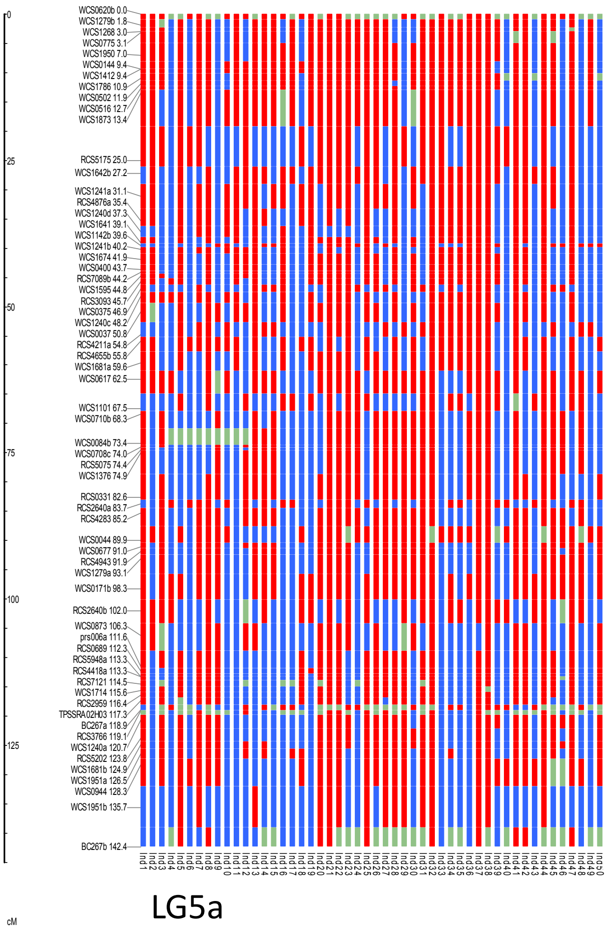

cM
LG5a

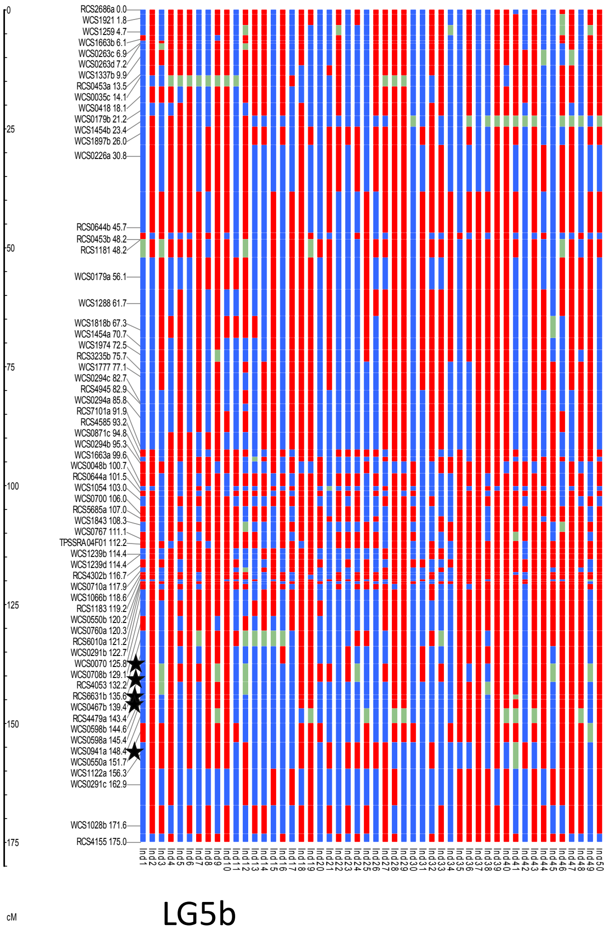

cM
LG5b

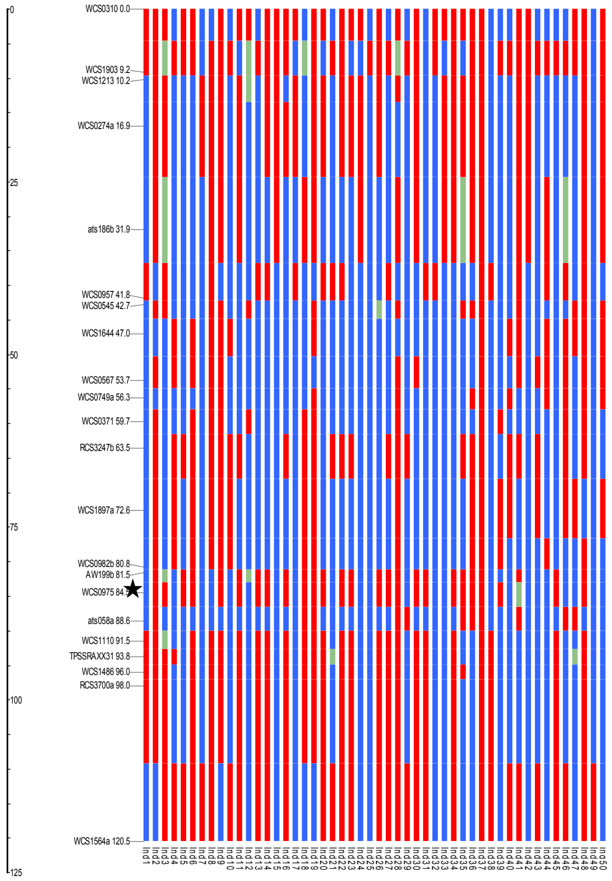
WCS0310 0.0
WCS1903 9.2
WCS1213 10.2
WCS0274a 16.9
ats186b 31.9
WCS0957 41.8
WCS0545 42.7
WCS1644 47.0
WCS0567 53.7
WCS0749a 56.3
WCS0371 59.7
RCS3247b 63.5
WCS1897a 72.6
WCS0982b 80.8
AW199b 81.5
ats058a 88.6
WCS1110 91.5
TPSSRAXX31 93.8
WCS1486 96.0
RCS3700a 98.0
WCS1564a 120.5
cM

LG6a

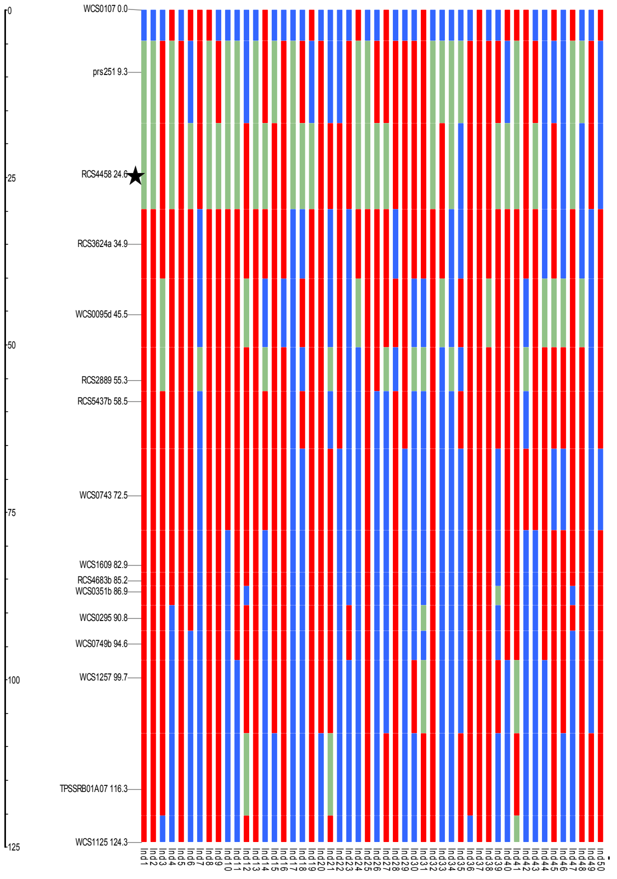
WCS0107 0.0
prs251 9.3
RCS4458 24.6
RCS3624a 34.9
WCS0095d 45.5
RCS2889 55.3
RCS5437b 58.5
WCS0743 72.5
WCS1609 82.9
RCS4683b 85.2
WCS0351b 86.9
WCS0295 90.8
WCS0749b 94.6
WCS1257 99.7
TPSSRB01A07 116.3
WCS1125 124.3
cM

LG6b

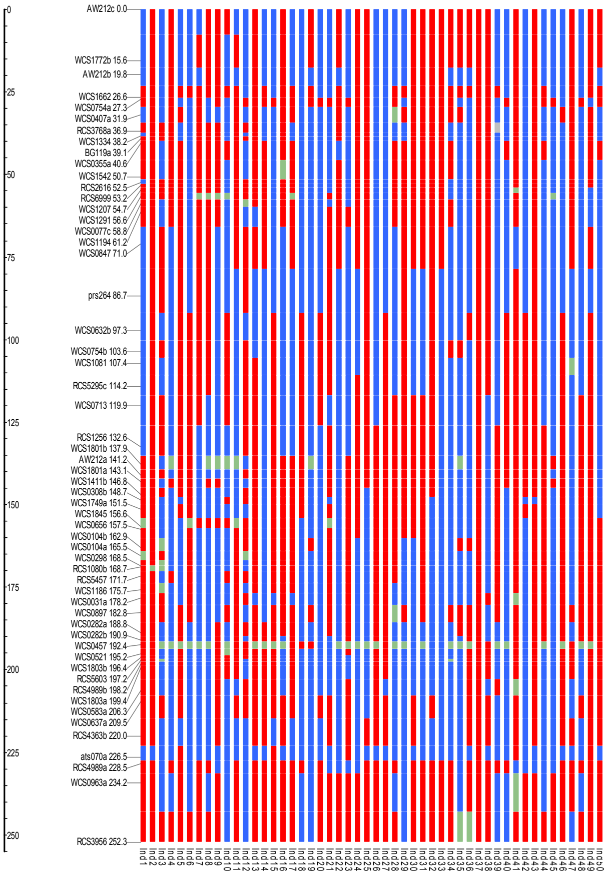

cM

LG7a

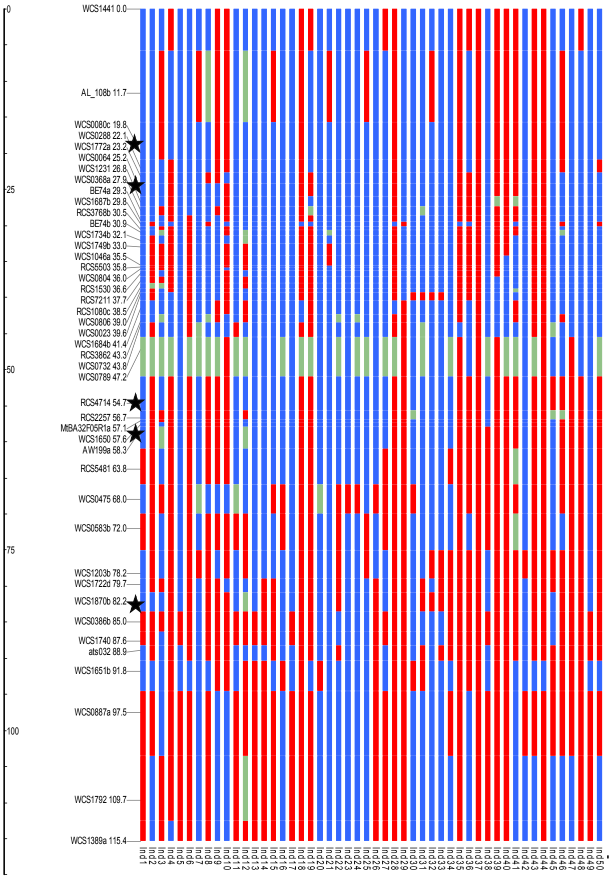

cM

LG7b

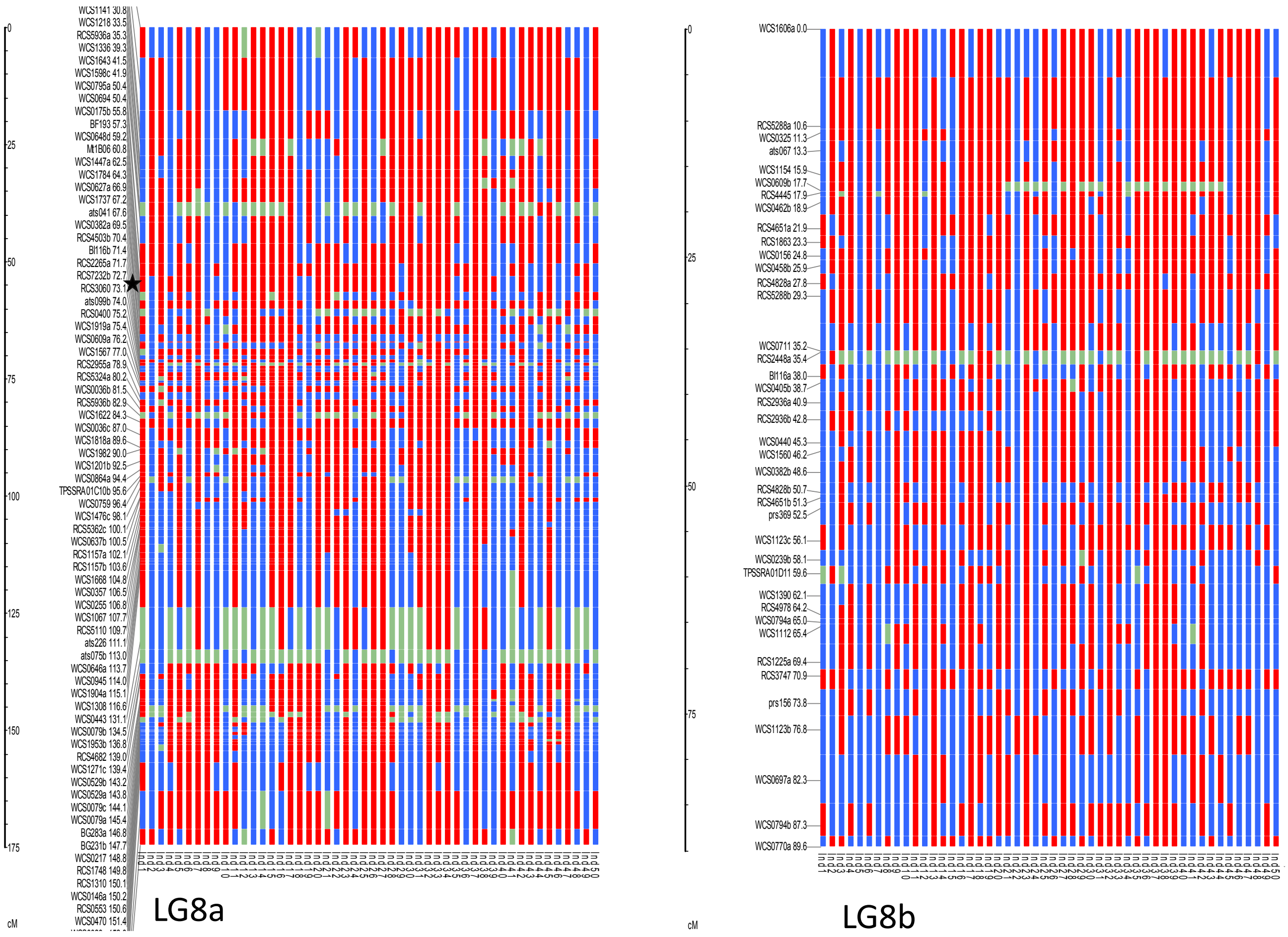

Figure S3 Graphical genotypes of 50 of the 188 F_1 populations mapped onto the largest linkage group shown in Figure S1. The red and blue colors show the two haplotypes of 'T17-349'. The light green color shows missing data. Black stars indicate loci showing a high LD (r^2>0.5) between loci mapped onto different linkage groups.
