# Supplementary material for: Comparative Genetic Mapping and Discovery of Linkage Disequilibrium Across Linkage Groups in White Clover (Trifolium repens L.)
Source: G3 (Bethesda). 2012 May 1;2(5):607–17. doi: 10.1534/g3.112.002600 (PMC3362943; doi:10.1534/g3.112.002600)
Supplement: Supporting Information [file supp_2.5.607_FigureS4.pdf]

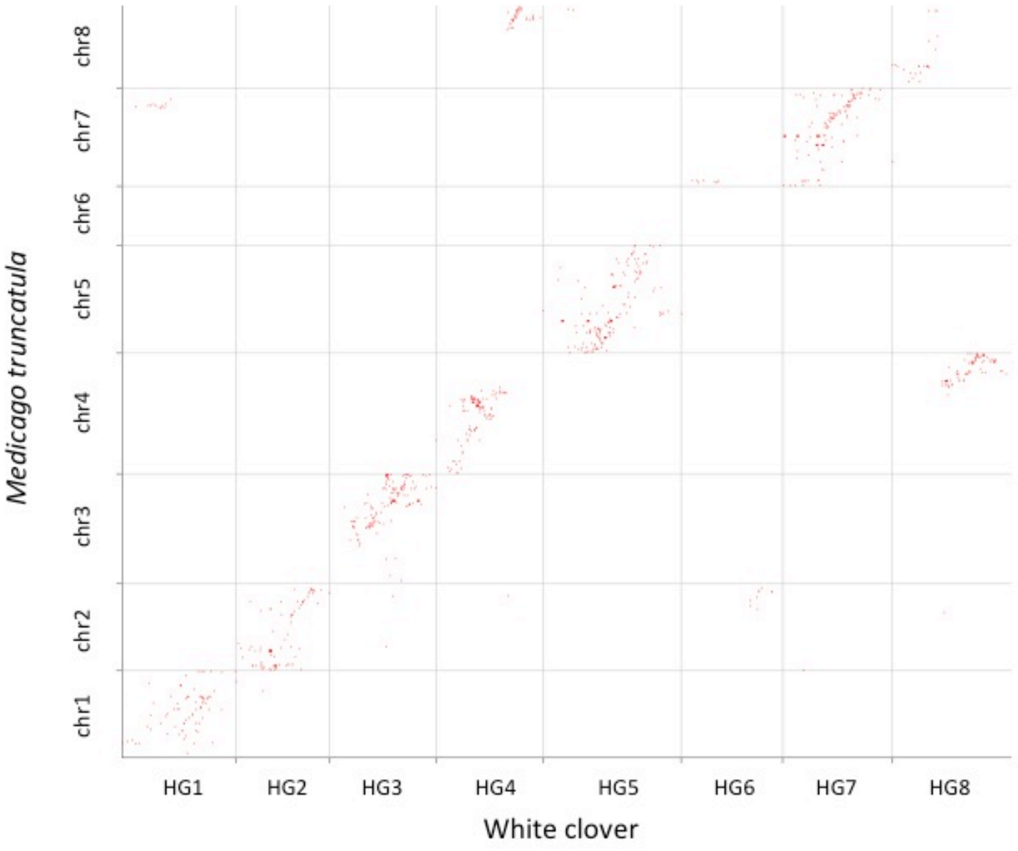

Figure S4 Graphical view of syntenic blocks between white clover and *M. truncatula*. X and Y axis show position on mapped markers on an white clover linkage map and chromosomes of *M. truncatula* genome, respectively.
