# Supplementary material for: Comparative Genetic Mapping and Discovery of Linkage Disequilibrium Across Linkage Groups in White Clover (Trifolium repens L.)
Source: G3 (Bethesda). 2012 May 1;2(5):607–17. doi: 10.1534/g3.112.002600 (PMC3362943; doi:10.1534/g3.112.002600)
Supplement: Supporting Information [file supp_2.5.607_FigureS5.pdf]

List of loci identified LD showing r2>0.5 between loci mapped onto different linkage groups.

Mapped LG	Locus name	Mapped LG	Locus name
1a	RCS1500	3b	WCS0643
1a	RCS2807	3b	WCS1743a
1a	RCS5289a	5b	RCS6631b
1a	WCS0251	5b	WCS0467b
1a	WCS1106	5b	WCS0598a
1a	WCS1364	5b	WCS0598b
1a	WCS1401	5b	WCS1122a
1b	WCS0487b	6a	WCS0975
2a	RCS4747	6b	RCS4458
2a	RCS4864	7b	ats032
2a	WCS0002a	7b	AW199a
2a	WCS0077a	7b	RCS4714
2a	WCS1287b	7b	WCS0064
3b	RCS3064a	7b	WCS0080c
3b	RCS4374b	7b	WCS1870b
3b	RCS5605a	8a	WCS0175b
3b	RCS7237		

RCS1500

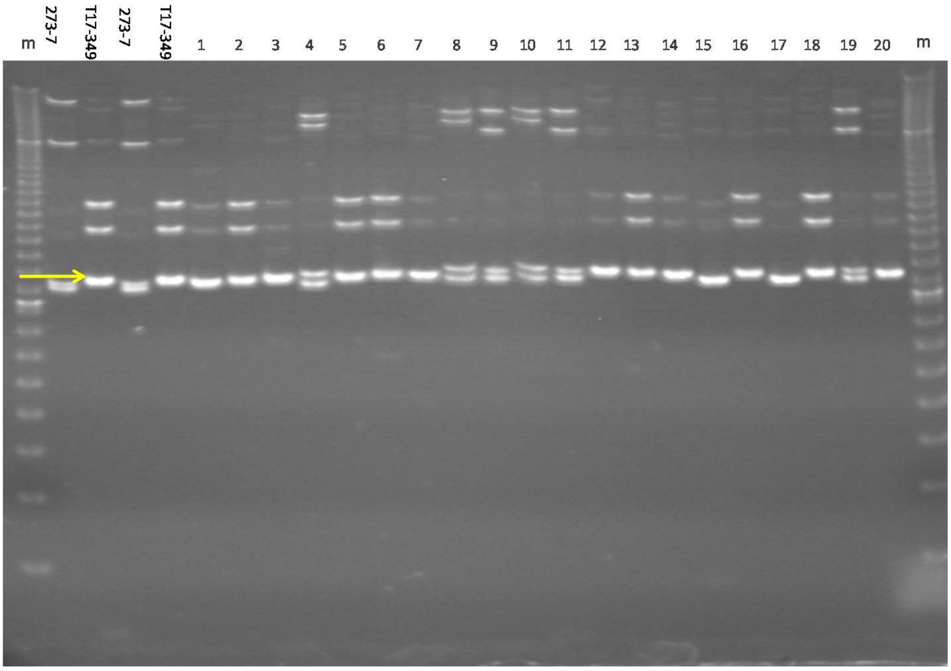

RCS2807

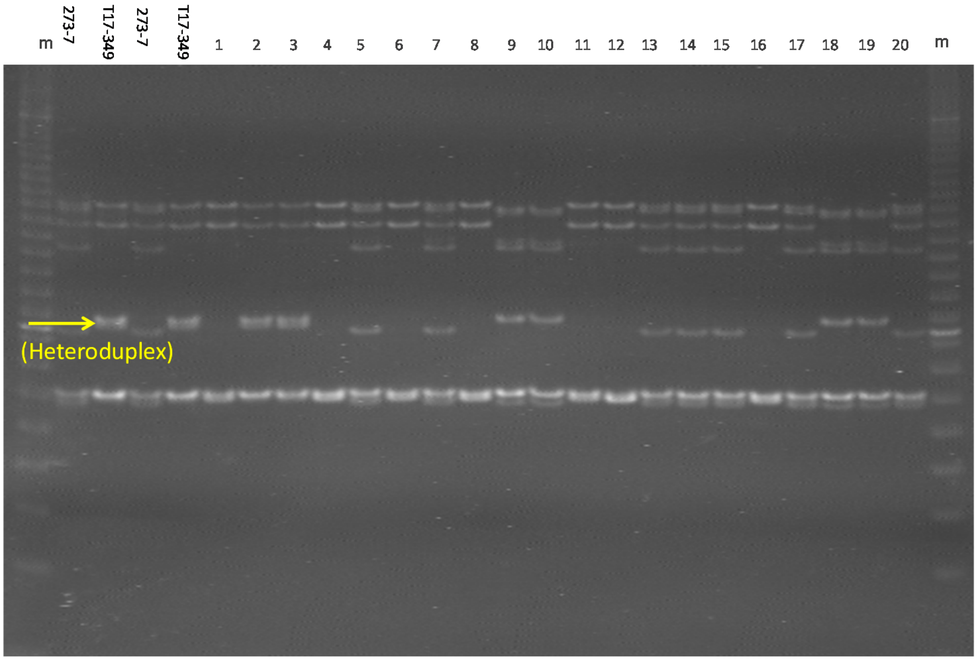

RCS5289a

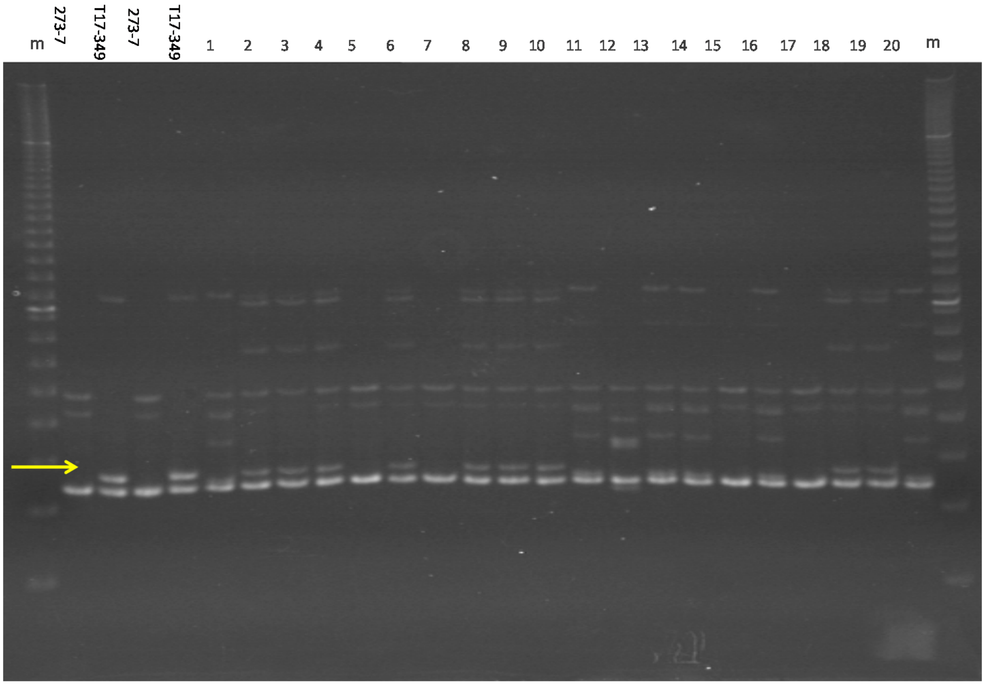

WCS0251

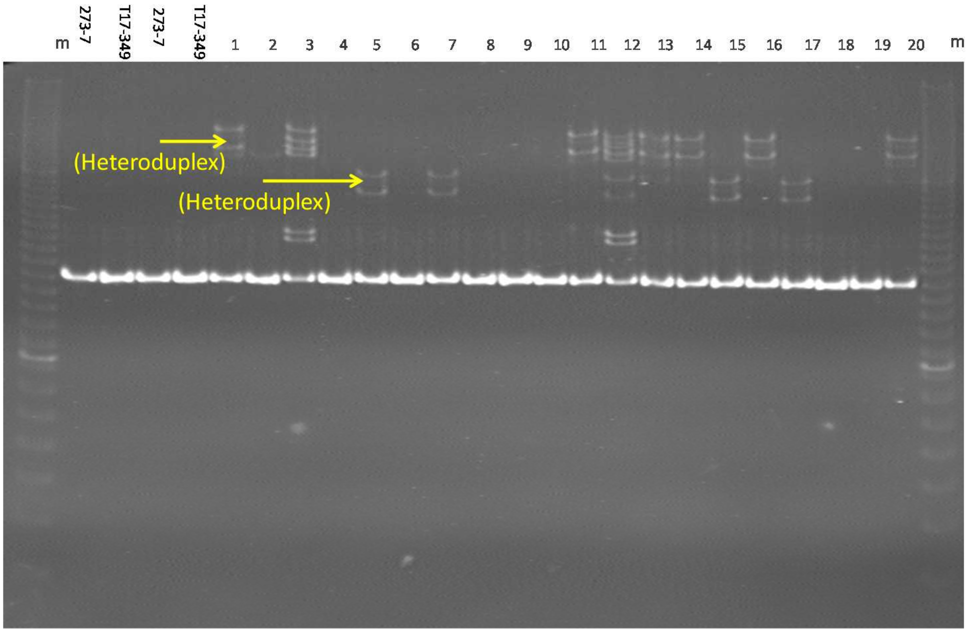

WCS1106

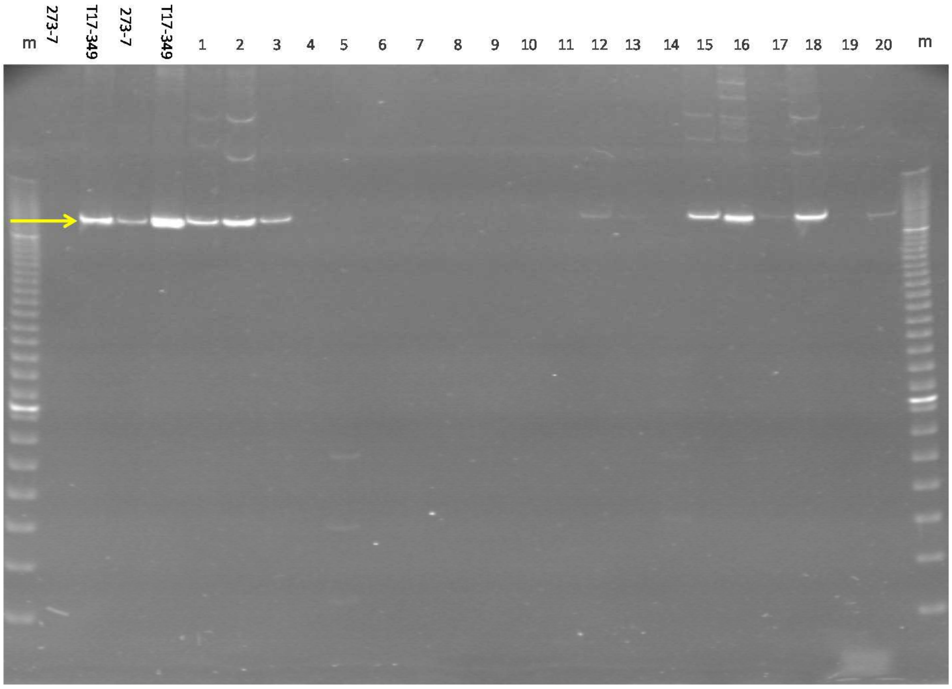

WCS1364

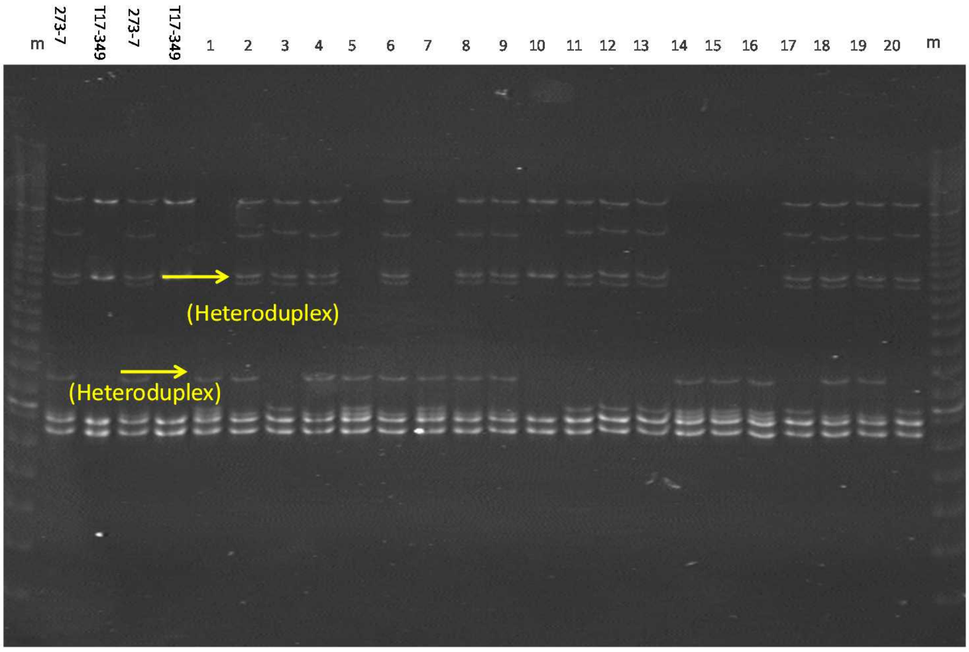

WCS1401

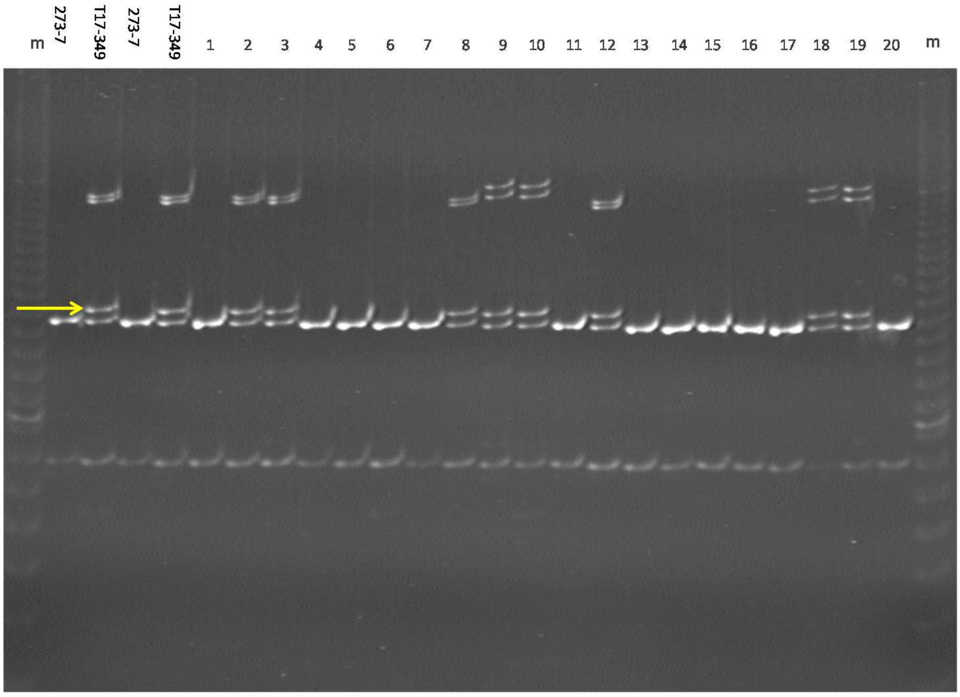

WCS0487b

m 273-7 T17-349 273-7 T17-349 1 2 3 4 5 6 7 8 9 10 11 12 13 14 15 16 17 18 19 20 m

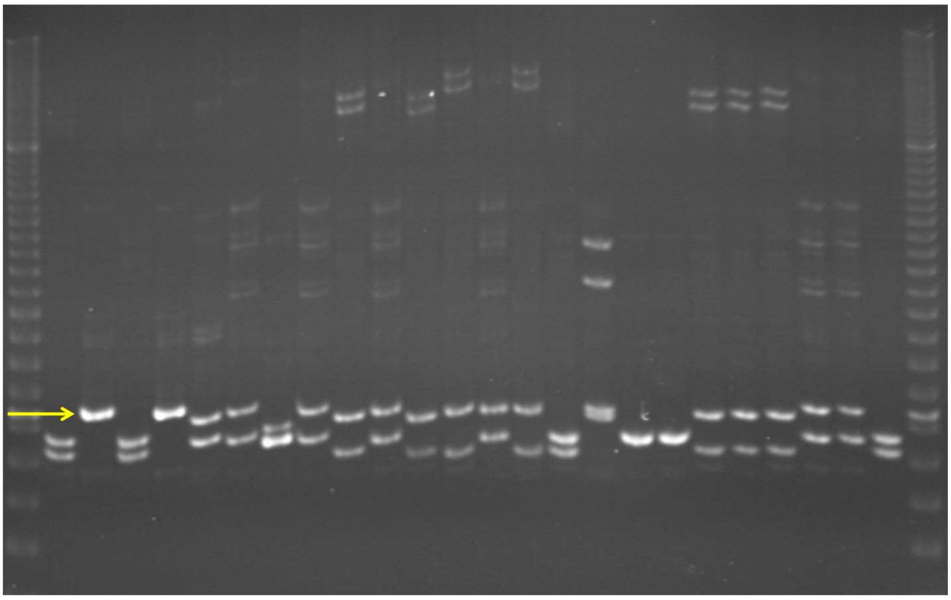

RCS4747

m 273-7 T17-349 273-7 T17-349 1 2 3 4 5 6 7 8 9 10 11 12 13 14 15 16 17 18 19 20 m

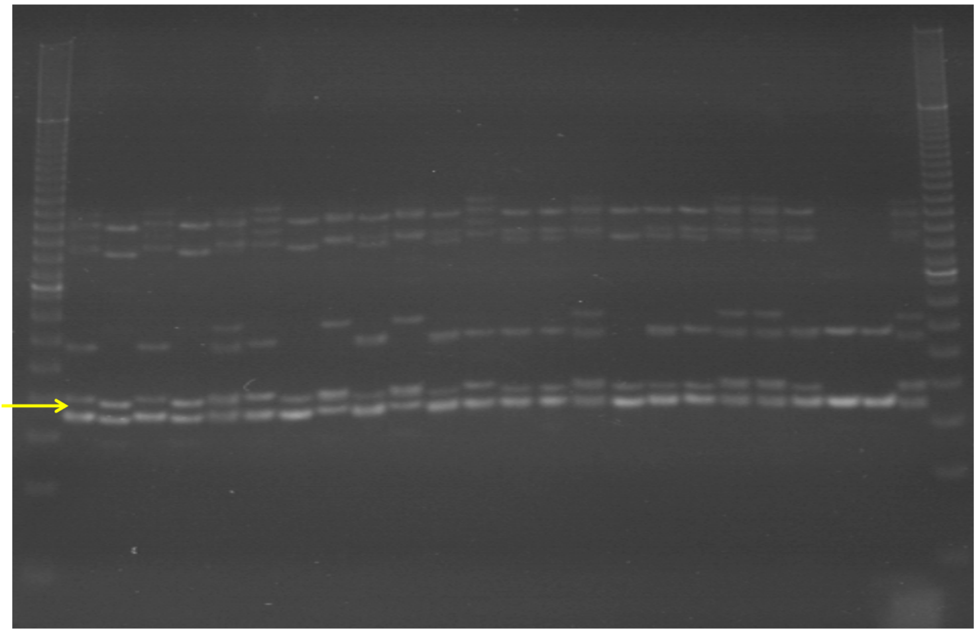

RCS4864

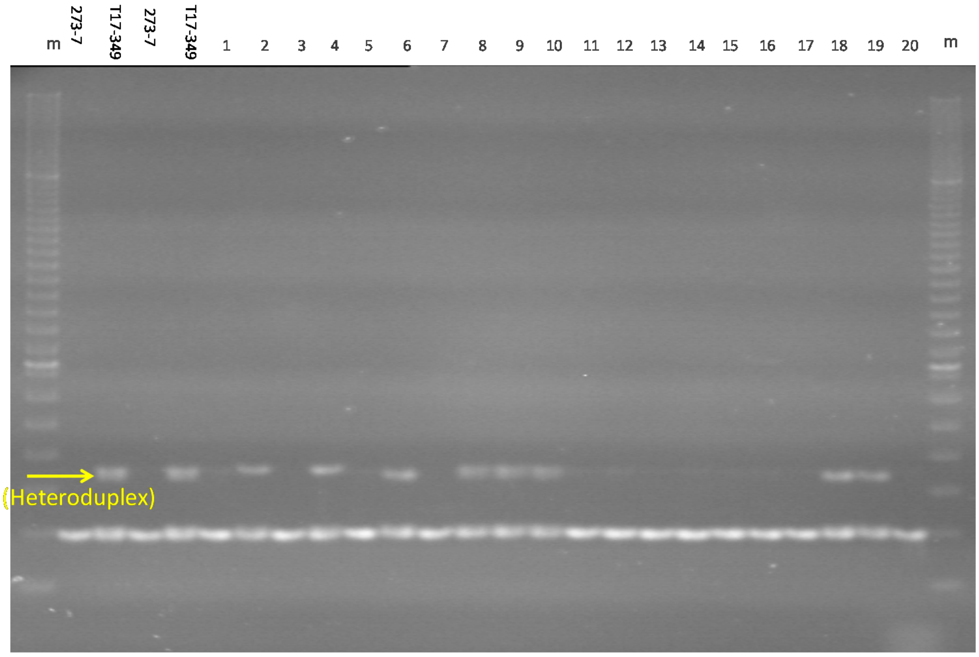

WCS0002a

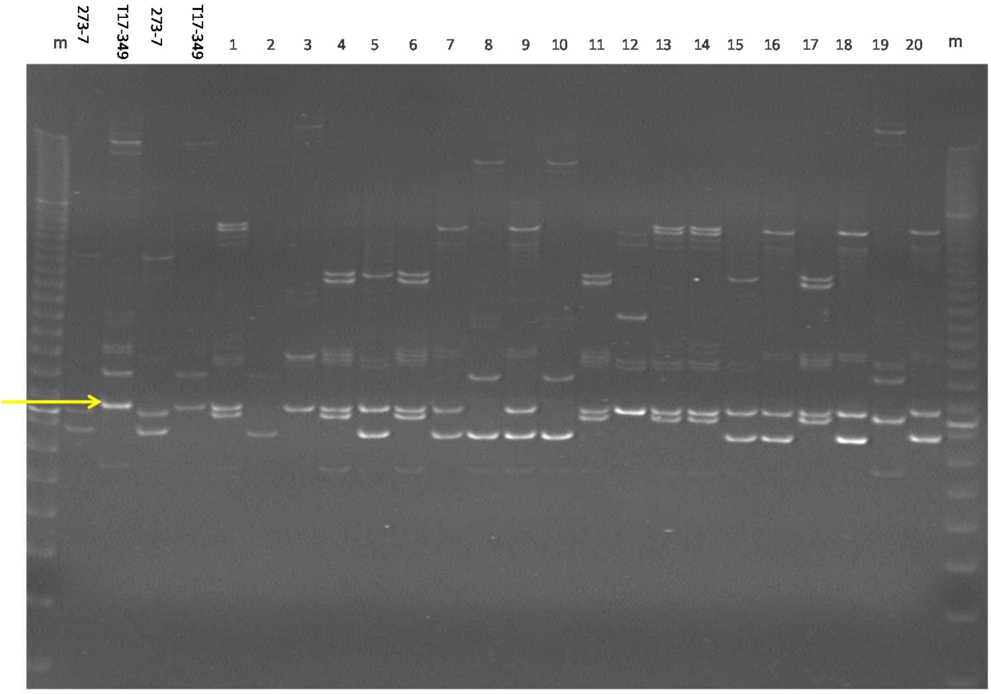

WCS00077a

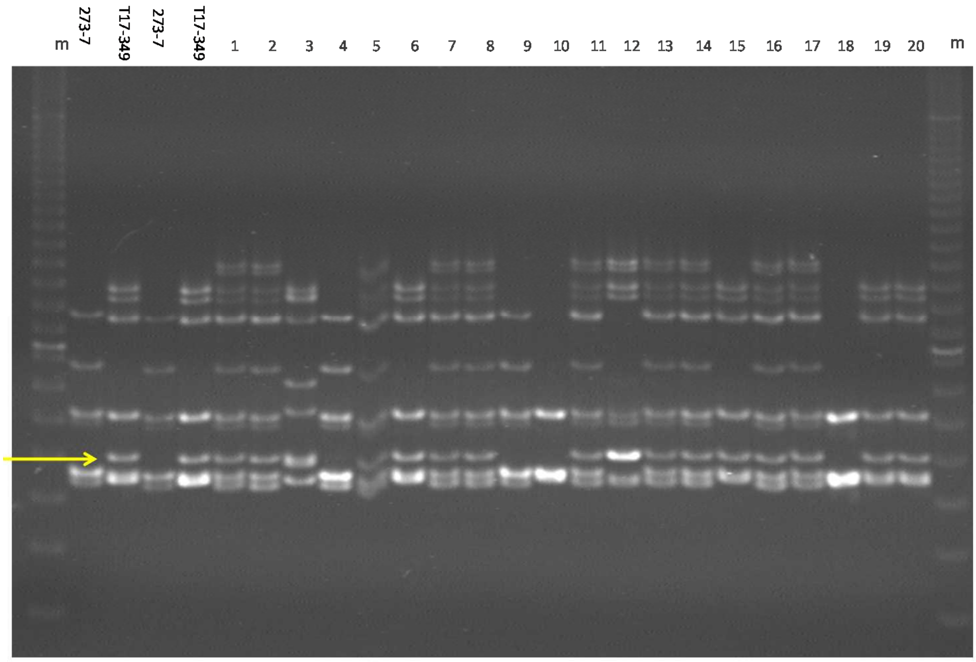

WCS1287b

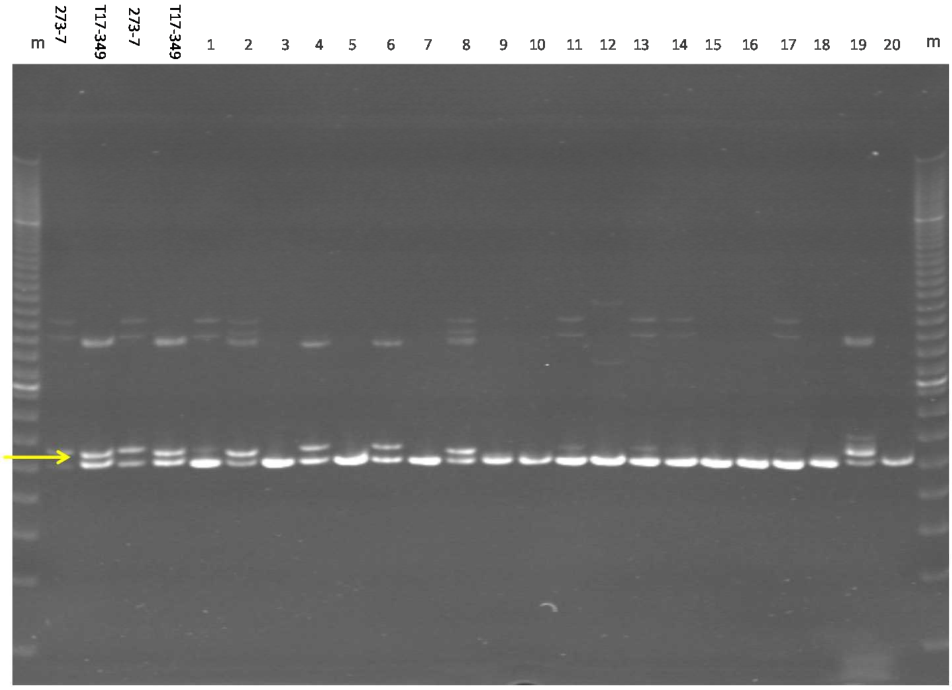

RCS3064a

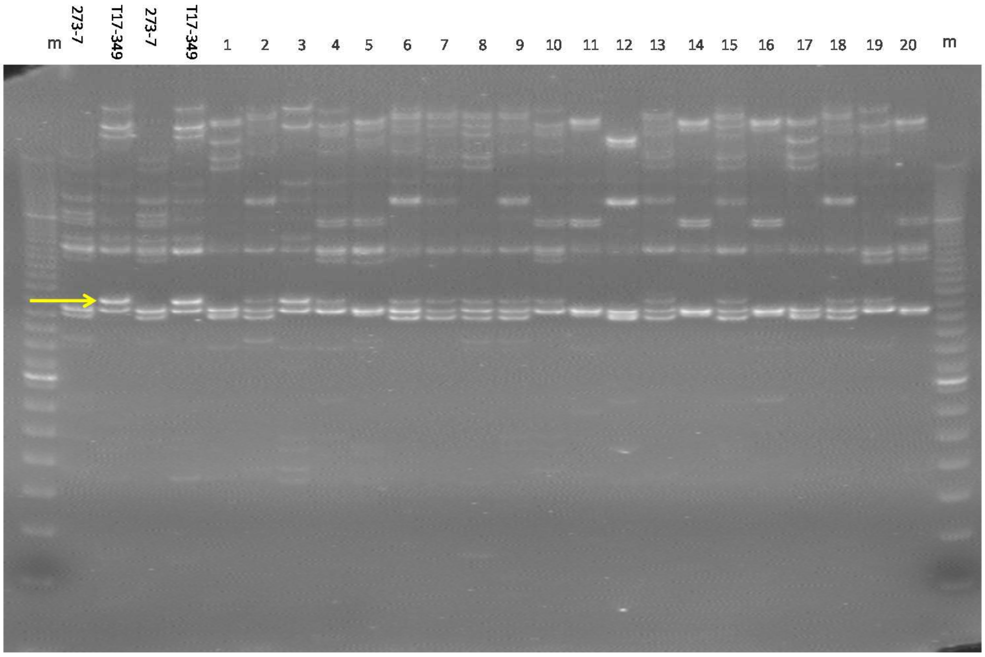

RCS4374b

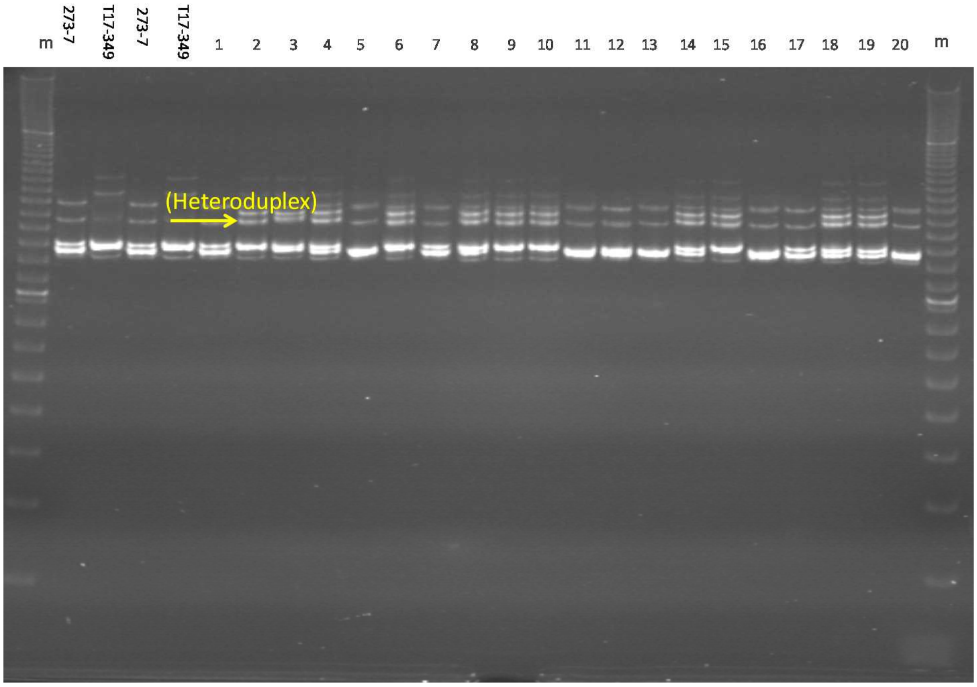

RCS5605a

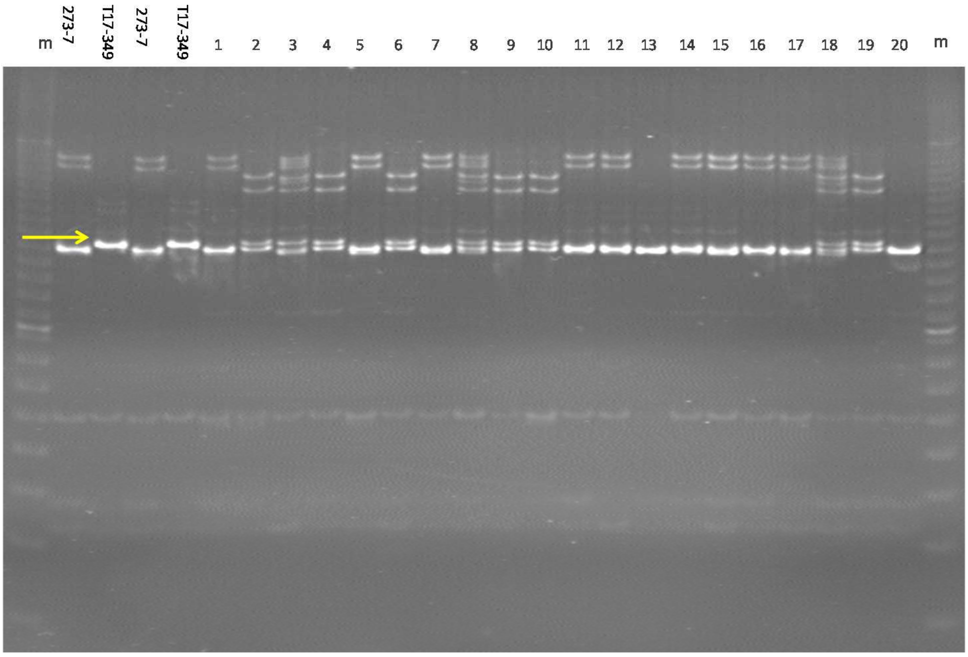

RCS7237

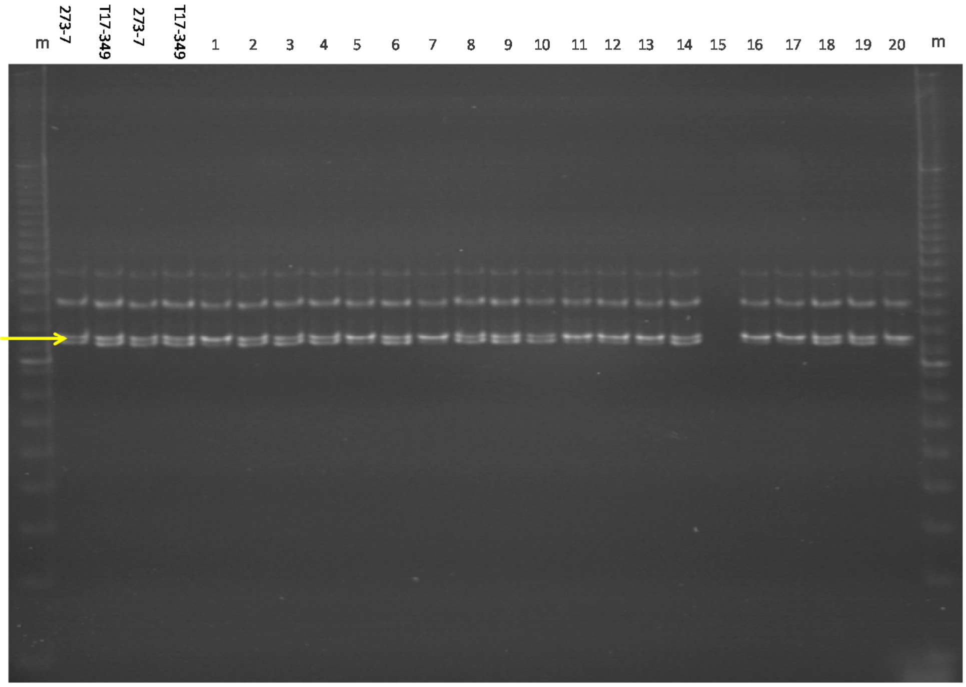

WCS0643

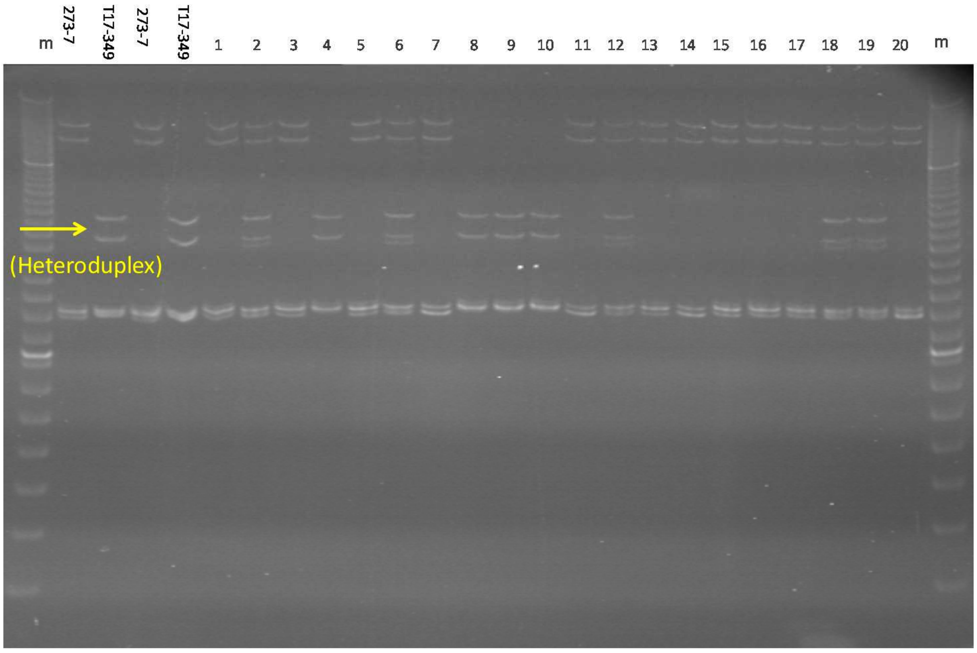

WCS1743a

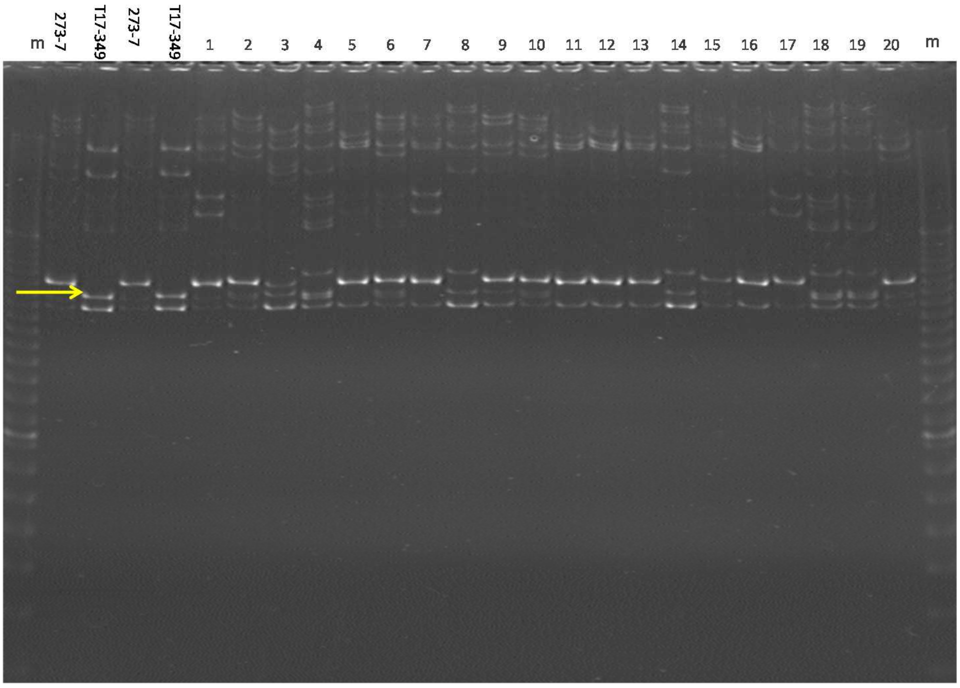

RCS6631b

m 273-7 T17-349 273-7 T17-349 1 2 3 4 5 6 7 8 9 10 11 12 13 14 15 16 17 18 19 20 m

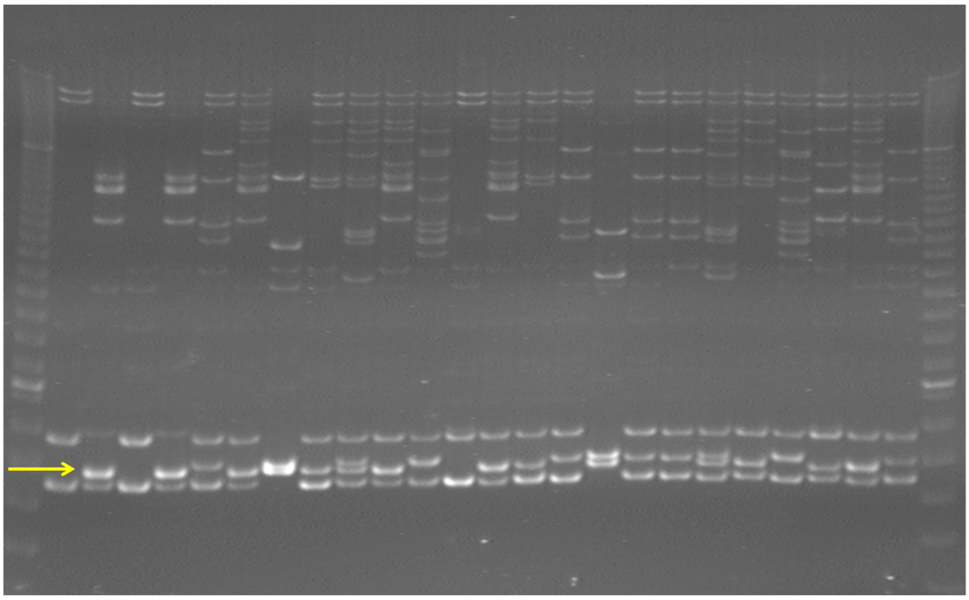

WCS0467b

m 273-7 T17-349 273-7 T17-349 1 2 3 4 5 6 7 8 9 10 11 12 13 14 15 16 17 18 19 20 m

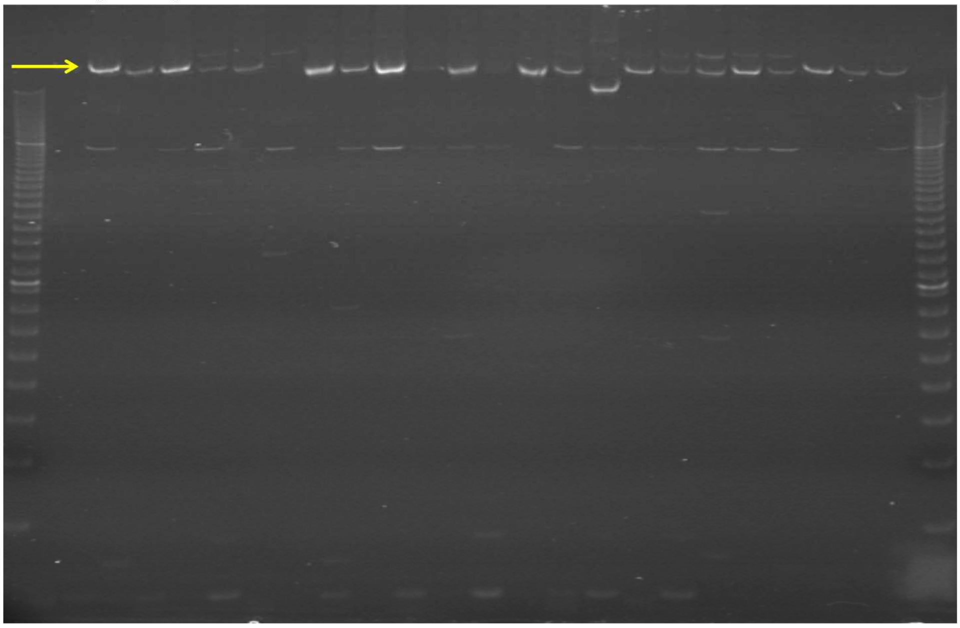

WCS0598a and WCS0598b

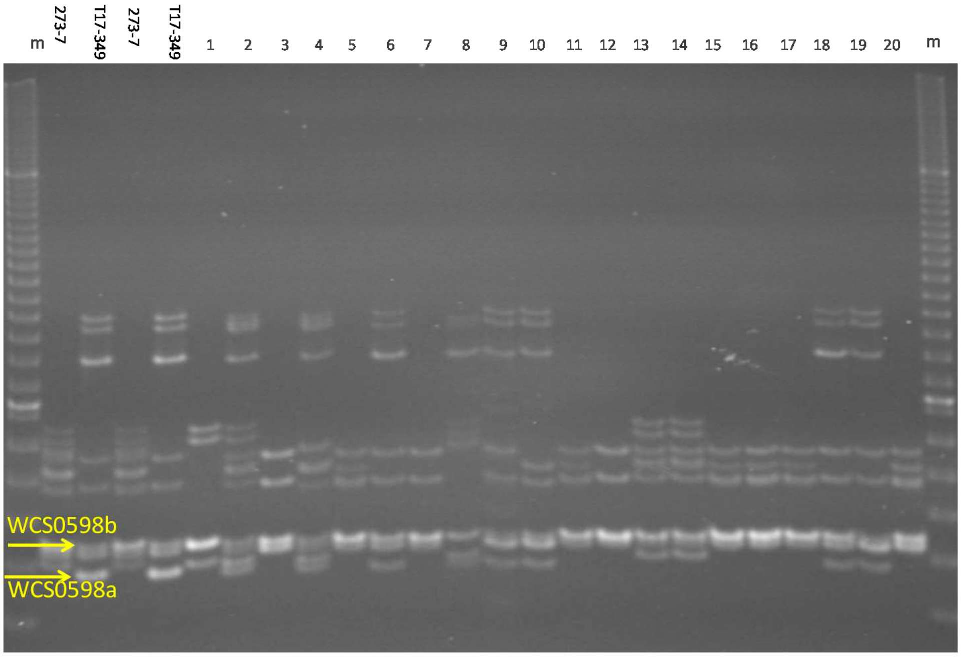

WCS1122a

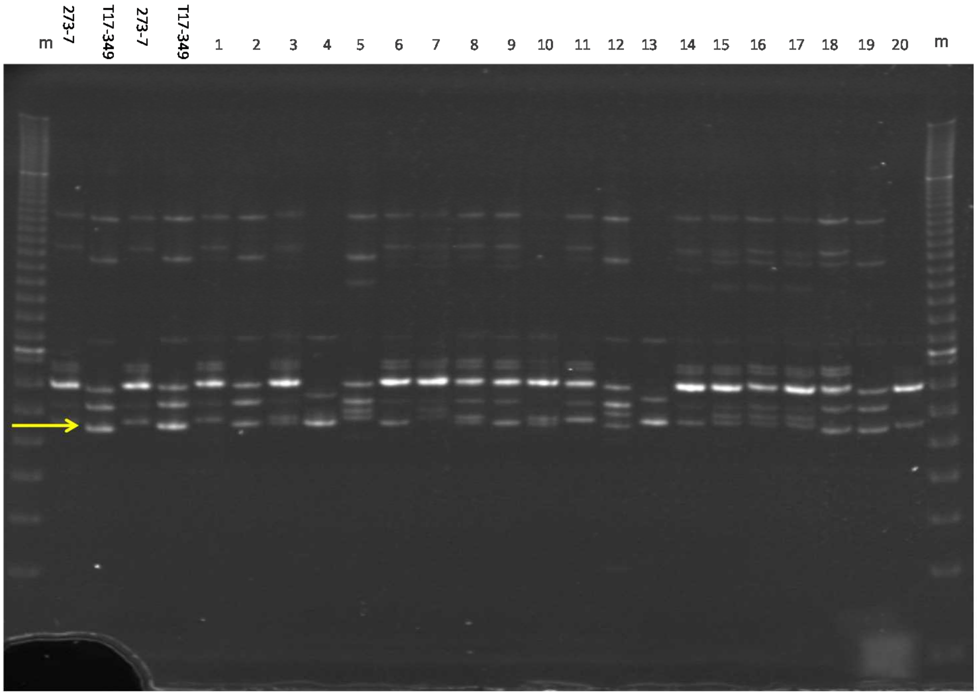

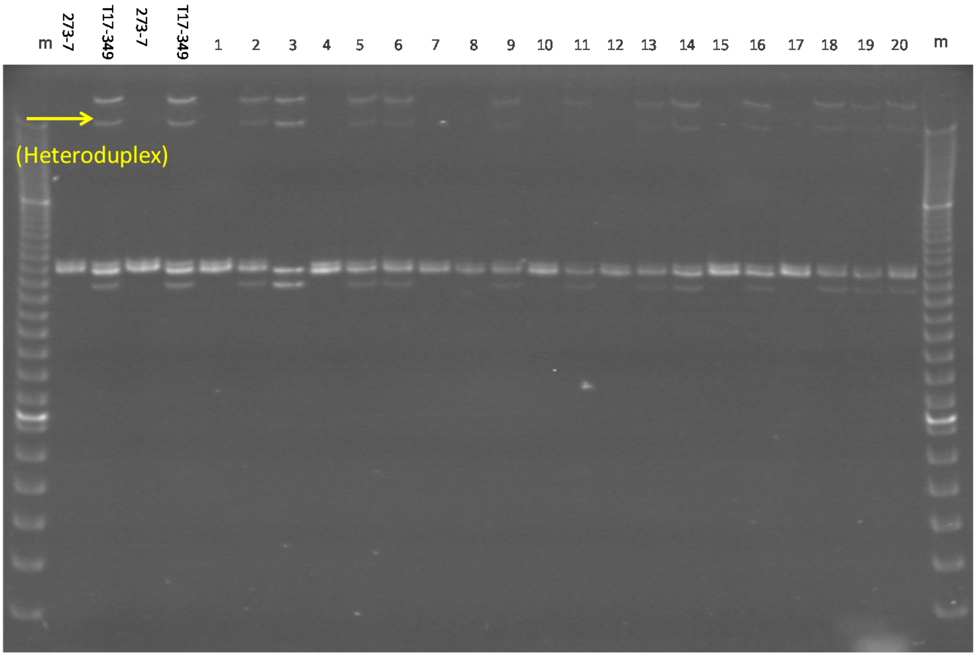
WCS0975
m
273-7
T17-349
273-7
T17-349
1 2 3 4 5 6 7 8 9 10 11 12 13 14 15 16 17 18 19 20
m
(Heteroduplex)

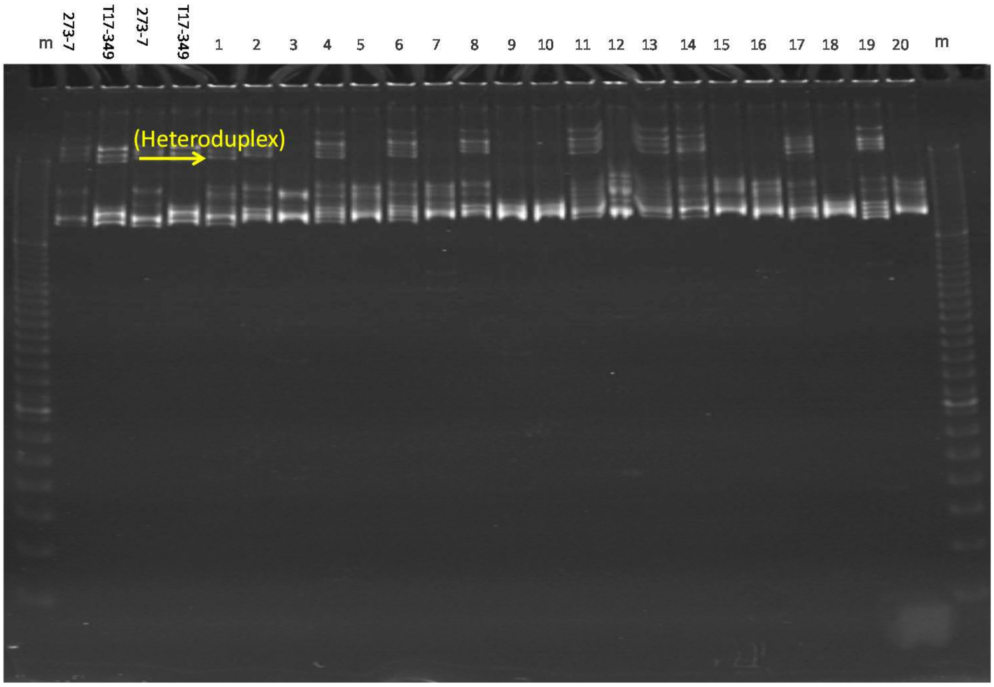
RCS4458
m
273-7
T17-349
273-7
T17-349
1 2 3 4 5 6 7 8 9 10 11 12 13 14 15 16 17 18 19 20
m
(Heteroduplex)

ats032

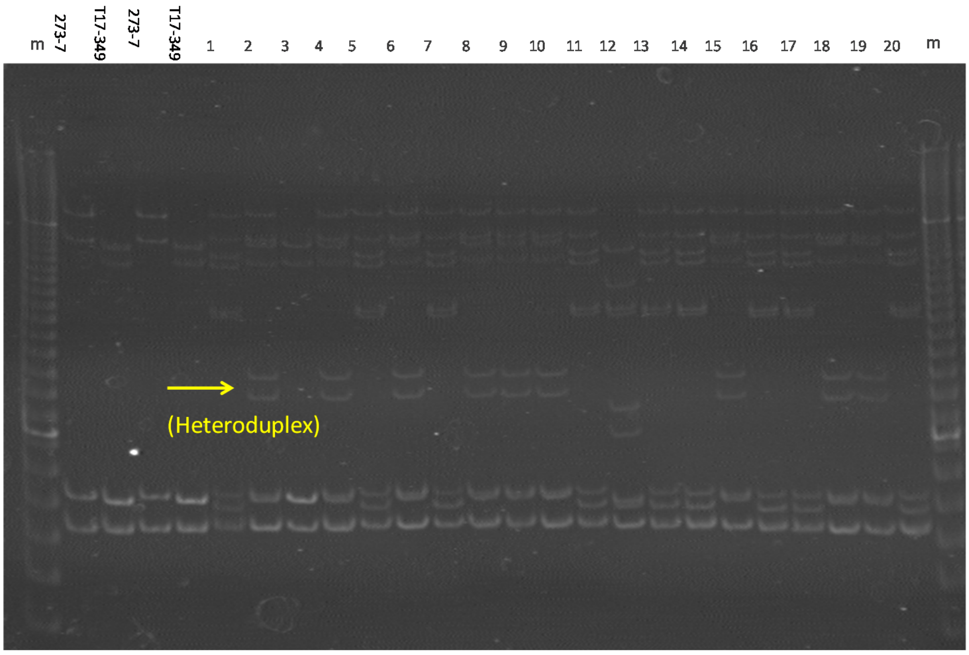

AW199a

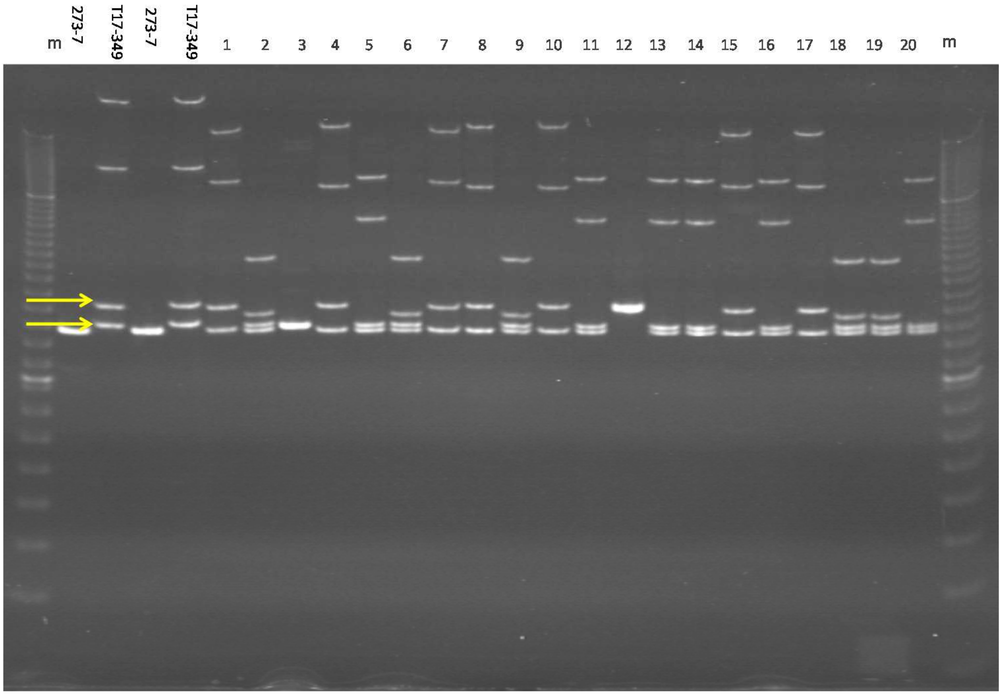

RCS4714

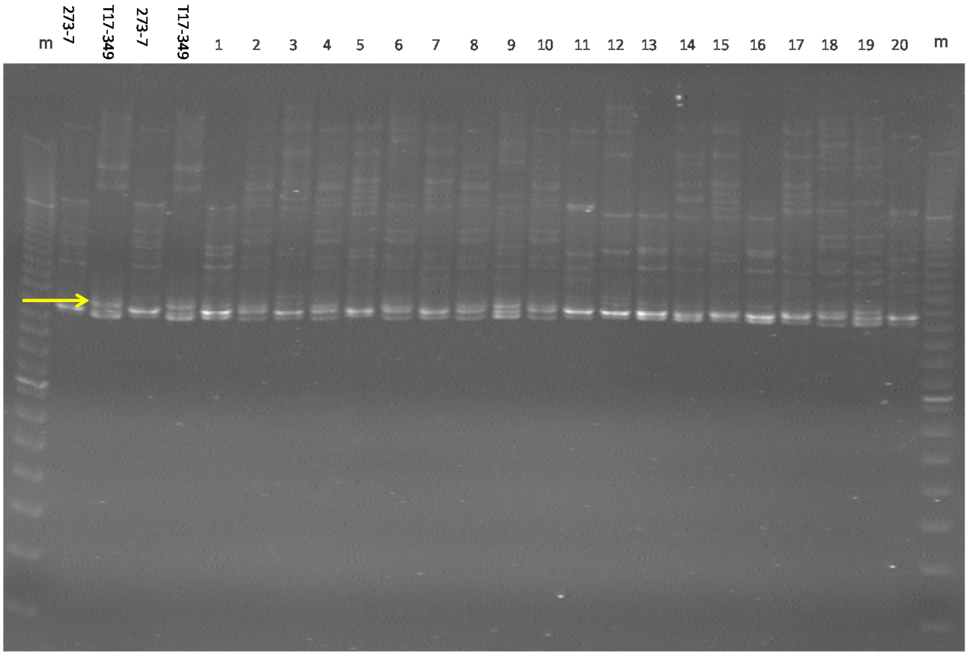

WCS0064

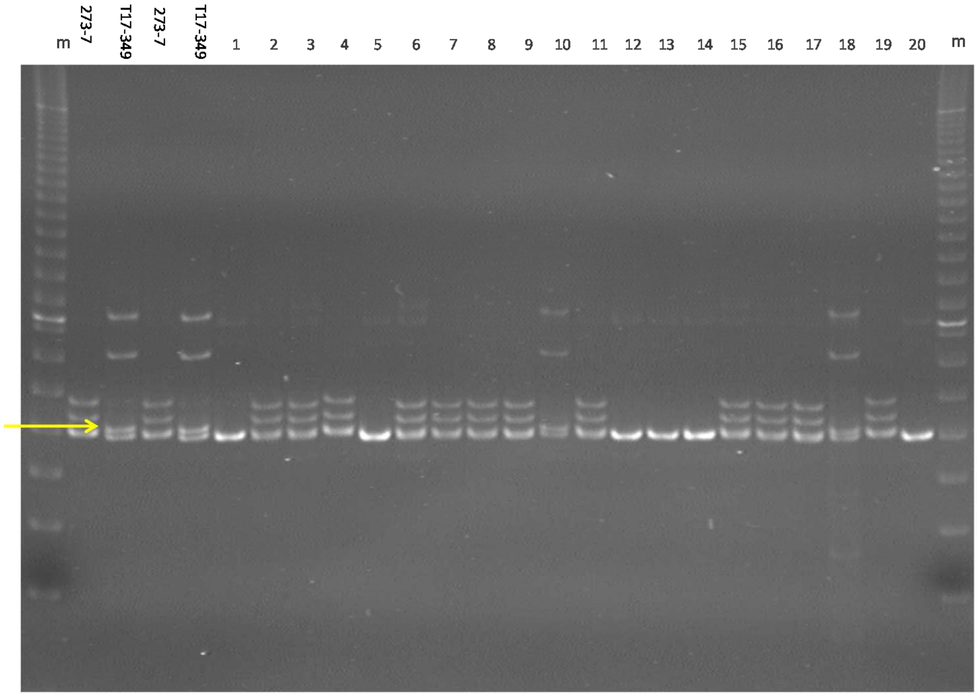

WCS0080c

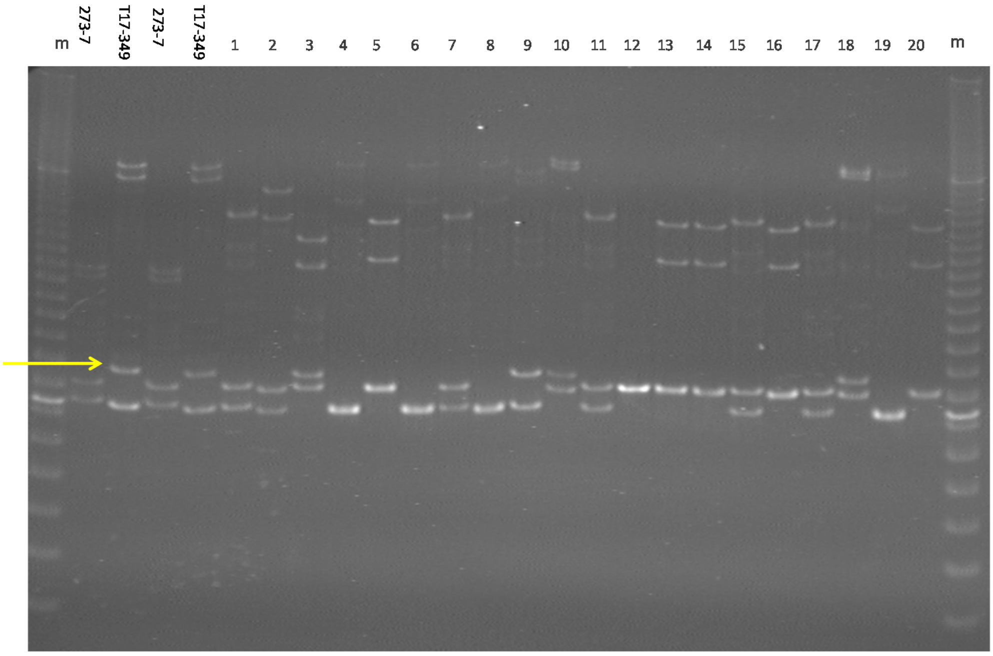

WCS1870b

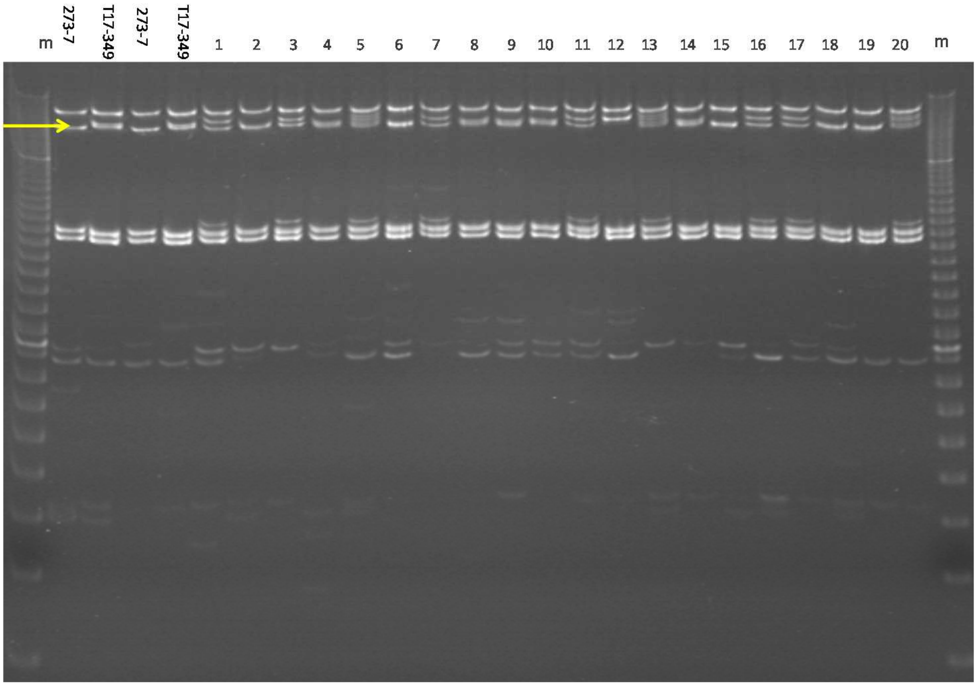

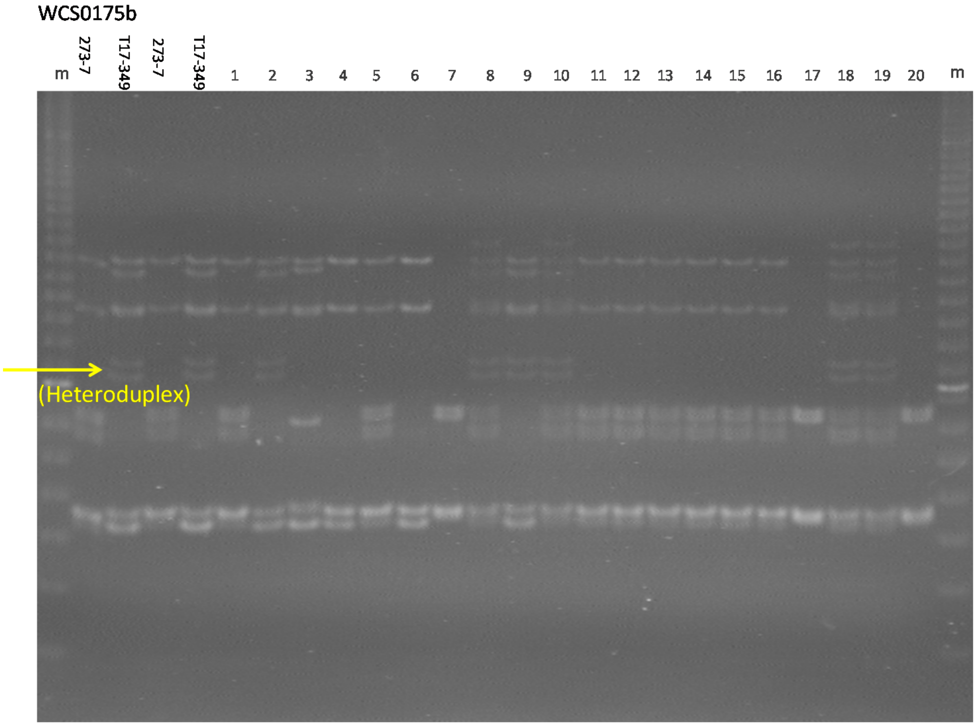

Figure S5 List of loci with identified LD showing r^2>0.5 between loci mapped onto different linkage groups, and photographs of acrylamide gel electrophoresis of PCR amplicons of the listed loci. The numbers above the acrylamide gel lanes represent IDs of F_1 individuals; m represents 20 bp ladder markers. Yellow arrows indicate scored bands for locus genotyping. 'Heteroduplex' shows bands used for scoring loci but predicted as heteroduplex bands.
